# Tyrosine Phosphorylation Profiling in FGF-2 Stimulated Human Embryonic Stem Cells

**DOI:** 10.1371/journal.pone.0017538

**Published:** 2011-03-17

**Authors:** Vanessa M. Y. Ding, Paul J. Boersema, Leong Yan Foong, Christian Preisinger, Geoffrey Koh, Subaashini Natarajan, Dong-Yup Lee, Jos Boekhorst, Berend Snel, Simone Lemeer, Albert J. R. Heck, Andre Choo

**Affiliations:** 1 Stem Cell Group, Bioprocessing Technology Institute, Agency for Science, Technology and Research (A*STAR), Singapore, Singapore; 2 Centre for Life Sciences (CeLS), NUS Graduate School for Integrative Sciences and Engineering (NGS), Singapore, Singapore; 3 Biomolecular Mass Spectrometry and Proteomics Group, Bijvoet Center for Biomolecular Research and Utrecht Institute for Pharmaceutical Sciences, Utrecht University, Utrecht, The Netherlands; 4 Netherlands Proteomics Centre, Utrecht, The Netherlands; 5 Bioinformatics, Department of Biology, Faculty of Science, Utrecht University, Utrecht, The Netherlands; 6 Centre for Biomedical Genetics, Utrecht, The Netherlands; 7 Division of Bioengineering, Faculty of Engineering, National University of Singapore, Singapore, Singapore; University of Southern California, United States of America

## Abstract

The role of fibroblast growth factor-2 (FGF-2) in maintaining undifferentiated human embryonic stem cells (hESC) was investigated using a targeted phosphoproteomics approach to specifically profile tyrosine phosphorylation events following FGF-2 stimulation. A cumulative total number of 735 unique tyrosine phosphorylation sites on 430 proteins were identified, by far the largest inventory to date for hESC. Early signaling events in FGF-2 stimulated hESC were quantitatively monitored using stable isotope dimethyl labeling, resulting in temporal tyrosine phosphorylation profiles of 316 unique phosphotyrosine peptides originating from 188 proteins. Apart from the rapid activation of all four FGF receptors, trans-activation of several other receptor tyrosine kinases (RTKs) was observed as well as induced tyrosine phosphorylation of downstream proteins such as PI3-K, MAPK and several Src family members. Both PI3-K and MAPK have been linked to hESC maintenance through FGF-2 mediated signaling. The observed activation of the Src kinase family members by FGF-2 and loss of pluripotent marker expression post Src kinase inhibition may point to the regulation of cytoskeletal and actin depending processes to maintain undifferentiated hESC.

## Introduction

Human ESCs are a powerful tool for drug screening, studying early lineage differentiation *in vitro*, and generating specific cell phenotypes for therapeutic applications. However, optimizing the efficient expansion and differentiation of these cells still requires further understanding, especially of the signaling pathways responsible for regulating hESC. Several pathways have been implicated in hESC self-renewal, including transforming growth factor-β/Activin-A/Nodal [1], sphingosine-1-phosphate/platelet-derived growth factor (S1P/PDGF) [2], insulin growth factor (IGF)/insulin [3] and fibroblast growth factor-2 (FGF-2) [4] (reviewed in Avery *et. al*. 2006 [5]). The process of self-renewal appears to be regulated synergistically through various pathways via growth factor or cytokine supplementation. Interestingly, FGF-2 signaling appears indispensible to hESC self-renewal just as leukemia inhibitory factor is to mESC [6]. Therefore, FGF-2 is widely used for sustained long-term culture of human embryonic stem cells (hESC) and induced pluripotent stem (iPS) cells under both feeder and feeder–free culture conditions [7]–[9].

FGFs execute their biological actions by activating cell surface fibroblast growth factor receptors (FGFRs) [10], [11]. The four human FGFRs, namely FGFR1, 2, 3 and 4, are members of the receptor tyrosine kinase (RTK) family. These receptors govern a wide variety of cellular processes from cell motility and differentiation to proliferation. hESC express all four FGFR [4], [12]–[15] whereby blocking of FGFR signaling leads to rapid differentiation [4], [12]. This suggests that FGF-mediated signaling is important for hESC self-renewal. Following FGF-2 stimulation, activation of the FGF/FGFRs in hESC typically results in signal transduction of the FGF canonical pathways, namely the mitogen activated protein kinase (MAPK) and phosphoinositide 3- kinase (PI3-K) pathways [4], [12], [16]. Further downstream signaling events upon FGF-2 stimulation and its link to hESC self-renewal and the maintenance of pluripotency remain to be determined.

The large scale analysis of cellular phosphorylation by liquid chromatography-mass spectrometry (LC-MS) is challenging due to the low stoichiometry of phosphorylation, causing phosphopeptides to remain largely undetected in the overwhelming background of non-modified peptides. Several of these large scale proteomic methods have been applied to profile phosphorylation in hESC [17]–[19]. Although impressive in the number of reported sites, these latter studies largely failed to monitor tyrosine phosphorylation. Therefore, specific enrichment of tyrosine phosphorylated peptides by phosphotyrosine-specific antibodies may provide a more targeted approach to study tyrosine phosphorylation [20]–[22].

**Figure 1:**
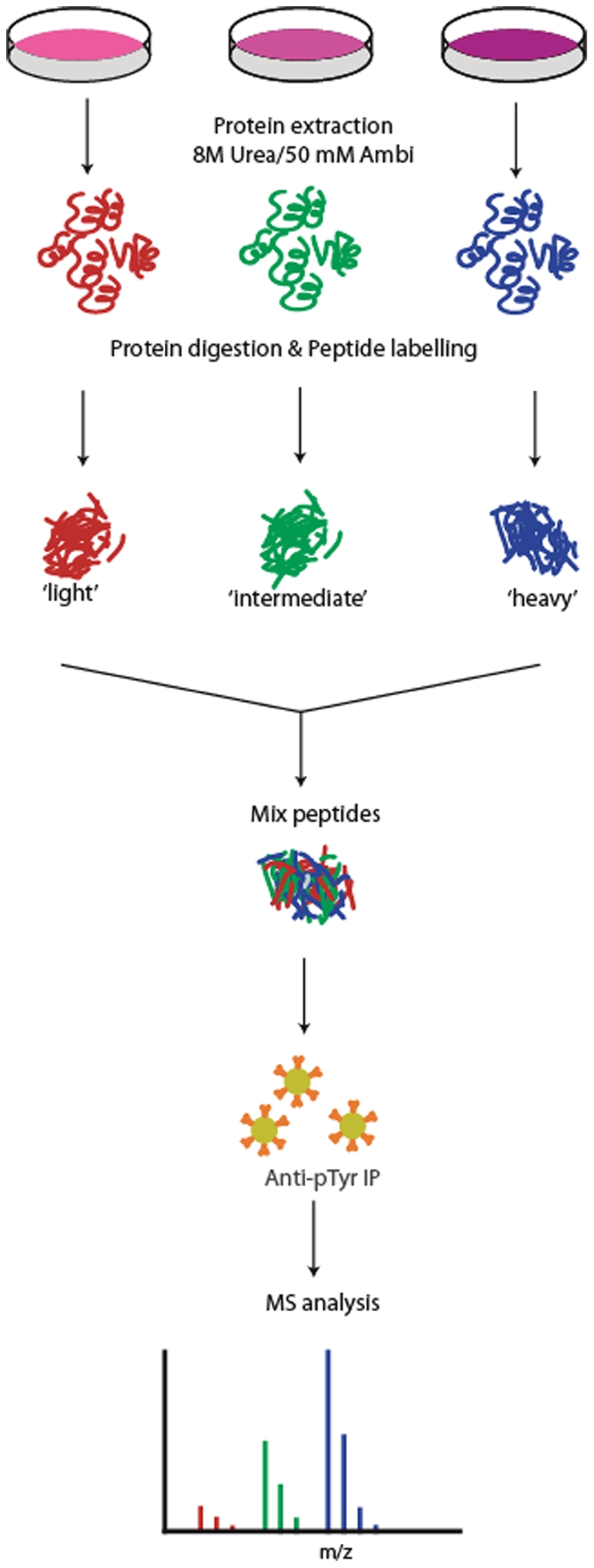
Overview of the quantitative proteomics workflow. Samples are stimulated for different times with FGF-2 followed by lysis and digestion. The peptides are differentially labeled by stable isotope dimethyl labeling and combined followed by simultaneous enrichment of tyrosine phosphorylated peptides using a phosphotyrosine specific antibody. The enriched fraction is analyzed by LC-MS.

To understand the broader implications of FGF signaling in hESC, we adopted a large-scale, targeted, phosphoproteomics approach to investigate tyrosine phosphorylation events following FGF-2 stimulation. Using a peptide-centered immuno-affinity purification strategy [22], [23] 735 unique tyrosine phosphorylation sites on 430 proteins were detected in two biological hESC replicates. Combining this enrichment technique with a stable isotope dimethyl labeling strategy [23], [24], a quantitative picture of the early signaling events in FGF-2 stimulated hESC was generated. Fig. 1 represents an overview of the quantitative phosphotyrosine proteomics strategy. Results from our quantitative dataset suggest that all four FGFRs were activated upon FGF-2 stimulation. Induced tyrosine phosphorylation of members of both MAPK and PI3-K pathways was also observed. Additionally, diverse trans-activation of receptors in the EGF family (EGFR, ERBB2, and ERBB3), Insulin family (IGF1-R and INSR), Ephrin receptors, and Vascular endothelial growth factor receptor 2 (VEGFR2/KDR) was detected. An increase in phosphorylation of Src kinase substrates was also observed, suggesting a possible role of FGF-2 in regulating cytoskeletal and actin dependent processes. The impact of changes to the cytoskeletal processes on hESC pluripotency should be further explored.

## Results

### Effect of FGF-2 stimulation on the hESC phosphoproteome

To specifically investigate the effects of activating FGFR, exogenous FGF-2 was added to hESC that were deprived of FGF-2 for 5 days (1 day ∼ 1 population doubling). Activation of the hESC was validated by Western blotting using two antibodies specific for the phosphorylation of proteins downstream of the FGFR. After 15 min of stimulation, a rapid upregulation of both phosphorylated ERK1/2 (ph-ERK1/2) and AKT (ph-AKT) was observed (Fig. S1).

A targeted phosphoproteomics experiment was subsequently carried out to profile the global tyrosine phosphorylation events post FGF-2 stimulation using an anti-pY specific antibody to enrich for tyrosine phosphorylated peptides [22], [25]. A total of 6 mg of cell lysate per cell state (different time points following FGF-2 stimulation) was digested with trypsin and this peptide digest was subsequently enriched for tyrosine phosphorylated peptides. The enriched peptides were then analyzed by LC-MS. A total of 153, 376, 273, 285 and 287 unique tyrosine phosphorylation sites could be identified respectively from the 0, 1, 5, 15 and 60 min post-FGF-2 stimulated cells. A cumulative total of 597 unique tyrosine phosphorylation sites were observed. In a biological replicate performed with similar amounts of sample and with similar FGF-2 stimulation conditions, a total of 574 tyrosine phosphorylation sites could be identified. Both experiments led to an overall cumulative 735 unique tyrosine phosphorylation sites in the two biological replicates (Table S1). The observed more than double increase in tyrosine phosphorylation immediately after FGF-2 stimulation suggests a prompt activation of numerous tyrosine phosphorylation signaling pathways. The 76% overlap we observed between the here performed biological replicates is much larger than typically observed in shotgun LC-MS approaches focusing on global serine and threonine phosphorylation [26]. This is likely caused by the significant reduction in sample complexity after enrichment of tyrosine phosphorylated peptides, thus allowing a more comprehensive and reproducible profiling of tyrosine phosphorylation [22]. A first classification using Panther [27] indicated that many of the detected pY sites were on proteins involved in (growth factor) signaling, such as the expected FGFs, but also EGF, VEGF and PDGF, and proteins involved in their downstream pathways.

**Figure 2:**
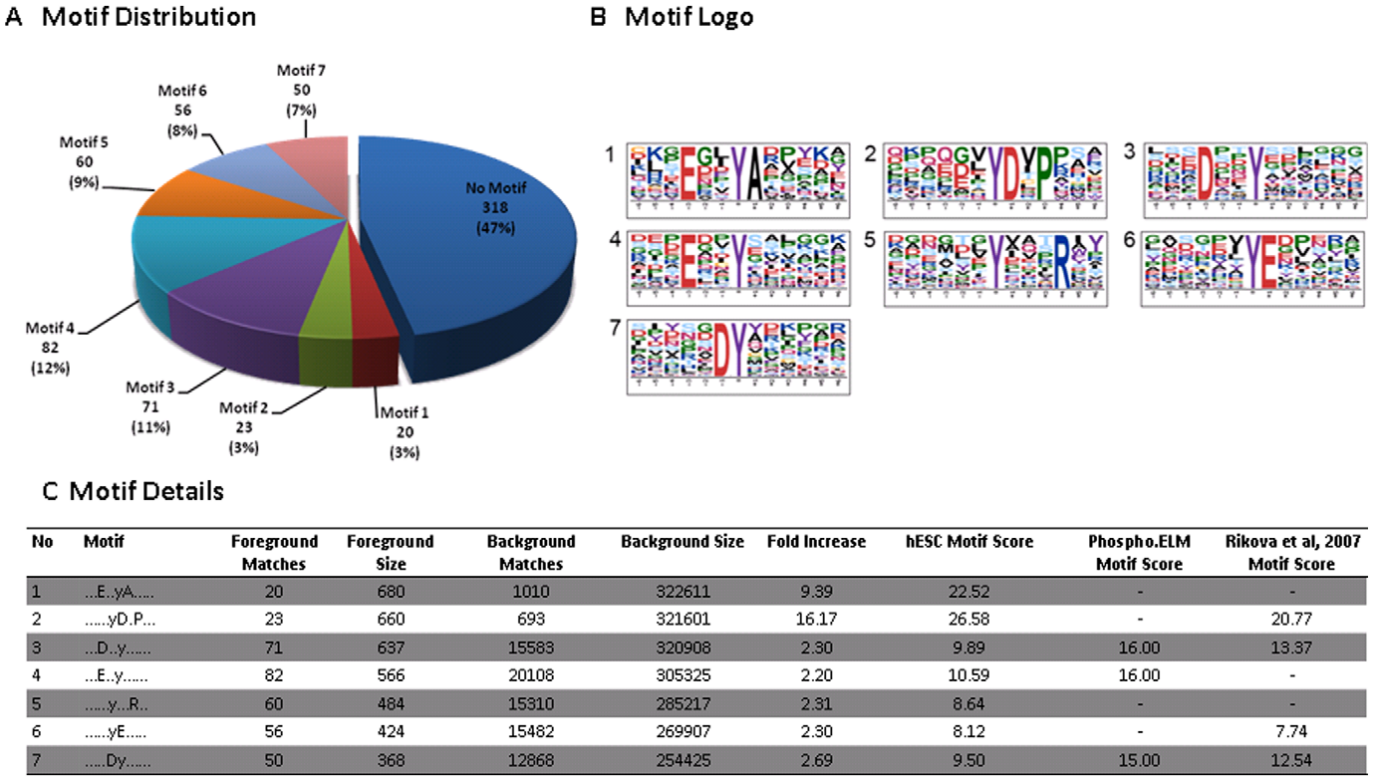
Tyrosine phosphorylation motifs overrepresented in the hESC cumulative dataset. Seven motifs were found to be significantly overrepresented using the Motif-X algorithm. (A) Distribution of different motifs in the dataset. For approximately half of the phospho-peptides (53%) an overrepresented motif could be identified, with the most abundant motifs being motifs 3 (12%) and 4 (11%). (B) Sequence logos of the various motifs. (C) Comparison of motifs among the various datasets. The table shows the motifs found in the different datasets, and their respective motif score. Motifs 2, 3, 4, 6, and 7 were also found either in the Phospho.ELM (ver. 8.2) or the Rikova et.al [*25*] datasets. Motifs 1 and 5, however, are unique to the hESC dataset. The number of foreground matches, background matches and the level of fold increase for each motif are also shown in the table.

A comparison of our hESC dataset with known pY site locations obtained from Phospho.ELM (version 8.2) and Rikova et al. [25] yielded overlaps of only 23% and 41%, respectively (Fig. S2). A global motif analysis was performed on the residues adjoining the phosphorylation sites in our hESC dataset. Overrepresented sequence motifs were extracted using Motif-X [28]. Employing the human IPI database for background sequences, 7 significant motif patterns could be identified, covering over 53% of our dataset (Fig. 2A–B). Comparison of the seven motif patterns identified in hESC with both the phospho.ELM database and the Rikova data revealed that motif 1 and motif 5 are unique to our hESC dataset. Amongst the proteins exhibiting such motifs were PI3-K (in motif 1), MAPK1, MAPK3, GAB1, SHB, ERBB3, TJP1, PKP2, PKP4, and CDK5 (all in motif 5) (Table S2). Further comparison to the Human Protein Reference Database [28] confirmed that both motif 1 and motif 5 are indeed unique to our cumulative hESC dataset (Fig. 2C).

### Quantitative phosphoproteomics

**Figure 3:**
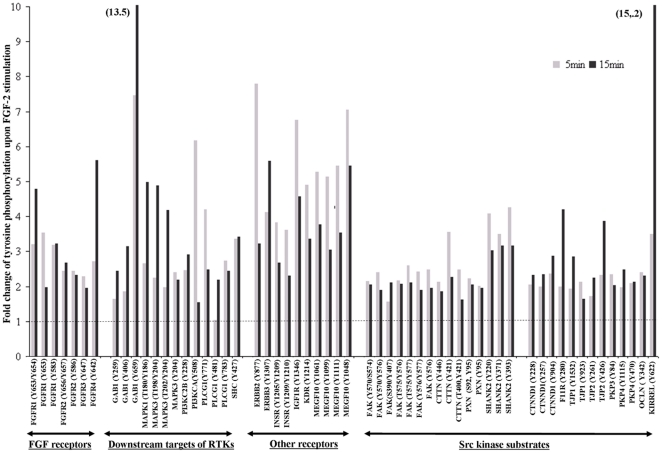
Quantitative temporal profiles of site-specific tyrosine phosphorylation upon FGF-2 stimulation for a selection of proteins. Data is normalized to no FGF-2 stimulation (0 min, dashed line).

**Figure 4:**
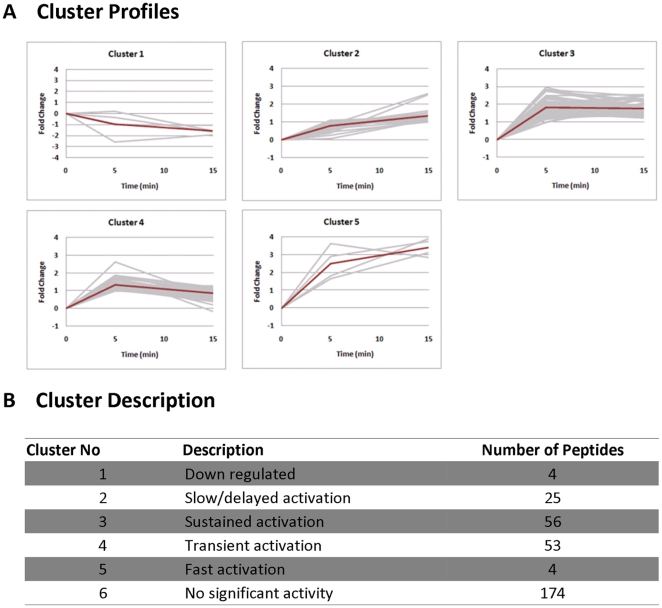
Clustering of temporal tyrosine phosphorylation profiles in response to FGF-2 stimulation. (A) The clusters. The grey lines show the profiles of the individual peptides within that cluster. The shapes that define the characteristics of each cluster are shown as thick red lines in the graph. (B) The qualitative description of each cluster and the number of peptides. The 174 peptides that display less than 2-fold change in activity are excluded from the clustering and are instead grouped into cluster 6 (no significant activity).

In order to accurately quantify the tyrosine phosphorylation events, we next applied a combined approach of pY-peptide immunoprecipitation (IP) and stable isotope labeling. Using this combined approach, we have generated a more precise and quantifiable tyrosine phosphorylation profiles [22]. Our Western blot results on ERK1/2 and AKT (Fig. S1) indicated that most of the tyrosine phosphorylation events occur within 15 min of FGF-2 stimulation. Therefore, 0, 5 and 15 min post FGF-2 stimulation time points were chosen for this profiling study. hESC lysate for each time point (6 mg) was digested, followed by stable isotope dimethyl labeling, whereby the non-stimulated hESC (0 min) was labeled with light dimethyl labels, the 5 min time point with intermediate labels, and the 15 min time point with heavy dimethyl labels. The differentially labeled samples were mixed 1∶1∶1 and enriched simultaneously for pY peptides by IP. The enriched pY peptides were then analyzed by LC-MS, using a 3 h elution gradient. By comparing their signal intensities, peptides from the different FGF-2 stimulated time points could be relatively quantified. From our analysis, 316 unique pY-peptide triplets (light, intermediate, heavy) with 300 unique tyrosine phosphorylation sites were identified (Table S3). The quantitative analysis demonstrated an increase in tyrosine phosphorylation of all 4 FGFRs and some of their canonical downstream effectors (eg. PLC-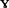, MAPK, PI3-K) following FGF-2 stimulation Fig. 3. Furthermore, a large number of pY-peptides not directly involved in the canonical FGF pathway were also identified with increased phosphorylation upon FGF-2 stimulation. These include Src kinase substrates (e.g. FAK, CTTN, PXN, SHANK2), additional receptor tyrosine kinases (e.g. INSR, IGF1R, KDR, ERBB2, MEGF-10), and many others (e.g. Occludin, TJP1, TJP2, Kirrel) (Fig. 3). To classify the global response to FGF-2, a cluster analysis was performed based on the temporal tyrosine phosphorylation profiles. Phosphopeptides that showed at least a 2-fold increase (arbitrarily chosen as a substantial increase) in tyrosine phosphorylation at either of the stimulated time points (5 min or 15 min) were clustered into 5 different groups (Fig. 4 and Table S4). Most of the pY phosphopeptides (approximately 34.5%) showed sustained or transient activation (Clusters 3 and 4) upon stimulation. The pY peptides of all FGFRs and some of their downstream targets (PLC--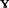, MAPK1, MAPK3, PI3-K, SHC) were found within these clusters.

Interestingly, various substrates of the Src kinase family were also found within these clusters, namely Shank2, F11R, PKP2, TJP2, CDK5 (all in cluster 3), and CTTN, OCLN, FAK, PKP3, PXN, and TJP1 (all in cluster 4) (see Table S4). The Src family kinases have been implicated in the maintenance of stem cell pluripotency[29]. Selective inhibition of these kinases by the chemical inhibitor SU6656 resulted in the downregulation of expression of such pluripotent marker as Oct3/4 and Nanog and decreased growth of hESCs[29]. Similar results were also observed in our study (Fig. S3A–C). Despite the continuous stimulation of hESC with FGF-2, inhibition of Src kinases with 1 µM SU6656 for 6 days led to significant downregulation of Oct3/4, Nanog and EpCAM expression, suggesting the importance of Src kinase signaling in maintaining the undifferentiated hESC phenotype. Concomitantly, decrease in cell numbers was observed following SU6656 treatment, however, this was more pronounced at 4 µM (Fig. S3B).

**Figure 5:**
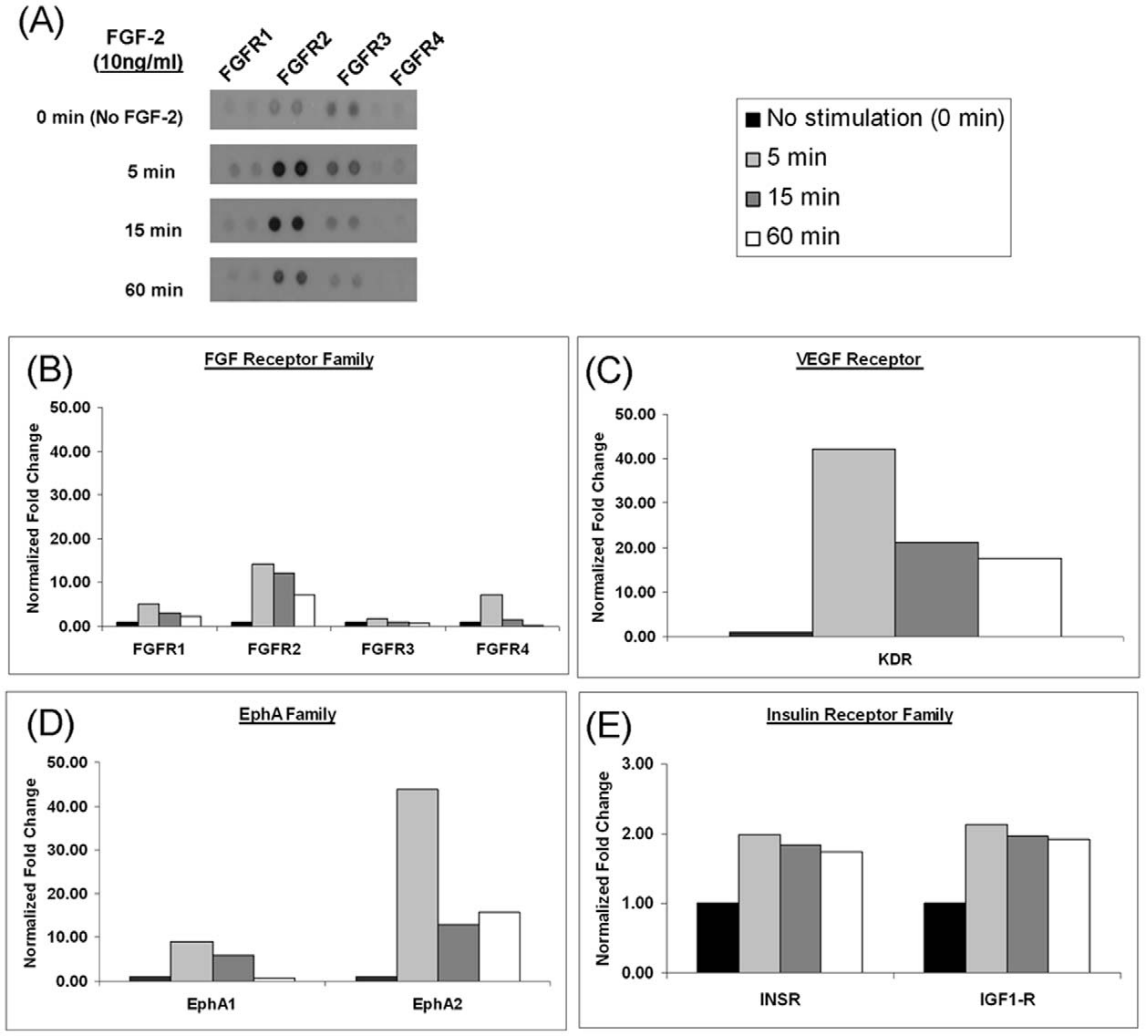
Human phospho-RTK array to detect proteins activated upon FGF-2 stimulation. (A, B) Phosphorylation levels of FGFR 1, 2, 3, and 4 are increased upon FGF-2 stimulation. (C, D, E) Proteins in the insulin receptor, ephrin A, and VEGFR family show an increase in phosphorylation upon FGF-2. All densitometry results were normalized to cells that had no FGF-2 stimulation (0 min).

To confirm the increased phosphorylation of the RTKs identified post FGF-2 stimulation, a protein array containing 42 different human RTKs (including receptors from the FGF, EGF, VEGF, and insulin receptor families) was used (Fig. 5). In the array experiment, we also included an additional time point (60 min) to understand the pattern of phosphorylation in stimulated hESC. A rapid increase in phosphorylation of all FGF receptors was observed (Fig. 5A–B). Furthermore, phosphorylation levels for INSR, IGF1-R, EphA 1 and 2, VEGFR2/KDR were also increased as compared to the basal level (no stimulation). (Fig. 5C–F). Results from the array confirmed an elevated level of phosphorylation in several RTK, and all four FGFRs (FGFR1–4) were activated simultaneously upon FGF-2 treatment.

## Discussion

Many cytokines and growth factors have been shown to play a role in maintaining self-renewal of hESCs (reviewed in [5], [30]). The central role of FGF-2 in maintaining hESC self-renewal remains undisputable, as almost all hESC culture platforms require FGF-2 supplementation. Withdrawal of exogenous FGF-2from hESC results in the loss of pluripotent marker expression (Oct-3/4, Tra-1-60, and Podocalyxin) [12]. Furthermore, blocking FGFR signaling in hESC leads to rapid differentiation [4], [12]. Interestingly, co-localization of all four FGFRs occurs on Oct-3/4 positive cells. Taken together, these results suggest that FGF-mediated signaling is important for the maintenance of the undifferentiated hESC phenotype. To further understand FGF-2 mediated FGFR signaling in hESC, we profiled tyrosine phosphorylation events following FGF-2 stimulation.

The main challenge in tyrosine phosphoproteomics is the low abundance of tyrosine phosphorylated proteins. In this study, we used a pY-specific antibody to enrich for these low abundant pY-peptides from large quantities of complex samples. Using this approach, 735 unique tyrosine phosphorylation sites in the two biological replicates were identified. To our knowledge, this is to date the largest tyrosine phosphorylation dataset reported for a hESC phosphoproteome. The overlap of our cumulative dataset with the datasets from Phospho.ELM and Rikova *et. al*. [25] is 23% and 41% respectively. Dissimilar cell constitutions (human/mouse cell lines/tissues for phosho.ELM, lung cancer cell lines/tissues from Rikova) together with different enrichment strategies could account for this difference. It has been estimated that tyrosine phosphorylation accounts for about 1% of all phosphorylated events in human cells [31], hinting at potentially over ∼75,000 phosphorylation events in our hESC. This is still an order of magnitude higher than currently achievable in large-scale phosphoproteomics screens [17], [32].

We identified, using Motif-X [33], a total of 7 tyrosine phosphorylated sequence motif. Interestingly, motif 1 and motif 5 have not been previously reported in any pY dataset, and could be specifically enriched in hESC. The hESC unique motif 5 has an arginine residue at the P+3 position, and was detected on proteins including MAPK1, MAPK3, GAB1, SHB, ERBB3, TJP1, PKP2, PKP4, and CDK5. Motif 1 contains a glutamic acid residue at P-2, and an alanine residue at P+1 position. A phosphopeptide of the regulatory subunit of PI3-K has a peptide sequence that belongs to motif 1, which is of interest as the PI3-K signaling pathway has been reported to be important for hESC self-renewal [12], [34]. The position of the glutamic acid at P-2 was also observed in motif 4. Hence, it is possible that motif 1 represents a subset of motif 4. Src kinase has been proposed to be the upstream kinase recognizing motif 4 [33]. The close resemblance between motifs 1 and 4 may imply that proteins/peptides belonging to motif 1 are also phosphorylated by Src kinases.

### Quantitative profiling of tyrosine phosphorylation

In order to understand early key signaling events of FGF-2 stimulated hESC, it is important to study the temporal involvement of the FGFRs, other RTKs, and their subsequent substrates post FGF-2 stimulation. Results from Western blotting of FGF signaling downstream effectors (ph-ERK1/2 and ph-AKT) demonstrated rapid response (5–15 min) of these downstream effectors to FGF-2 stimulation, hence we selected 0, 5, and 15 min for a more in-depth quantitative study. Stable isotope dimethyl labeling method was selected to be used in conjunction with pY-peptide IP. By performing stable isotope labeling prior to IP, potential variation in the IP or LC-MS step is mitigated as sample handling and analysis is performed simultaneously for all samples. A potential downside is that up to 6 mg of material needs to be isotope labeled, necessitating a cost-effective labeling approach provided by reductive dimethyl labeling. Using the above combination, 316 unique quantifiable tyrosine phosphorylated peptides, occurring as a triplet in the MS data, could be identified and quantified.

As expected, increased tyrosine phosphorylation at multiple sites of FGFRs was observed after FGF-2 stimulation, notably on all four FGFRs. The use of the RTK array confirmed the simultaneous activation of the four FGFRs. A separate study by Livia *et. al* (2004) [35] using the RTK array also demonstrated activation of FGFRs in hESC by FGF-2 stimulation. Increases in tyrosine phosphorylation of downstream targets implied in canonical FGF signaling, such as PLC–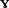, GAB1, MAPK1/3, PI3-K could also be detected. The temporal response of pY residues at the autophosphorylation sites on the FGFRs indicates propagation of the signal due to the presence of exogenous FGF-2.

Apart from FGFRs, several other receptors were found to be rapidly tyrosine phosphorylated following FGF-2 stimulation. These included INSR, IGF1-R, ERBB2, ERBB3, EphA1 and 2, EphB3 and 4, VEGFR2/KDR, and MEGF10. The activation of most of these receptors was confirmed by a human phospho-RTK array. IGF1-R and IR have been shown to play a role in self-renewal of hESC [3], [36]. Furthermore, tyrosine phosphorylation of ERBB2, ERBB3, VEGFR2/KDR, FGFR3, and FGFR4 has been demonstrated previously upon stimulation with conditioned medium supplemented with FGF-2 [36]. MEGF10 showed a noteworthy increase in tyrosine phosphorylation at multiple sites. There are very limited reports on MEGF10 and its function in hESC remains to be further explored (see Text S1 for further discussion). Although many of the results obtained in the pY-IP approach could be validated using the human phospho-RTK array, the sequencing approach of the phosphoproteomics method allows the determination of exactly which tyrosine sites are differentially phosphorylated and therefore provide higher resolution data.

One possible explanation for the induced tyrosine phosphorylation of other RTKs following FGF-2 stimulation could be transactivation [37], [38]. For example, FGFR1 has been shown to be capable of tyrosine phosphorylating EphA4 in an adult kinase-negative mutant cell line [37]. Also, it has been demonstrated that a transactivation mechanism might even be required to establish certain physiological processes [38]. The elucidation of these RTK transactivations and cross-talk between different pathways, once again, illustrates that a linear view of signal transduction is an oversimplification of the actual signaling process [18]. Some RTKs share several of the same downstream targets, including the above mentioned PLC–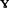, GAB1, MAPK1/3, and PI3-K, which may complicate the determination whether phosphorylation of any of these proteins is the direct result of upstream activation of FGFR or any of the other RTKs. Therefore, a more comprehensive view based on the data provided here is required to deconvolute the complex interplay of signaling pathways underlying the effect of cellular stimuli.

### Src family kinases and substrates

Src family kinases (SFKs) represent a group of tyrosine kinases that are strongly activated by, amongst others, RTKs [39]. Several tyrosine phosphorylation sites were identified on members of SFKs, but the extensive homology, in particular, in the activation loop of different members of the family complicates the determination of the exact family member. Interestingly, although only relatively small increases in tyrosine phosphorylation were observed for the SFK members, larger increases in tyrosine phosphorylation were observed for Src substrates, for example, focal adhesion kinase (FAK). Src and FAK are known to form a tight complex after activation by RTKs or integrins [40], [41]. Src binds to phosphorylated FAK and promotes further phosphorylation of tyrosine residues on FAK.

Cortactin has long been known to be a very efficient substrate for SFKs and was found in our screen to exhibit increased tyrosine phosphorylation levels. These phosphorylation events are supposed to create docking sites for proteins containing the SH2 phosphotyrosine binding domain, such as Src family kinases themselves or adaptor proteins [42], [43]. These interactions have been proposed to influence actin polymerization and thus the turnover of actin networks. SHANK2 was also found to be heavily phosphorylated upon induction with FGF. It is a large scaffold protein associated with actin, and also a binding partner of Cortactin [44]. Another protein that showed increased tyrosine phosphorylation is the scaffold protein Paxillin, which localizes at the sites of cell adhesions. Together with a large number of interaction partners, Paxillin is heavily involved in cytoskeletal reorganization and cell adhesion [45]–[48].

Plakophilin-3, Plakophilin-4, Catenin delta-1, F11R, Occludin, KIRREL/Neph1, TJP-1 and TJP-2 are found in tight junctions and an increased tyrosine phosphorylation was detected in our screen. Of these proteins Catenin delta-1, Occludin, TJP-1 and TJP-2 have been described as genuine Src kinase substrates; the phosphorylation of the aforementioned sites has been shown to modulate the regulation of cell-cell adhesions as well as the formation of tight junctions [45], [49], [50].

The prevalence of activity of Src family kinases can be seen from both Motif mapping (Motif 1 and 4) and the large number of Src kinase substrates identified. SFKs can also be recruited by receptor mediated phosphorylation and influence the signaling dynamics of the cell. It has been stated that Src activity controls FGFR activation and FGF-2 induced AKT activation is dependent on activation of Src [51]. Furthermore, SFKs are known to promote cell growth and survival by activation of MAP kinase pathways (e.g. MEKK2/5, Erk5) or the signal transducers and activators of transcription (STAT) family (e.g. STAT3). In this study, we demonstrate that Src kinase activity is crucial for the maintenance of pluripotency by FGF-2. Inhibition of Src kinases resulted in decreased growth and expression of markers representing the undifferentiated hESC state. Results from this investigation and previous study by Annaren *et. al*. (2004) suggest a considerable involvement of these kinases and their substrates in hESC in maintenance of undifferentiated hESC[29] .

### Other interesting targets

CDK5 was also found to be upregulated upon FGF-2 treatment. CDK5 is an atypical member of the cyclin dependent kinase family, which has rather been found to be involved in the regulation of CNS development [52]. Many of its substrates are also involved in cytoskeletal regulation and neuronal migration such as c-Abl, Src, PAK1 or β-catenin.

Multiple EGF-like domains 10 (MEGF10) was identified in the dataset with an average of 5 fold-change on all the four pY-peptides identified (within 5 min of FGF-2 stimulation). Out of the four pY-peptides identified, two were not reported previously. Additionally, this is the first account for identification of tyrosine phosphorylation of MEGF10 in hESC, but its precise function in hESC is largely unknown. MEGF10 is believed to be the mammalian ortholog of nematode CED-1, a cell surface receptor involved in phagocytosis of dead cells [53], [54]. It has been known to interact with clathrin assembly protein complex 2 medium chain (AP50) [55], and ATP binding cassette transporter (ABCA1) [54]. The function of this protein is largely thought to be involved in cell engulfment due to its similarity with CED-1 [54], [55]. It has also been reported to have a role in novel adhesion pattern, restricting cell motility, and inducing large vacuole formation [53], [55]. There are limited reports on the MEGF10 signaling pathway. Whether the function of the protein is supporting self-renewal of hESC remains to be elucidated.

### Conclusion

**Figure 6:**
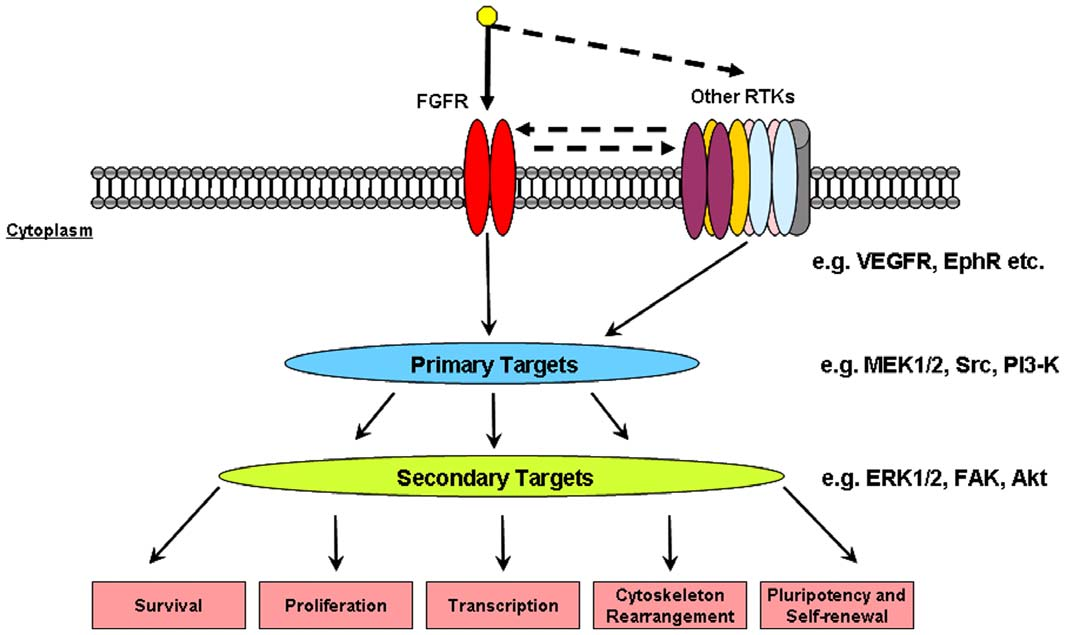
Summary of tyrosine phosphorylated RTKs and its downstream targets after FGF-2 stimulation in hESC culture. FGF-2 treatment of hESC results in the activation of all four FGFR family members as well as possibly transactivation of members of several other receptor families such as the insulin receptor family, the Ephrin type A family and the KDR receptor. Increased tyrosine phosphorylation was found on downstream substrate targets of these RTKs including Src kinase substrates.

In this study, we have provided a global temporal tyrosine phosphorylation profile of hESC after FGF-2 stimulation. In Figure 6, the events following the introduction of FGF-2 to hESC are summarized. Remarkably, we did not only observed an activation of the FGFR pathways, but also detected the tyrosine phosphorylation of several other RTKs upon FGF-2 stimulation. This activation could be either a direct effect of FGF-2 (although no specificity for FGF-2 is known for the mentioned RTKs), through transactivation, which would involved an FGFR, or via a yet unknown mechanism. All RTKs, including FGFRs, have downstream target substrates that are tyrosine phosphorylated. Not surprisingly, we found a plethora of proteins with increased levels of tyrosine phosphorylation upon FGF-2 stimulation, many of which have not been associated with FGF signaling before. Many of these proteins have been described as integral components and also key players in the aforementioned processes in adult cells with functions including modulation of cell-adhesion, cell-cell interaction, migration and the formation of tight junctions. Our preliminary results from our Src kinase inhibition experiment suggest possible involvement of the Src kinase and its substrate in the maintenance of undifferentiated hESC phenotype. Interestingly, changes to cytoskeletal and actin dependent processes are major events during hESC differentiation [56]. Therefore, we hypothesize that the pluripotency maintaining effect of FGF-2 in hESC might be partially executed through these processes.

## Materials and Methods

### Cell culture, stimulation, and cell lysis

Human ESC line, HES-3 (46 X,X) was obtained from ES Cell International (ESI, Singapore). Briefly, the cells were cultured on Matrigel-coated (Becton, Dickinson and Company, Franklin Lakes, NJ) tissue culture dishes and supplemented with conditioned medium from MEF. For routine culture, the medium was supplemented with 10 ng/ml of FGF-2 (Invitrogen, Carlsbad, CA, USA), and medium was changed daily. The cultures were passaged weekly following enzymatic treatment as previously described [57]. For FGF-2 starved cultures, cells were maintained in the absence of FGF-2 for 5–7 population doublings (PD, 1 PD  =  ∼1 day). Cells were then stimulated with 10 ng/ml of FGF-2 at the indicated time points.

Cells were lysed on ice in 7 M urea, 2 M thiourea, 4% CHAPS, 40 mM Tris, 50 µg/ml DNase, 50 µg/ml RNase, 1 mM sodium orthovanadate and 1X *PhosSTOP* (Roche Diagnostics, Switzerland, Rotkreuz, http://www.roche.com/) in the presence of protease inhibitors. Protein concentration was determined using Bradford Assay. Total protein lysate of 6 mg per time point were reduced with dithiothreitol (DTT) at a final concentration of 10 mM at 56°C. Subsequently, lysates were alkylated with 55 mM iodoacetamide. Lysates were diluted 6-fold with 100 mM ammonium bicarbonate and digested overnight with trypsin.

### Stable isotope labeling by reductive dimethylation of tryptic peptides

Tryptic peptides were desalted using a Sep-Pak C18 column (Waters, USA, Massachusetts), eluted peptides were lyophilized, and re-suspended in 100 µL of triethylammonium bicarbonate (100 mM). Subsequently, stable isotope dimethyl labeling was performed on the full digested lysate (∼6 mg) as described before [22] using formaldehyde-H_2_ and cyanoborohydride, formaldehyde-D_2_ and cyanoborohydride and formaldehyde-^13^C–D_2_ and cyanoborodeuteride to generate light, intermediate and heavy dimethyl labels, respectively. The light, intermediate and heavy dimethyl labeled samples were mixed in 1∶1∶1 ratio based on total peptide amount, determined by analyzing an aliquot of the labeled samples on a regular LC-MS run and comparing overall peptide signal intensities.

### Immunoprecipitation (IP) of phosphopeptides

Prior to LC-MS analysis, the differentially labeled peptides from the three different digested lysates were mixed, desalted with Sep-Pak C18 column, and lypholized. Labeled peptide mixtures were dissolved in IP buffer containing 50 mM Tris (pH 7.4), 150 mM NaCl, 1% NOG, and 1x complete mini protease inhibitor cocktail (Roche diagnostics). Agarose-conjugated anti-p-Tyr (pY99) antibodies (Santa Cruz Biotechnology Inc., USA, CA) (prewashed three times with IP buffer) were added into each peptide mixture and incubated overnight at 4°C with gentle rotation. After incubation, the beads were then washed three times with 1 ml of IP buffer followed by two times with 1 ml of water, all at 4°C. Peptides were eluted with 0.15% TFA and centrifuged at 1500 g for 1 min to separate the antibody beads from the eluate. Eluted peptides were desalted and concentrated on STAGE-tips.

### On-line nanoflow LC-MS

Nanoflow LC-MS/MS was performed by coupling an Agilent 1100 HPLC system (Agilent Technologies, Waldbronn, Germany) to a LTQ-Orbitrap mass spectrometer (Thermo Electron, Bremen, Germany) as described previously [58]. Dried fractions were reconstituted in 10 µL 0.1 M acetic acid and delivered to a trap column (Aqua^tm^ C18, 5 µm, (Phenomenex, Torrance, CA, USA); 20 mm × 100 µm ID, packed in-house) at 5 µL/min in 100% solvent A (0.1 M acetic acid in water). Subsequently, peptides were transferred to an analytical column (ReproSil-Pur C18-AQ, 3 µm, Dr. Maisch GmbH, Ammerbuch, Germany; 40 cm × 50 µm ID, packed in-house) at ∼100 nL/min in a 2 hour (non-labeled) or 3 hour (stable isotope dimethyl labeled) gradient from 0 to 40% solvent B (0.1 M acetic acid in 8/2 (v/v) acetonitrile/water). The eluent was sprayed via distal coated emitter tips (New Objective), butt-connected to the analytical column. The mass spectrometer was operated in data dependent mode, automatically switching between MS and MS/MS. Full scan MS spectra (from *m/z* 300–1500) were acquired in the Orbitrap with a resolution of 60,000 at *m/z* 400 after accumulation to target value of 500,000. The three most intense ions at a threshold above 5000 were selected for collision-induced fragmentation in the linear ion trap at normalized collision energy of 35% after accumulation to a target value of 10,000.

### Data analysis

All MS^2^ spectra were converted to single DTA files using Bioworks 3.3 at default settings. Runs were searched using an in-house licensed MASCOT search engine (Mascot version 2.1.0) software platform (Matrix Science, London, UK) against the Human IPI database version 3.36 (labeled sample; 63012 sequences) or version 3.37 (non-labeled sample; 69164 sequences) with carbamidomethyl cysteine as a fixed modification. Light, intermediate and heavy dimethylation of peptide N-termini and lysine residues (for labeled samples only), oxidized methionine and phosphorylation of tyrosine, serine and threonine were set as variable modifications. Trypsin was specified as the proteolytic enzyme and up to two missed cleavages were allowed. The mass tolerance of the precursor ion was set to 5 ppm and for fragment ions 0.6 Da. Peptides were assigned to the first protein hit reported by Mascot. The assignment of phosphorylation sites of identified phosphopeptides was performed by the PTM scoring algorithm implemented in MSQuant as described previously [31]. Individual MS/MS spectra from phosphopeptides were accepted for a Mascot score ≥20 [22]. The FDR at this score was estimated to be less than 3.5% (and less than 1% for tyrosine phosphorylated peptides only) by performing a concatenated decoy database search [22]. All identified phosphopeptides that were found to be differentially phosphorylated were manually validated as previously described[59]. The quality of the spectrum was judged based on a sufficient number of b- and/or y-ions –preferably those that contain the pY residue- to consolidate the peptide sequence. Furthermore, no dominant neutral loss of phosphoric acid should be observed when no additional serine or threonine phosphorylation was annotated to assure phosphorylation of a tyrosine residue.

### Quantification

Quantification of peptide triplets of which at least one has obtained a Mascot peptide score of 20 was performed using an in-house dimethyl-adapted version of MSQuant [60], as described previously [22], [23]. Briefly, peptide ratios were obtained by calculating the extracted ion chromatograms (XIC) of the “light”, “intermediate” and “heavy” forms of the peptide using the monoisotopic peaks only. The total XIC for each of the peptide forms was obtained by summing the XIC in consecutive MS cycles for the duration of their respective LC-MS peaks in the total ion chromatogram using FT-MS scans. This total XIC was then used to compute the peptide ratio. Quantified proteins were normalized against the Log_2_ of the median of all peptides quantified. StatQuant, an in-house developed program [61], was used for normalization, outlier detection and determination of standard deviation. Ratios of phosphotyrosine levels were normalized to the ratios of (non-specifically binding) non-phosphorylated peptides.

### Western Blotting

Cells were lysed in 1X Cell Lysis Buffer (Cell Signaling Technology, Beverly MA, USA) supplemented with 1 mM phenylmethylsulphonyl fluoride (PMSF). Protein concentrations were determined using the DC protein assay (Bio-Rad laboratories Inc., Hercules CA, USA). Twenty micrograms of each sample was mixed with Laemmli buffer and boiled for 5 min at 95°C. All samples were subjected to SDS-PAGE and electro-transferred onto PDVF membranes (0.2 µm, Bio-Rad). Membranes were probed using the corresponding primary antibodies at the indicated dilutions. After incubation with the primary antibodies, appropriate peroxidase-conjugated secondary antibodies (Dako, Denmark) or fluorescent secondary antibodies (LI-COR Biosciences) were used to detect the bound antibodies. Protein bands were visualized either using a chemiluminescence detection reagent ECL Plus (Amersham, GE Healthcare, UK) or LI-COR ODYSSY imaging system (LI-COR Biosciences, Nebraska). All antibodies used are shown in Table S5.

### Human phospho-receptor tyrosine kinase array

Analysis of protein expression using the human phospho-RTK antibody array (R&D Systems Inc., Minneapolis) was performed according to manufacturer's instructions. Briefly, capture and control antibodies were spotted in duplicates on nitrocellulose membranes and incubated overnight with 300 µg protein lysate. The membrane was then washed extensively with buffer provided and, further incubated with pan anti-phospho-tyrosine antibody conjugated to horseradish peroxidase (HRP). After incubation, arrays were washed and visualized using chemiluminescence ECL Plus (Amersham).

### Dataset Comparison

The hESC phosphoproteome was compared to other datasets by mapping known pY site locations obtained from Phospho.ELM (version 8.2) and Rikova *et. al*. [25]. Using the IPI human database, 1382 and 4117 unique pY sites were mapped out from phospho.ELM and Rikova *et. al*. datasets respectively. Overlap was determined by counting the number of identical sites between the datasets.

## Supporting Information

Figure S1 **Activation of FGF-signaling in hESC.** Activation profile of HES-3 cells post-FGF-2 induction, by Western Blotting. Cells were starved of FGF-2 for 5 days and 10 ng/ml of FGF-2 was added at the indicated time.
(TIF)

Figure S2 **Comparison of hESC cumulative tyrosine phosphorylated dataset.** Overlap of identified phosphotyrosine sites between our dataset (hESC), those deposited in Phospho.ELM (ver. 8.2), and those identified in Rikova et. al. [25]. Of the 735 phospho-peptides identified in FGF-2 stimulated hESCs, 374 (50.9%) peptides are unique to our dataset.
(TIF)

Figure S3 **Effect of Src Kinase inhibitor on undifferentiated hESC.** Cells were treated with increase concentrations of Src Kinase inhibitor (SU6656) for 6 PD. (A) Morphology of hESC after treatment with SU6656. Cells treated with 4 µM of SU6656 showed significant reduce in cell number when compared to control (untreated hESC) Scale bar  =  500 µm and 100 µm respectively (B) Cell count of SU6656 treated hESC. (C) Pluripotent marker expression of SU6656 treated hESC using quantitative real time PCR. Data were expressed as mean + SEM and results were from triplicate runs.
(TIF)

Table S1 **Summary of antibodies used.**
(PDF)

Table S2 **List of cumulative phosphopeptides detected from FGF-2 stimulated hESC.**
(PDF)

Table S3 **Motif assignment for phosphopeptides from the cumulative dataset.**
(PDF)

Table S4 **List of phosphopeptides from the quantitated dataset.**
(PDF)

Table S5 **List of phosphopeptides classified into 5 clusters using cluster analysis.**
(TIF)

Text S1 **Supplementary Materials and Methods.**
(DOC)
