# Supplementary figures and images for: Tyrosine Phosphorylation Profiling in FGF-2 Stimulated Human Embryonic Stem Cells
Source: PLoS One. 2011 Mar 17;6(3):e17538. doi: 10.1371/journal.pone.0017538 (PMC3060089; doi:10.1371/journal.pone.0017538)

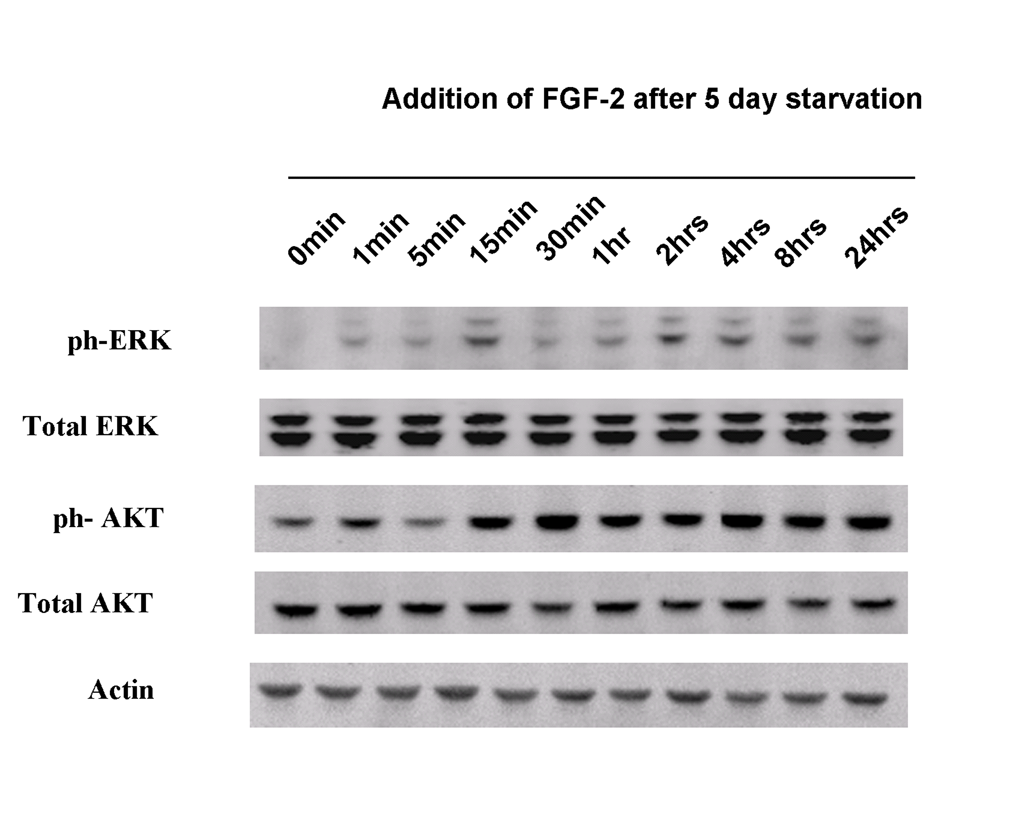

Supplement: Figure S1 — Activation of FGF-signaling in hESC. Activation profile of HES-3 cells post-FGF-2 induction, by Western Blotting. Cells were starved of FGF-2 for 5 days and 10 ng/ml of FGF-2 was added at the indicated time. (TIF) [file pone.0017538.s001.tif]

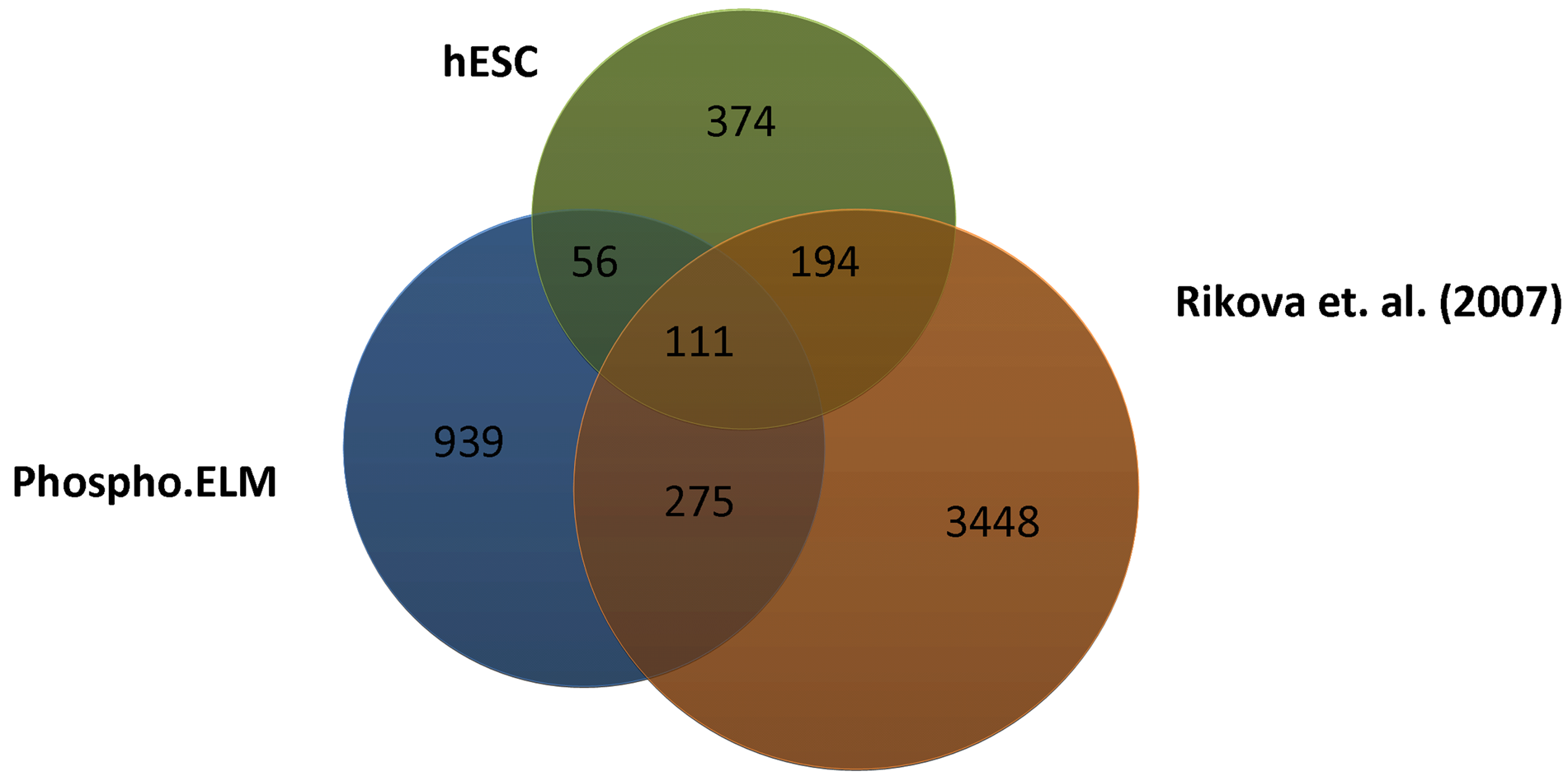

Supplement: Figure S2 — Comparison of hESC cumulative tyrosine phosphorylated dataset. Overlap of identified phosphotyrosine sites between our dataset (hESC), those deposited in Phospho.ELM (ver. 8.2), and those identified in Rikova et. al. [25]. Of the 735 phospho-peptides identified in FGF-2 stimulated hESCs, 374 (50.9%) peptides are unique to our dataset. (TIF) [file pone.0017538.s002.tif]

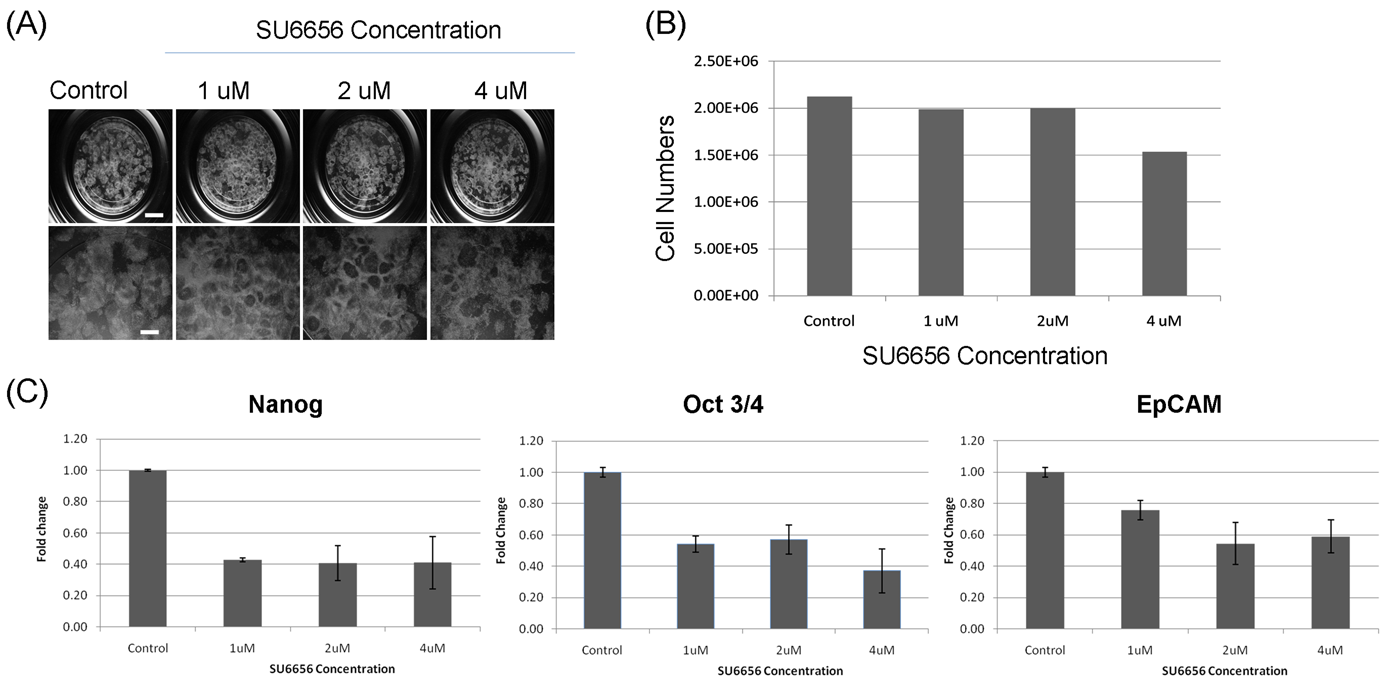

Supplement: Figure S3 — Effect of Src Kinase inhibitor on undifferentiated hESC. Cells were treated with increase concentrations of Src Kinase inhibitor (SU6656) for 6 PD. (A) Morphology of hESC after treatment with SU6656. Cells treated with 4 µM of SU6656 showed significant reduce in cell number when compared to control (untreated hESC) Scale bar = 500 µm and 100 µm respectively (B) Cell count of SU6656 treated hESC. (C) Pluripotent marker expression of SU6656 treated hESC using quantitative real time PCR. Data were expressed as mean + SEM and results were from triplicate runs. (TIF) [file pone.0017538.s003.tif]

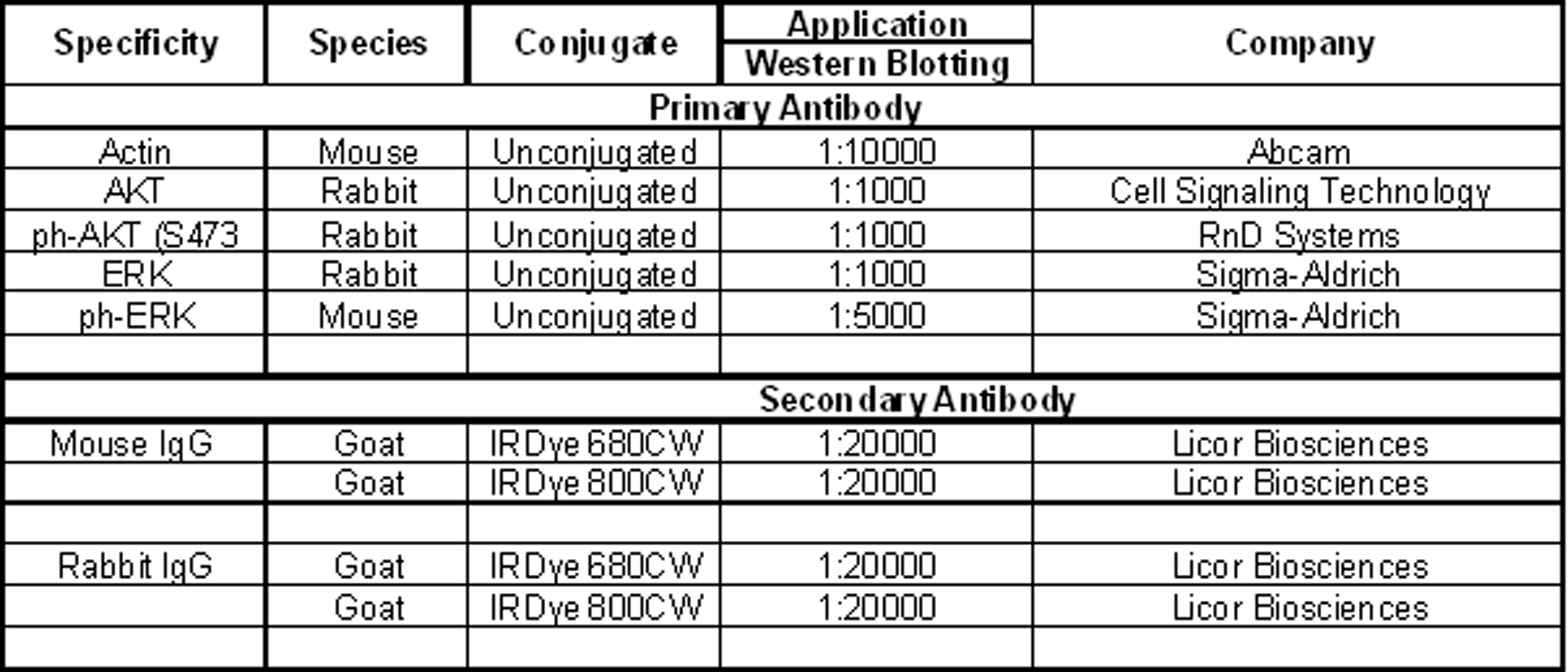

Supplement: Table S5 — List of phosphopeptides classified into 5 clusters using cluster analysis. (TIF) [file pone.0017538.s008.tif]
