# Supplementary material for: Tyrosine Phosphorylation Profiling in FGF-2 Stimulated Human Embryonic Stem Cells
Source: PLoS One. 2011 Mar 17;6(3):e17538. doi: 10.1371/journal.pone.0017538 (PMC3060089; doi:10.1371/journal.pone.0017538)
Supplement: Table S1 — Summary of antibodies used. (PDF) [file pone.0017538.s004.pdf]

IPI accession no.	Protein	pY site	Peptide	Mascot score	Dataset
IPI00000024	Isoform of protocadhedrin-1	863	DLpYAPKPSGK	31.9	5min_batch2
IPI00000070	Low-density lipoprotein receptor	828	NINSINFDNPVpYQK	74.5	15min_batch1
		845	TTEDEVHICHNQDGpYSYPSR	48.9	15min_batch1
IPI00000192	Isoform F of protein son	949	LGQDPpYRLGHDPYR	20.6	5min_batch2
IPI00000352	Isoform 1 of dual specificity tyrosine-phosphorylated regulated-kinase 1B	63	HINEVpYYAK	30.6	5min_batch1
		273	IYQpYIQSR	53.6	0min_batch1
IPI00000775	Isoform 1 of leucine-rich repeats and immunoglobin-like domains protein-1	941	VVCSDCNTEVDCpYSR	72.9	60min_batch1
IPI00000878	Tyrosine protein kinase tec	519	YVLDDQpYTSSSGAK	34.9	60min_batch1
IPI00001466	Echinoderm microtubles associated protein-like 4	226	DVIINQEGEpYIK	71.5	60min_batch1
IPI00001477	Isoform 1 of epithelia discoidin domain-containing receptor 1	484	EPPPpYQEPRPR	30.9	60min_batch2
		513	GNPPHSAPCVPNGSALLLSNPApYR	53.7	15min_batch1
		520	LLLATpYARPPR	20.9	60min_batch1
		792	NLpYAGDYYR	29.4	1min_batch1
		792	NLpYAGDYpYR	27.0	60min_batch2
		792	NLpYAGDpYYR	40.1	5min_batch1
		796	NLpYAGDpYYR	40.1	5min_batch1
		797	NLYAGDYpYR	20.3	5min_batch2
		797	NLpYAGDYpYR	27.0	60min_batch2
IPI00001654	Isoform 3 of pericentrolar maternal 1 protein	1004	NNCPFSADENpYRPLAK	28.9	1min_batch1
		1215	TEpY*MAFPKPFESSSSIGAEKPR	48.3	5min_batch2
IPI00001754	Junctional adhesion molecule A	280	KVIpYSQPSAR	55.1	60min_batch1
		280	KVIpYSQPpSAR	41.0	60min_batch1
		280	VIpYSQPSAR	48.7	60min_batch1
		280	VIpYSQPpSARSEGEFK	20.7	15min_batch1
		280	VIpYSQPSARpSEGEFK	34.9	5min_batch2
IPI00001762	Oligophrenin-1	370	LW*MEA*MDGKEPIpYHSPITK	28.6	5min_batch2
IPI00002211	Isoform 2 of semaphorin-6A	869	*MSEVAQMALEDQAATLEpYK	47.6	1min_batch1
		933	RLE*MHHSSSYGVDpYK	27.4	5min_batch2
		933	LE*MHHSSSYGVDpYK	39.9	5min_batch2
		1009	QPSLNApYNpSLTR	24.1	5min_batch2
		1009	QPSLNApYNSLTR	46.3	5min_batch2
IPI00002491	Isoform 9 of sorbin and SH3 domain-containing protein-1	291	LNRDDDSDLpYSPR	44.7	5min_batch2
IPI00002857	Isoform CSBP2 of mitogen-activated protein kinase 14	182	HTDDEMpTGpYVATR	57.0	5min_batch1
		182	HTDDE*MpTGpYVATR	51.2	5min_batch1
		182	HTDDE*MTGpYVATR	68.3	5min_batch2
		182	HTDDEMTGpYVATR	75.0	60min_batch1
IPI00002966	Heat shock 70kDa protein 4	336	EDIpYAVEIVGGATR	67.2	5min_batch2
IPI00003145	Isoform 1 of mitogen-activated protein kinase 8	185	TAGTSFM*MTPpYVVTR	51.8	60min_batch1
		185	TAGTSF*M*MTPpYVVTR	59.7	5min_batch1
		185	TAGTSF*MMpTPpYVVTR	41.3	1min_batch1
		185	TAGTSF*M*MpTPpYVVTR	40.1	5min_batch1
		185	TAGTSFMMTPpYVVTR	46.0	1min_batch1
		185	TAGTSF*MMTPpYVVTR	49.1	60min_batch1
IPI00003373	Occludin	287	SNILWDKEHIpYDEQPPNVEEWVK	35.5	60min_batch1
IPI00003377	Isoform 1 of splicing factor, argnine/serine rich-7	33	AFSpYYGPLR	39.9	1min_batch1
IPI00003431	Mitogen-activated protein kinase 6	467	ESEVNHpYYEPK	20.6	5min_batch2
		468	ESEVNHYpYEPK	25.5	5min_batch1
IPI00003479	Mitogen-activated protein kinase 1	187	VADPDHDHpTGFLTEpYVATR	39.8	15min_batch1
		187	VADPDHDHTGFLTEpYVATR	74.3	15min_batch2
		187	VADPDHDHTGFLpTEpYVATR	50.2	5min_batch2
IPI00003648	Isoform 1 of poliovirus receptor-related protein 1	468	YDEDAKRPpYFTVDEAEAR	70.0	15min_batch1
IPI00003843	cDNA FLJ53324, Highly similar to tight junction protein ZO-2	249	GRpSIDQDpYER	42.0	5min_batch2
		261	AYDPDpYERAYpSPEYR	39.8	15min_batch1
		265	AYDPDYERApYpSPEYR	37.0	1min_batch1
		423	RHQpYSDYDYHSSSEK	38.3	5min_batch1
		426	RHQYSDpYDYHSSSEK	44.1	5min_batch2
		426	HQYSDpYDYHSSSEK	45.9	5min_batch2
		426	RHQYSDpYDYHpSSSEK	26.6	15min_batch2
		506	TFLRPSPEDEAIpYGPNTK	47.3	60min_batch1
		1007	TQNKEESpYDFSK	53.9	60min_batch1
		1118	HPDIpYAVPIK	36.4	60min_batch1
		1118	IEIAQKHPDIpYAVPIK	40.7	15min_batch2
		1166	GSYGpSDAEEEEpYRQQLSEHSK	35.3	15min_batch1
IPI00003865	Isoform of heat shock cognate 71kDa protein	41	TTPSpYVAFTDTER	71.7	1min_batch2

IPI00003921	Isoform 1 of protein 4.1	660	LDGENIpYIR	29.6	60min_batch1
IPI00003985	Mitochondria chaperone BCS1	181	TV*MYTAVGSEWRPFGpYPR	62.4	1min_batch1
IPI00004279	Solute carrier family 15 member 1	697	SNPpYF*MSGANSQK	32.5	60min_batch2
IPI00004497	Isoform 1 of breakpoint cluster protein	177	KGHGQPGADAEKPFpYVNVEFHHER	31.6	15min_batch1
		177	GHGQPGADAEKPFpYVNVEFHHER	58.9	15min_batch1
IPI00004500	NEDD4-binding protein 3	83	NEPADpYATLYYR	51.3	1min_batch1
		87	NEPADYATLpYYR	60.7	1min_batch1
IPI00004901	cDNA FLJ20242 FIS clone COLF6369	98	VPSEGApYDIILPR	67.5	5min_batch2
		125	AED*MpYSAQSHQAATPPKDGK	33.8	5min_batch1
		125	AED*MpYSAQSHQAApTPPKDGK	26.0	5min_batch2
		125	AEDMpYSAQSHQAATPPKDGK	48.6	60min_batch1
		125	AED*MpYSAQSHQAATPPK	38.1	5min_batch2
IPI00005038	Ribonuclease UK114	110	AApYQVAALPK	29.9	5min_batch1
IPI00005126	Isoform 1 of Ephrin-A4	304	TADSVFCPHpYEK	56.6	5min_batch2
		330	VSGDYGHPVYIVQE*MPPQSPANIpYYK V	39.5	5min_batch2
IPI00005142	Isoform 1 of basic fibroblast growth factor recpetor 1	583	RPPGLEpYCpYNPSHNPEEQLSSK	33.2	1min_batch1
		583	RPPGLEpYCYNPSHNPEEQLSSK	51.3	15min_batch1
		585	RPPGLEYCpYNPpSHNPEEQLSSK	34.4	1min_batch1
		585	RPPGLEpYCpYNPSHNPEEQLSSK	33.2	1min_batch1
		585	RPPGLEYCpYNPSHNPEEQLSSK	48.2	15min_batch2
		653	DIHHIDpYpYK	32.8	60min_batch1
		653	DIHHIDpYpYKK	38.5	60min_batch1
		653	DIHHIDpYYKK	49.2	60min_batch2
		654	DIHHIDpYpYK	32.8	60min_batch1
		654	DIHHIDYpYKK	46.9	15min_batch2
		654	DIHHIDpYpYKK	38.5	60min_batch1
		654	DIHHIDYpYK	23.4	15min_batch2
IPI00005264	Isoform 2 of plakophilin-2	161	AHpYTHSDYQYSQR	44.1	60min_batch1
		166	AHYTHSDpYQYSQR	52.9	60min_batch1
		168	AHYTHSDYQpYSQR	39.3	15min_batch2
		631	YSQNIpYIQNR	26.8	5min_batch2
IPI00005416	Nice-4 protein (fragement)	354	DGSLASNPpYSGDLTK	35.9	60min_batch2
IPI00005741	Mitogen-activated protein kinase 13	182	HADAEMTGpYVVTR	66.6	60min_batch1
		182	HADAE*MTGpYVVTR	47.1	60min_batch2
		182	HADAE*MpTGpYVVTR	42.9	5min_batch1
IPI00005904	Probable ATP-dependent RNA helicase DDX20	756	LQTEAQEDDWpYDCHR	39.9	1min_batch1
IPI00006070	Isoform CD34-F of hematopoietic progention cell antigen CD34	286	KLGILDFTEQDVApSHQSpYSQK	21.9	0min_batch1
IPI00006176	Isoform 1 of hepatocyte growth factor-regulated tyrosine kinase substrate	132	VVQDTpYQI*MK	26.2	1min_batch2
		132	VVQDTpYQIMK	29.9	60min_batch1
		216	VCEPCpYEQLNR	57.6	15min_batch2
IPI00006196	Isoform 2 of nuclear mitotic apparatus protein 1	1822	KLDVEEPDSANSSFpYSTR	40.0	1min_batch1
IPI00006197	Isoform 1 of nuclear valosin-containing protein-like	655	GPELLN*MpYVGESER	79.2	1min_batch1
IPI00006482	Isoform long of sodium/potasssium-transporting ATPase subunit alpha-1	260	GIVVpYTGDR	57.5	60min_batch2
IPI00006608	Isoform APP770 of amyloid beta A4 protein (fragement)	757	*MQQNGpYENPTYKFFEQ*MQN	32.0	5min_batch1
		757	*MQQNGpYENPTYK	54.7	60min_batch2
		757	MQQNGpYENPTYK	65.0	60min_batch1
		762	*MQQNGYENPTpYKFFEQ*MQN	32.7	5min_batch2
		762	*MQQNGYENPTpYK	50.3	60min_batch1
		762	MQQNGYENPTpYK	43.3	1min_batch2
IPI00006658	Isoform 2 of peptidyl-prolyl cis-trans isomerase nima-interacting 4	147	FGpYHII*MVEGR	45.1	5min_batch2
		147	FGpYHIIMVEGR	47.0	15min_batch1
IPI00007067	Golgi-associated plant pathogenesis-related protein 1	42	EAQQpYSEALASTR	55.1	60min_batch2
IPI00007248	Pleckstrin homology domain-containing family A member 6	492	SEDIpYADPAAYVMR	54.4	1min_batch1
		492	SEDIpYADPAAYV*MR	102.6	15min_batch2
		492	LPPRSEDIpYADPAAYVMR	38.5	1min_batch1
		492	LPPRSEDIpYADPAAYV*MR	38.2	5min_batch2
IPI00007582	Sodium-and chloride-dependent creatine transporter 1	11	KSAENGIpYSVSGDEK	83.4	5min_batch2
		11	SAENGIpYSVSGDEK	67.5	1min_batch2
IPI00007750	Tubulin alpha-4A chain	399	FDL*MpYAK	34.4	5min_batch2
IPI00007935	PDZ and LIM domain protein 5	251	YTEFpYHVPTHSDASK	40.2	5min_batch2
IPI00008239	cDNA FLJ55176, highly similar to G-protein coupled receptor family C group 5 member B	376	SNVpYQPTEMAVVLNGGTIPTAPPSHTG R	75.7	1min_batch1
IPI00008290	Ephrin receptor	833	VLEDDPEAApYTTR	81.8	1min_batch2

IPI00008315	Ephrin type-B receptor 1	600	IYIDPFTpYEDPNEAVR	68.6	1min_batch2
		778	YLQDDTSDPTpYTSSLGGK	68.0	15min_batch1
IPI00008318	Ephrin type-A receptor 4	596	TpYVDPFTYEDPNQAVR	96.9	60min_batch1
		596	TpYVDPFTpYEDPNQAVR	95.5	5min_batch2
		602	TYVDPFTpYEDPNQAVR	92.0	1min_batch1
		602	pTYVDPFTpYEDPNQAVR	81.5	1min_batch1
		602	TpYVDPFTpYEDPNQAVR	95.5	5min_batch2
IPI00008438	40S ribosomal protien S10	12	IAIpYELLFK	62.5	15min_batch1
IPI00008530	60S acidic ribosomal protein P0	24	IIQLLDDpYPK	62.5	1min_batch2
IPI00008868	Microtubule-associated protein 1B	1062	AAEAGGAEEQpYGFLTTPTK	73.9	1min_batch2
		1796	ESSPLpYSPTFSDSTSAVK	51.8	15min_batch1
		1889	SPDEEDYDpYESYEK	44.3	1min_batch1
		1905	TSDVGGYpYYEK	25.6	60min_batch2
		1906	TSDVGGYYpYEK	40.7	5min_batch1
		1938	TPEDGDpYSYEIIEK	47.5	1min_batch1
		1940	TPEDGDYSpYEIIEK	58.7	1min_batch1
		2042	TPDTSTYCpYETAEK	43.8	5min_batch2
IPI00009236	Isoform 1 of caveolin-1	14	YVDSEGHLpYTVPIR	79.8	5min_batch2
IPI00009329	Utrophin	2599	Q*MPIGGDVPALQLQpYDHCK	39.5	5min_batch2
IPI00009342	Ras GTPase-activating-like protein IQGAP1	1510	LQQTpYAALNSK	51.1	5min_batch2
IPI00010204	Splicing factor, arginine/serine rich 3	32	AFGpYYGPLR	41.9	5min_batch2
IPI00010490	Isoform long of armadillo repeat protein deleted in velo-cardio-facial syndrome	201	DSPSpYGSLSR	42.6	60min_batch2
IPI00010680	Isoform 1 of fibroblast growth factor receptor 2	586	RPPG*MEpYSYDINRVPEEQ*MTFK	52.7	1min_batch1
		656	DINNIDpYpYKK	33.0	5min_batch1
		656	DINNIDpYYKK	36.6	0min_batch1
		657	DINNIDpYpYKK	33.0	5min_batch1
		657	DINNIDYpYKK	30.4	5min_batch2
IPI00010732	Parathyroid hormone/parathyroid hormone-related peptide receptor	494	SGSSSpYSpYGP*MVSHTSVTNVGPR	57.4	1min_batch1
		494	SGSSSpYSYGP*MVSHTSVTNVGPR	52.1	15min_batch1
		494	SGSSSpYSYGPMVSHTSVTNVGPR	83.8	1min_batch1
		496	SGSSSYSpYGP*MVSHTSVTNVGPR	58.2	1min_batch1
		496	SGSSSYSpYGPMVSHTSVTNVGPR	67.7	15min_batch1
		496	SGSSSpYSpYGP*MVSHTSVTNVGPR	57.4	1min_batch1
IPI00010740	Isoform long of splicing factor, proline-and glutamine rich	488	FAQHGTFEpYEYSQR	73.8	1min_batch1
IPI00011084	Claudin 6	214	GPSEpYPTKNpYV	31.7	5min_batch2
		219	GPSEpYPTKNpYV	31.7	5min_batch2
		219	GPSEYPTKNpYV	47.2	15min_batch2
IPI00011564	Syndecan-4	197	KAPTNEFpYA	25.8	60min_batch2
IPI00011652	Isoform EFS1 of embryonal FYN-associated substrate	148	DALEVpYDVPPTALR	65.2	1min_batch1
		163	VPSSGPpYDCPASFSHPLTR	76.2	1min_batch1
IPI00011676	Neural wiskott-aldrich syndrome protein	256	VIpYDFIEK	41.4	5min_batch1
		256	ETSKVIpYDFIEK	20.3	60min_batch1
IPI00011736	Phosphotidylinositol 3-kinase regulatory subunit beta	464	SREYDQLpYEEYTR	56.6	15min_batch1
		464	EYDQLpYEEYTR	51.8	60min_batch2
		467	EYDQLYEEpYTR	43.4	5min_batch2
		605	NETEDQpYALMEDEDDLPHHEER	121.3	15min_batch1
		605	NETEDQpYAL*MEDEDDLPHHEER	103.1	1min_batch1
IPI00011913	Heterogenous nuclear ribonucleoprotein A0	180	AVPKEDIpYSGGGGGGSR	49.9	5min_batch2
IPI00011998	Breast cancer anti-estrogen resistance 1	128	AQQGLpYQVPGPSPQFQSPPAK	62.9	15min_batch2
		234	VGQGYVYEAAQPEQDEpYDIPR	59.4	15min_batch1
		249	HLLAPGPQDIpYDVPPVR	57.6	5min_batch2
		267	GLLPSQYGQEVpYDTPP*MAVK	50.7	1min_batch1
		287	GPNGRDPLLEVpYDVPPSVEK	44.5	1min_batch1
		287	DPLLEVpYDVPPSVEK	27.0	1min_batch1
		345	EETpYDVPPAFAK	29.7	5min_batch2
		428	VLPPEVADGGVVDSGVpYAVPPPAER	43.5	1min_batch1
IPI00012007	Adenosylhomocysteinase	193	SKFDNLpYGCR	53.4	5min_batch2
IPI00012011	Cofilin-1	140	HELQANCpYEEVKDR	56.3	15min_batch2
IPI00012752	Isoform 3 of growth factor receptor-bound protein 10	404	YG*MLLpYQNYR	37.4	5min_batch2
		404	YGMLLpYQNYR	23.5	1min_batch1
IPI00012885	Isoform 1 of focal adhesion kinase	397	THAVSVSETDDpYAEIIDEEDTYT*MPSTR	66.8	1min_batch1
		397	THAVSVSETDDpYAEIIDEEDTYTMPSTR	63.8	5min_batch2
		397	THAVSVSETDDpYAEIIDEEDTpYT*MPSTR	38.2	5min_batch2
		407	THAVSVSETDDpYAEIIDEEDTpYT*MPSTR	38.2	5min_batch2

		441	CIGEGQFGDVHQGIpY*MSPENPALAVAIK	50.3	0min_batch1
		570	pY*MEDSTpYYK	32.7	5min_batch1
		576	Y*MEDSTpYYK	42.2	0min_batch1
		576	Y*MEDpSTpYYKASK	34.6	5min_batch2
		576	YMEDSTpYYKASK	54.6	1min_batch2
		576	Y*MEDSTpYpYK	39.7	5min_batch1
		576	YMEDSTpYpYKASK	52.8	5min_batch2
		576	Y*MEDSTpYYKASK	56.8	60min_batch1
		576	Y*MEDSpTpYYKASK	40.3	5min_batch2
		576	YMEDSTpYpYK	37.8	15min_batch1
		576	pY*MEDSTpYYK	32.7	5min_batch1
		576	Y*MEDSTpYpYKASK	51.7	15min_batch2
		576	YMEDSpTpYYKASK	37.5	60min_batch1
		576	YMEDSTpYYK	51.3	15min_batch1
		576	Y*MEDpSTpYYK	20.4	5min_batch2
		577	YMEDSTYpYKApSK	23.1	5min_batch2
		577	Y*MEDSTYpYK	39.2	60min_batch1
		577	Y*MEDSTpYpYKASK	51.7	15min_batch2
		577	Y*MEDSTpYpYK	39.7	5min_batch1
		577	YMEDSTpYpYKASK	52.8	5min_batch2
		577	YMEDSTpYpYK	37.8	15min_batch1
		577	YMEDSTYpYK	54.8	60min_batch1
		861	GSIDREDGSLQGPIGNQHIpYQPVGKPDPAAPPK	44.7	1min_batch1
IPI00013068	Eukaryotic translation initiation factor 3 subunit E	445	SEAPNWATQDSGFpY	70.4	1min_batch1
IPI00013070	Isoform 1 of heterogenous nuclear ribonucleoprotein U-like protien 1	510	NYILDQTNVpYGSAQR	59.1	5min_batch2
IPI00013174	Isoform 1 of RNA binding protein 14	226	ASpYVAPLTAQPATYR	35.0	1min_batch1
IPI00013396	U1 small nuclear ribonucleoprotein C	8	FYCDpYCDTYLTHDSPSVR	28.2	1min_batch1
IPI00013721	Serine/Threonine-protein kinase PRP4 homolog	849	LCDFGSASHVADNDITPpYLVSR	79.8	1min_batch1
IPI00013877	Isoform 1 of heterogenous nuclear ribonucleoprotein H13	296	DGMDNQGGpYGSVGR	59.6	60min_batch1
IPI00013881	Heterogenous nuclear ribonucleoprotein H	266	DLNpYCFSG*MSDHR	54.3	5min_batch2
		266	DLNpYCFSGMSDHR	20.2	1min_batch1
IPI00013894	Stress-induced phosphoprotein 1	354	LApYINPDLALEEK	23.2	1min_batch1
IPI00013933	Isoform DPI of desmoplakin	28	AESGPDLRpYEVTSGGGGTSR	58.1	1min_batch1
		56	RGVITDQNSDGpYCQTGT*MSR	49.7	5min_batch2
		172	GGGGpYTCQSGSGWDEFTK	82.3	5min_batch2
IPI00013981	Proto-oncogene tyrosine-protien kinase YES	222	KLDNGGpYYITTR	79.7	60min_batch1
		223	KLDNGGYpYITTR	49.8	0min_batch1
		426	LIEDNEpYTAR	61.6	5min_batch1
		446	WTAPEAALpYGR	28.2	5min_batch2
IPI00013983	Proto-oncogene tyrosine-protien kinase receptor RET	687	RPAQAFPVSpYSSSGAR	75.6	5min_batch2
		900	DVpYEEDSpYVKR	36.6	5min_batch2
		900	DVpYEEDpSYVKR	36.9	60min_batch2
		905	DVpYEEDSpYVKR	36.6	5min_batch2
		905	DVYEEDSpYVKR	27.8	5min_batch2
IPI00013988	Isoform 2 of RHO GTPase-activating protein 5	1108	GYSDEIpYVVPDDSQNR	72.6	5min_batch2
IPI00014172	Lysosomal-associated transmembance protein 4A	230	MPEKEPPPPpYLPA	69.6	1min_batch1
		230	*MPEKEPPPPpYLPA	48.1	5min_batch2
IPI00014177	Isoform 1 of septin-2	17	QQPTQFINPETPGpYVGFANLPNQVHR	66.9	15min_batch2
IPI00014197	Isoform 1 of protein CDV3 homolog	190	KTPQGPPEIpYSDTQFPSLQSTAK	60.7	60min_batch1
		244	LQLDNQpYAVLENQK	66.0	15min_batch1
IPI00014344	Isoform long of dual specificity tyrosine-phosphorylation-regulated kinase 1A	145	VYNDGYDDDNpYDYIVK	115.4	15min_batch1
		145	KVYNDGYDDDNpYDYIVK	77.6	15min_batch2
		147	VYNDGYDDDNYDpYIVK	68.4	1min_batch2
IPI00014424	Elongation factor 1-alpha 2	29	STTTGHLIpYK	63.5	5min_batch2
		141	EHALLApYTLGVK	51.2	0min_batch1
IPI00014454	Isoform RIN1 of Ras and Rab interactor 1	36	EKPAQDPLpYDVPNASGGQAGGPQRPGR	83.2	15min_batch1
IPI00014873	cDNA FLJ59580, highly similar to cell division protein kinase 10	50	IGEGTpYGIVYRAR	27.0	15min_batch1
IPI00014898	Isoform of plectin-1	4615	GYYSPpYSVSGSGSTAGSR	115.7	15min_batch1
IPI00015180	Protein shroom 2	210	DSApYGSFSTSSSTPDHTLSK	43.5	1min_batch1
		1257	TLSTSEQFpYSR	52.4	5min_batch1
IPI00015287	Isoform 1 of docking protien 1	409	VKEEGYELPYNPATDDpYAVPPPR	26.0	15min_batch1
		449	SHNSALpYSQVQK	79.8	60min_batch2
IPI00015580	Isoform 3 of formin-binding protein 1-like	291	SGFEPPGDFPFEDYSQHIpYR	34.9	1min_batch1

IPI00015756	Isoform 1 of receptor-type tyrosine-protein phosphatase KAPPA	858	YLCEGTESPpYQTGQLHPAIR	35.2	5min_batch2
IPI00016371	Isoform JM-A of receptor tyrosine-protein kinase ERBB-4	733	VLGSGAFGTVpYK	24.5	15min_batch1
IPI00016373	Ras-related protein Rab-13	5	ApYDHLFK	54.5	5min_batch2
IPI00016645	Isoform 1 of ephrin type-A receptor 7	597	ADQEGDEELpYFHFK	59.7	1min_batch2
		608	TpYIDPETYEDPNR	72.9	15min_batch1
		614	TYIDPETpYEDPNR	48.8	5min_batch1
		791	VIEDDPEAVpYTTTGGK	79.5	15min_batch1
		791	VIEDDPEAVpYTTTGGKIPVR	47.5	15min_batch1
IPI00016670	RHOA activator C11ORF59	40	ALNGAEPNpYHSLPSAR	44.1	1min_batch1
IPI00016736	1-phosphotidylinositol-4,5-biphosphate phosphodiesterase gamma-1	472	LAEGSApYEEVPTS*M*MYSENDISNSIK	37.5	5min_batch2
		771	IGTAEPDpYGALYEGR	67.5	5min_batch1
		775	IGTAEPDYGALpYEGR	39.5	5min_batch2
		783	NPGFpYVEANP*MPTFK	75.2	0min_batch1
		783	NPGFpYVEANPMPTFK	61.0	1min_batch1
		977	ACpYRDMSSFPETK	46.7	5min_batch2
		977	ACpYRD*MSSFPETK	40.8	60min_batch1
IPI00016910	Eukaryotic translation initiation factor 3 subunit C	881	QGTpYGGYFR	20.5	5min_batch2
		884	QGTYGGpYFR	32.4	5min_batch2
IPI00016932	Isoform 1 of phosphotidylinositol-3,4,5-trisphosphate 5-phosphatase 2	886	LpYEWISIDKDEAGAK	80.6	15min_batch1
		986	NSFNNPApYYVLEGVPHQLLPPEPPSPAR	65.8	15min_batch2
		1135	TLSEVDpYAPAGPAR	84.1	60min_batch2
IPI00017292	Isoform 1 of catenin beta-1	489	LHpYGLPVVVK	32.1	60min_batch1
IPI00017562	Isoform 2 of latrophilin-2	1350	RSENEDIpYYK	60.4	5min_batch1
IPI00017578	SH2 domain-containing adaptor protein B	201	LDpYCGGSGEPGGVQR	84.7	1min_batch2
		333	VTIADDpYSDPFDAK	73.4	15min_batch1
		333	DKVTIADDpYSDPFDAK	75.8	15min_batch1
		333	VTIADDpYSDPFDAKNDLK	43.1	1min_batch2
		355	GESAGpY*MEPYEAQR	88.6	60min_batch1
		355	AGKGESAGpYMEPYEAQR	96.6	5min_batch2
		355	AGKGESAGpY*MEPYEAQR	78.3	5min_batch2
		355	GESAGpYMEPYEAQR	81.4	5min_batch2
		355	AGKGESAGpY*MEPpYEAQR	31.4	5min_batch2
		359	AGKGESAGpY*MEPpYEAQR	31.4	5min_batch2
		384	GIQLpYDTPYEPEGQSVDpSDSESTVSPR	49.3	15min_batch2
		384	GIQLpYDTPYEPEGQSVDSDSESTVSPR	63.2	1min_batch1
		384	GIQLpYDTPYEPEGQSVDSDpSESTVSPR	47.9	1min_batch1
		423	LPQDDDRPADEpYDQPWEWNR	51.6	1min_batch1
IPI00018195	Mitogen-activated protein kinase 3	204	IADPEHDHTGFLpTEpYVATR	60.5	5min_batch2
		204	IADPEHDHTGFLTEpYVATR	80.5	0min_batch1
IPI00018274	Isoform 1 of epidermal growth factor receptor	1172	GSHQISLDNPDpYQQDFFPK	22.6	1min_batch1
IPI00018335	Isoform FLT1 of vascular endothelia growth factor receptor 1	1048	DIpYKNPDpYVR	41.0	5min_batch2
		1053	DIpYKNPDpYVR	41.0	5min_batch2
IPI00018434	Isoform 1 of tumor susceptibility gene 101 protein	390	KTAGLSDLpY	28.1	5min_batch2
IPI00018522	HMT1 HNRNP methyltransferase-like 2 isoform 1	309	TGFSTSPESPpYTHWK	29.9	1min_batch1
IPI00018534	Histone H2B type 1-L	43	KESYSVYVpYK	52.5	5min_batch2
		43	ESYSVYVpYK	25.6	1min_batch1
IPI00019025	Partitioning defective 6 homolog beta	101	KEEADpYSAFGTDTLIK	76.5	1min_batch2
IPI00019146	Isoform 1 of coxsackievirus and adenovirus receptor	313	SYIGSNHSSLGS*MSPSN*MEGpYSK	63.0	5min_batch1
		313	SYIGSNHSSLGSMSPSN*MEGpYSK	43.8	1min_batch1
		318	TQpYNQVPSEDFER	57.5	15min_batch1
IPI00019471	Insulin receptor substrate 1	465	GEEELSNpYIC*MGGK	66.5	5min_batch2
		612	GGHHRPDSSTLHTDDGpY*MP*MSPGVAPVPSGR	35.9	1min_batch1
		662	VDPNGpYMM*MSPSGGCSPDIGGGPSSSSSSNAVPSGTSYGK	35.8	15min_batch2
		662	VDPNGpYM*M*MSPSGGCSPDIGGGPSSSSSSNAVPSGTSYGK	23.9	1min_batch1
		662	VDPNGpY*M*M*MSPSGGCSPDIGGGPSSSSSSNAVPSGTSYGK	46.4	1min_batch1
		941	CPSQLQPAPREEETGTEEpY*MK	30.7	5min_batch2
		941	CPSQLQPAPREEETGTEEpYMK	27.9	1min_batch1
IPI00019502	Isoform 1 of myosin-9	754	ALELDSNLpYR	49.2	15min_batch1

		1408	VAApYDKLEK	24.4	5min_batch1
IPI00020096	Isoform 1 of kinesin light chain 1	449	DGTSFGEpYGGWYK	41.0	1min_batch1
IPI00020178	Isoform 1 of signal tranducing adpator molecule 1	384	L*MNEDP*MYS*MpYAK	28.1	5min_batch1
IPI00020464	Phosphoprotein associated with glycoshingolipid-enriched microdomains 1	359	SPSSCNDLpYATVK	43.5	60min_batch2
IPI00020501	Myosin-11	761	ALELDPNLpYR	29.4	60min_batch1
IPI00020729	Insulin receptor substrate 4	743	GpY*M*M*MFPR	36.2	5min_batch2
IPI00021076	Isoform 1 of plakophilin-4	157	SSTQMNSYSDSGpYQEAGSFHNSQNVSK	70.3	1min_batch1
		306	QTSNPNGPTPQpYQTTAR	45.5	5min_batch2
		369	TVHD*MEQFGQQQpYDIYER	63.7	1min_batch1
		369	TVHDMEQFGQQQpYDIYER	50.9	1min_batch1
		372	TVHD*MEQFGQQQYDIpYER	44.8	0min_batch1
		372	TVHDMEQFGQQQYDIpYER	70.2	15min_batch1
		415	SAVSPDLHITPIpYEGR	62.4	5min_batch2
		415	SAVpSPDLHITPIpYEGR	48.5	15min_batch2
		420	TpYYSPVYR	39.5	5min_batch1
		421	TYpYSPVYR	42.6	5min_batch2
		443	SPNHGTVELQGSQTALpYR	48.7	1min_batch1
		470	NNpYALNTTATpYAEPYRPIQYR	55.3	5min_batch2
		470	NNpYALNTTATYAEPYRPIQYR	92.4	1min_batch1
		478	NNYALNTTATpYAEPYRPIQYR	82.8	5min_batch1
		470	NNpYALNTTATpYAEPYRPIQYR	55.3	5min_batch2
		487	NNYALNTTATYAEPYRPIQpYR	23.5	15min_batch1
		1115	LQHQQLpYYSQDDSNRK	40.1	60min_batch1
		1116	LQHQQLYpYSQDDSNRK	34.1	5min_batch2
		1139	LYLQSPHSpYEDPYFDDR	54.9	15min_batch1
		1143	LYLQSPHSYEDPpYFDDR	52.1	1min_batch1
		1156	VHFPASTDpYSTQYGLK	83.7	5min_batch2
		1168	STTNpYVDFYSTK	70.5	1min_batch2
		1172	STTNYVDFpYSTK	64.1	5min_batch2
IPI00021267	Ephrin type-A receptor 2	575	QSPEDVpYFSK	32.0	5min_batch1
		588	SEQLKPLKTpYVDPHTYEDPNQAVLK	38.0	60min_batch1
		588	TpYVDPHpTYEDPNQAVLK	44.9	5min_batch2
		588	SEQLKPLKTpYVDPHTpYEDPNQAVLK	38.3	1min_batch1
		588	SEQLKPLKTpYVDPHpTYEDPNQAVLK	33.0	15min_batch1
		588	TpYVDPHTYEDPNQAVLK	45.1	1min_batch1
		588	TpYVDPHTpYEDPNQAVLK	62.5	1min_batch1
		594	SEQLKPLKTpYVDPHTpYEDPNQAVLK	38.3	1min_batch1
		594	SEQLKPLKpTYVDPHTpYEDPNQAVLK	33.5	1min_batch1
		594	TYVDPHTpYEDPNQAVLK	39.5	5min_batch1
		594	pTYVDPHTpYEDPNQAVLK	57.5	5min_batch2
		594	TpYVDPHTpYEDPNQAVLK	62.5	1min_batch1
		772	VLEDDPEATpYTTSGGKIPIR	89.8	15min_batch2
		772	VLEDDPEATpYTTSGGK	111.3	15min_batch2
IPI00021275	Isoform 1 of ephrin type-B receptor 2	780	FLEDDTSDPTpYTSALGGK	116.4	15min_batch1
IPI00021290	ATP-citrate synthase	682	TTDGVpYEGVAIGGDR	60.8	5min_batch2
IPI00021326	SHC transforming protein 1 isoform 3	428	ELFDDPSpYVNVQNLDK	90.2	1min_batch2
IPI00021396	Vascular endothelia growth factor receptor 2	801	TGpYLSIV*MDPDELPLDEHCER	80.0	5min_batch2
		1214	FHpYDNTAGISQYLQNSK	108.2	60min_batch1
IPI00021428	Actin, alpha skeletal muscle	93	IWHHTFpYNELR	35.2	1min_batch2
IPI00021439	Actin, cytoplasmic 1	166	TTGIV*MDSGDGVTHTVPIpYEGYALPHAILR	34.8	0min_batch1
		198	GpYSFTTTAER	59.6	60min_batch2
		294	KDLpYANTVLSGGTT*MYPGIADR	41.3	5min_batch2
IPI00021695	Isoform D of plasma membrane calcium-transporting ATPase 1	53	IQESpYGDVYGICTK	44.6	1min_batch1
		57	KIQESYGDVpYGICTK	33.2	1min_batch1
IPI00021917	Isoform 1 of receptor-interacting serine/threonine-protein kinase 2	381	KAQDCpYFMK	28.8	5min_batch2
IPI00021941	Isoform 1 of disks large-associated protein 1	118	DGpYHTLQYK	40.0	60min_batch2
IPI00022228	Vigilin	437	MDpYVEINIDHK	43.0	1min_batch1
IPI00022296	Isoform 1 of Mast/stem cell growth factor receptor	703	QEDHAEAALpYKNLLHSK	35.2	1min_batch1
		703	QEDHAEAALpYK	37.8	5min_batch1
		936	QISESTNHIpYSNLANCSPNR	27.3	15min_batch1
		936	QISESTNHIpYSNLANCpSPNR	36.3	15min_batch1
IPI00022353	Non-receptor tyrosine-protein kinase TYK2	292	LLAQAEGEPCpYIR	40.4	1min_batch1
IPI00022434	Putative uncharacterized protein ALB	164	YLpYEIAR	29.5	5min_batch1
IPI00022462	Transferrin receptor protein 1	20	SAFSNLFGGEPLSpYTR	93.3	15min_batch1

IPI00022521	Isoform 2 of dual specificity tyrosine-phosphorylation-regulated kinase 2	309	VYTpYIQSR	58.8	5min_batch1
IPI00022558	Isoform 1 of myelin protein zero-like protein 1	241	SLPSGSHQGPVIpYAQLDHSGGHHSDK	46.6	15min_batch1
		263	INKSESVVpYADIR	65.0	5min_batch2
		263	SESVVpYADIR	62.9	60min_batch2
		263	SESVVpYADIRK	42.9	60min_batch2
IPI00022661	Isoform delta od poliovirus receptor-related protein 2	513	INPIpYDALSYSSPSDSYQGK	83.9	1min_batch1
IPI00022959	Isoform 1 of poliovirus receptor-related protein 3	511	FERP*MDYpYEDLK	28.1	1min_batch1
		511	FERPMDYpYEDLK	25.9	15min_batch1
IPI00023343	Isoform 1 of disks large homolog 3	673	DNEVDGQDpYHFVVSR	42.5	60min_batch1
		511	RDNEVDGQDpYHFVVSR	51.0	1min_batch1
IPI00023461	Isoform 3 of afadin	94	YSLpYEVHVSGER	83.2	15min_batch2
IPI00023503	Cell division protein kinase 3	43	IGEGTpYGVVYK	59.5	0min_batch1
		43	IGEGpTpYGVVYK	75.2	5min_batch2
		43	IGEGTpYGVVpYK	63.8	5min_batch2
		47	IGEGTYGVVpYK	54.9	1min_batch1
		47	IGEGTpYGVVpYK	63.8	5min_batch2
		47	IGEGpTYGVVpYK	39.9	0min_batch1
IPI00023530	Cell division protein kinase 5	15	IGEGTpYGTVFK	53.5	15min_batch2
IPI00023704	Lipoma-preferred partner	244	SAQPSPHYMAAPSSGQIpYGSGPQGYNTQPVPVSGQCPPPSTR	56.6	15min_batch1
		273	GG*MDpYAYIPPPGLQPEPGYGYAPNQGR	42.3	1min_batch1
		297	YpYEGYYAAGPGYGGR	57.9	1min_batch1
		300	YYEGpYYAAGPGYGGR	51.8	5min_batch2
		301	YYEGYpYAAGPGYGGR	83.1	1min_batch1
		317	NDSDPTpYGQQGHPNTWK	54.3	5min_batch1
IPI00023785	DEAD box polypeptide 17 isoform 4	580	TTSSANNPNL*MpYQDECDRR	32.0	5min_batch2
IPI00023942	Syndecan 3 (SYND3). Isoform 2	383	QASVTYQKPDKQEEFpYA	30.5	1min_batch1
IPI00023974	Pituitary timour-transforming gene 1 protein interacting protein	174	YGLFKEENPpYAR	31.5	15min_batch1
IPI00024067	Isoform 1 of clathrin heavy chain 1	634	ALEHFTDLpYDIKR	58.4	5min_batch2
IPI00024087	Pyruvate dehydrogenase E1 component subunit alpha, testis-specific form, mitochondria	287	pYHGHS*MSDPGVSpYR	31.2	15min_batch2
		299	YHGHSMSDPGVSpYR	42.5	60min_batch1
		299	YHGHpS*MSDPGVSpYR	30.3	60min_batch1
		299	YHGHS*MSDPGVSpYR	35.6	60min_batch1
		299	pYHGHS*MSDPGVSpYR	31.2	15min_batch2
IPI00024097	Isoform 1 of testin	251	EGDPAIpYAER	21.0	5min_batch2
IPI00024282	Ras-related protein Rab-8B	5	TpYDYLFK	44.9	60min_batch1
IPI00024307	Ephrin-B1	313	TTENNpYCPHYEK	36.3	5min_batch2
		317	TTENNYCPHpYEK	50.3	60min_batch2
IPI00024312	FYVE, RHOGEF and PH domain containing protein 1	621	LLpYCVPR	37.1	5min_batch2
IPI00024320	Putative RNA-binding protein 3	127	YYDSRPGGYGpYGYGR	21.3	15min_batch1
		129	YYDSRPGGYGYGpYGR	42.7	1min_batch1
IPI00024382	cDNA FLJ61103, highly similar to clip-associating protein 2	1234	DYNPpYNYSDSISPFNK	44.3	1min_batch1
IPI00024673	Isoform alpha-2 of mitogen-activated protein kinase 9	185	TACTNF*M*MTPpYVVTR	86.9	1min_batch1
		185	TACTNF*MMTPpYVVTR	47.0	5min_batch2
		185	TACTNFM*MTPpYVVTR	68.3	60min_batch1
		185	TACTNFMMTPpYVVTR	56.6	1min_batch1
IPI00024911	Endoplasmic recticulum protein ERP29	66	FDTQYPpYGEKQDEFK	39.1	1min_batch1
IPI00025803	Insulin receptor precursor	1205	DIpYETDpYYRK	40.2	5min_batch2
		1205	DIpYETDYYR	54.8	15min_batch1
		1205	DIpYETDYpYR	33.5	1min_batch2
		1205	DIpYETDpYYR	30.2	5min_batch1
		1205	DIpYETDYYRK	50.4	1min_batch1
		1205	DIpYETDYpYRK	29.3	5min_batch2
		1205	DIpYEpTDYYR	29.3	60min_batch2
		1205	DIpYETDpYpYRK	44.9	5min_batch2
		1209	DIYEpTDpYpYRK	21.9	5min_batch2
		1209	DIpYETDpYYRK	40.2	5min_batch2
		1209	DIpYETDpYpYRK	44.9	5min_batch2
		1209	DIYETDpYYR	39.5	1min_batch2
		1209	DIpYETDpYYR	30.2	5min_batch1
		1209	DIYEpTDpYYRK	36.9	5min_batch1
		1209	DIYETDpYYRK	29.7	5min_batch1
		1210	DIpYETDpYpYRK	44.9	5min_batch2
		1210	DIpYETDYpYR	33.5	1min_batch2
		1210	DIYEpTDpYpYRK	21.9	5min_batch2

		1210	DIYETDYpYR	30.5	60min_batch1
		1210	DIpYETDYpYRK	29.3	5min_batch2
		1210	DIYETDYpYRK	33.5	60min_batch2
		1375	SpYEEHIPpYTH*MNGGK	34.6	5min_batch2
		1381	SpYEEHIPpYTH*MNGGK	34.6	5min_batch2
IPI00025830	WEE1-like protein kinase	132	SPAAPpYFLGSSFSPVR	68.2	5min_batch2
IPI00025846	Isoform 2A of desmocollin-2	823	YTpYSEWHSFTQPR	70.4	15min_batch1
		853	HAQDpYVLTYNYEGR	46.1	1min_batch1
IPI00026689	Putative uncharacterized protein DKFZP686L20222	15	IGEGpTpYGVVYKGR	56.9	15min_batch1
		15	IGEGTpYGVVYKGR	48.1	1min_batch1
		15	IEKIGEGTpYGVVYK	29.4	5min_batch2
		15	IEKIGEGpTpYGVVYK	42.9	5min_batch2
		19	IGEGTYGVVpYKGR	78.6	60min_batch1
IPI00026889	Isoform DAB555 of disabled homolog 1	232	EGVpYDVPK	22.0	15min_batch2
IPI00026904	cDNA FLJ59367, highly similar to adenylosuccinate lyase	294	QQIGSSA*MPpYK	23.6	5min_batch2
IPI00026952	Plakophilin-3	84	GQpYHTLQAGFSSR	73.2	5min_batch2
		176	ADpYDTLSLR	24.8	60min_batch2
IPI00027174	Isoform 1 of fibroblast growth factor receptor 3	647	DVHNLDpYpYKK	45.3	5min_batch1
		647	DVHNLDpYYKK	36.8	1min_batch2
		648	DVHNLDpYpYKK	45.3	5min_batch1
		648	DVHNLDYpYKK	22.8	5min_batch2
IPI00027232	Insulin-like growth factor 1 receptor	1346	ASFDERQPpYAH*MNGGR	29.8	5min_batch2
IPI00027252	Prohibitin-2	121	VLSRPNAQELPS*MpYQR	28.4	1min_batch1
IPI00027269	E3 ubiquitin-protein ligase CBL	674	IKPSSSANAIpYSLAARPLPVPK	31.4	15min_batch1
IPI00027422	Isoform beta-4C of intergrin beta-4	1207	VCAYGAQGEGPpYSSLVSCR	57.0	5min_batch2
IPI00027438	Flotillin-1	203	VSAQpYLSEIEMAK	57.3	15min_batch1
IPI00027667	Isoform 1 of gastrin/cholecytokinin type B receptor	294	CRPETGAVGEDSDGCpYVQLPR	48.8	1min_batch1
IPI00027705	Isoform 1 of DNA primase large subunit	381	IILSNPPSQGDpYHGCPFR	29.8	1min_batch1
IPI00027721	Isoform 1 of alpha-type platelet-derived growth factor receptor	762	SLpYDRPASpYK	38.5	1min_batch2
		768	SLpYDRPASpYK	38.5	1min_batch2
IPI00027834	Heterogenous nuclear ribonucleoprotein L	92	TDNAGDQHGGGGGGGGGAGAAGGGGGGENpYDDPHKTPASPVVHIR	30.7	1min_batch1
IPI00028065	Cytoplasmic protein NCK1	105	LpYDLN*MPAYVK	48.2	15min_batch2
			LpYDLNMPAYVK	51.2	60min_batch1
IPI00028570	Isoform 1 of glycogen synthase kinase-3 beta	216	GEPNVSpYICSR	64.6	5min_batch2
			GEPNVSpYICpSR	54.3	5min_batch1
IPI00028931	Desmoglein-2	968	VpYAPASTLVDQPYANEGTVVVTER	44.6	1min_batch1
		979	VYAPASTLVDQPpYANEGTVVVTER	54.1	1min_batch1
		1013	VIQPHGGGSNPLEGTQHLQDVPpYVMVR	27.5	1min_batch2
IPI00029111	Collapsin response mediator protein long variant	545	NHQSAAEpYNIFEG*MELR	36.7	5min_batch2
IPI00029263	Proto-oncogene tyrosine-protein kinase FER	402	VQENDGKEPPPVVNpYEEDAR	78.4	5min_batch2
		714	QEDGGVpYSSSGLK	66.9	60min_batch2
IPI00029273	Isoform 1 of hepatocyte growth factor receptor	1003	SVSPTTEMVSNESVDpYR	80.4	1min_batch1
		1234	D*MYDKEpYpYSVHNK	27.9	5min_batch2
		1234	D*MYDKEpYYSVHNK	46.7	5min_batch2
		1234	DMYDKEpYYSVHNK	34.1	60min_batch1
		1235	D*MYDKEpYpYSVHNK	27.9	5min_batch2
IPI00029422	Kinesin-line protein KIF20A	869	TPTCQSSTDCSPpYAR	69.2	60min_batch1
IPI00029449	Vang-lilke protein 2	308	VpYSLGEENSTNNSTGQSR	99.5	60min_batch1
		342	DNSHNEYpYYEEAEHER	42.7	1min_batch1
IPI00029515	Isoform 1 of pleckstrin homology domain-containing family A member 5	134	ERPIS*MINEASNYNVTSDpYAVHP*MSPVGR	51.3	5min_batch2
		398	GGNRPNTGPLpYTEADR	58.0	5min_batch2
IPI00029601	Src substrate cortactin	178	SAVGFDpYQGKTEK	35.8	5min_batch2
		421	LPSpSPVpYEDAASFK	70.2	1min_batch2
		421	LPSSPVpYEDAASFK	83.4	60min_batch2
		421	TQpTPPVSPAPQPTEERLPSSPVpYEDAASFK	74.4	1min_batch1
		421	TQTPPVSPAPQPTEERLPSpSPVpYEDAASFK	67.3	1min_batch1
		421	TQTPPVSPAPQPTEERLPSSPVpYEDAASFK	76.2	1min_batch2
		446	GPVSGTEPEPVpYSMEAADYR	87.8	15min_batch2
		446	GPVSGTEPEPVpYS*MEAADYR	76.8	60min_batch2
IPI00029702	Isoform 1 of protein tyrosine kinase 2 beta	579	YIEDEDpYYKASVTR	54.8	1min_batch1
		580	YIEDEDYpYKASVTR	37.3	15min_batch1
IPI00029731	60S ribosomal protein L35A	34	IEGVpYAR	35.1	5min_batch2
IPI00029756	Proto-oncogene tyrosine-protein kinase MER	749	KIpYSGDpYYR	48.6	60min_batch1

		749	KIpYSGDYYR	31.1	60min_batch2
		753	IYSGDpYYR	31.7	60min_batch1
		753	KIpYSGDpYYR	48.6	60min_batch1
		753	KIYSGDpYYR	42.3	60min_batch1
		754	KIYSGDYpYR	40.5	15min_batch2
		754	IYSGDYpYR	31.1	5min_batch1
IPI00029769	Isoform P59-HCK of tyrosine-protein kinase HCK	188	TLDNGGFpYISPR	71.0	15min_batch1
		390	VIEDNEpYTAR	64.5	60min_batch1
		390	VIEDNEpYTAREGAK	36.7	5min_batch2
IPI00030320	Probable ATP-dependent RNA helicase DDX6	473	SLYVAEpYHSEPVEDEKP	24.0	1min_batch1
IPI00030887	Tyrosine-protein kinase receptor TYRO3	828	DQPpYSGAGDGSG*MGAVGGTPSDCR	96.6	60min_batch2
IPI00031030	Isoform 1 of amyloid-like protien 2	750	MQNHGpYENPTYK	55.6	60min_batch2
		750	*MQNHGpYENPTYK	38.8	60min_batch1
		755	MQNHGYENPTpYK	56.0	60min_batch1
		755	*MQNHGYENPTpYK	53.2	5min_batch2
		755	*MQNHGYENPTpYKYLEQ*MQI	48.0	15min_batch1
IPI00031068	Isoform 1 of GRB2-associated-binding protein 1	242	HGMNGFFQQQ*MIpYDSPPSR	42.1	15min_batch1
		242	HG*MNGFFQQQMIpYDSPPSR	49.1	15min_batch1
		242	HG*MNGFFQQQ*MIpYDSPPSR	79.4	15min_batch2
		259	APSASVDSSLpYNLPR	115.2	5min_batch2
		373	TASDTDSSpYCIPTAG*MSPSR	80.6	5min_batch2
		406	DASSQDCpYDIPR	46.1	15min_batch2
		406	KDASSQDCpYDIPR	56.7	60min_batch1
		659	SSGSGSSVADERVDpYVVVDQQK	69.9	1min_batch1
IPI00031195	Isoform 1 of vang-like protein 1	344	DSSHNELpYYEEAEHER	21.7	15min_batch1
IPI00031386	Phosphotidylinositol-4,5-bisphosphate 3-kinase catalytic subunit alpha isoform	508	EAGFSpYSHAGLSNR	45.0	5min_batch2
IPI00031407	Enhancer of filamentation 1	166	TGHGYVpYEYPSR	34.3	5min_batch2
		261	QAGRPDLRPEGVpYDIPPTCTKPAGK	20.5	1min_batch1
		317	HQSLSPNHPPPQLGQSVGSQNDApYDVPR	67.8	1min_batch1
		317	RHQSLSPNHPPPQLGQSVGSQNDApYDVPR	49.2	15min_batch1
		345	ANPQERDGVpYDVPLHNPPDAK	40.4	15min_batch1
		345	DGVpYDVPLHNPPDAK	68.9	1min_batch1
IPI00031461	cDNA FLJ60299, highly similar to Rab GDP dissociation inhibitor beta	203	TDDYLDQPCpYETINR	87.9	15min_batch2
IPI00031681	Cell division kinase 2	15	IGEGTpYGVVYKAR	38.5	5min_batch2
IPI00031812	Nuclease-sensitive element-binding protein 1	208	RPQpYSNPPVQGEV*MEGADNQGAGEQGRPVR	75.3	1min_batch1
		208	RPQpYSNPPVQGEVMEGADNQGAGEQGRPVR	55.4	1min_batch1
IPI00032003	Emerin	85	KEDALLpYQSK	60.5	5min_batch2
		94	GYNDDpYYEESYFTTR	87.8	15min_batch1
IPI00032426	Protein MEMO1	210	YSYYDESQGEIpYR	61.3	15min_batch1
IPI00033025	51kDa protein	320	KLAAVTpYNGVDNNK	89.2	5min_batch2
		320	LAAVTpYNGVDNNK	91.4	5min_batch2
IPI00043622	Isoform 1 of ferm domain-containing protein 6	412	DTGPEDSpYSSSAIHR	69.0	5min_batch2
IPI00043978	Isoform 1 of partitioning defective 3 homolog B	1000	DHLEGLpYAK	42.4	5min_batch1
		1000	DGHPLSPERDHLEGLpYAK	23.7	15min_batch1
IPI00044678	Isoform 1 of serine/threonine-protein kinase PFTAIRE-2	63	LGEGSpYATVYK	50.9	5min_batch2
IPI00045423	Isoform 7 of partitioning defective 3 homolog	388	FSPDSQpYIDNR	41.1	60min_batch2
		489	DVTIGGSAPIpYVK	52.4	5min_batch1
		706	ISHSLpYSGIEGLDESPSR	82.1	15min_batch1
		706	RISHpSLpYSGIEGLDESPSR	50.9	5min_batch2
		706	RISHSLpYSGIEGLDESPSR	73.6	15min_batch1
		706	RISHSLpYpSGIEGLDESPSR	45.6	15min_batch2
		997	ERDpYAEIQDFHR	30.7	15min_batch2
		1044	EGH*M*MDALpYAQVK	40.6	5min_batch2
		1044	EGHM*MDALpYAQVK	25.9	60min_batch1
		1044	EGHMMDALpYAQVK	61.6	15min_batch1
		1044	EGH*MMDALpYAQVK	37.7	15min_batch2
		1138	QpYpSSLPR	21.7	5min_batch2
		1161	KNASSVSQDSWEQNpYSPGEGFQSAK	68.3	1min_batch1
		1230	KQPPSEGPSNYDSpYK	21.8	5min_batch2
IPI00053621	Zinc finger protein 99	77	IHpTRENIpYK	21.6	1min_batch1
IPI00054004		65	YFDSGDpYN*MAK	44.5	60min_batch2
IPI00059185	Uncharacterized protein C11ORF52	78	SPGLMSEDSNLHpYADIQVCSRPHAR	46.8	60min_batch1

		78	SPGL*MSEDSNLHpYADIQVCSRPHAR	25.7	5min_batch2
		103	HVHLENATEpYATLR	48.9	1min_batch1
IPI00059292	Protein mago nashi homolog 2	42	YANNSNpYKNDV*MIR	48.1	5min_batch1
		42	YANNSNpYKNDVMIR	30.0	15min_batch1
IPI00059964	Isoform 1 of gamma secretase subunit APH-1A	256	V*MVpYSALR	32.0	60min_batch2
IPI00061178	RNA binding motif protein, X-linked-like 1	134	GGH*MDDGGpYS*MNFN*MSSSR	66.6	60min_batch2
		243	DYTpYRDYGHSSSR	21.2	5min_batch2
IPI00063784	Isoform long of vesicle transport through interaction with T-snares homolog 1B	115	YGIpYAVENEHMNR	23.0	1min_batch1
IPI00064607	Isoform 1 of multiple epidermal growth factor-like domains 10	1061	RDSPpYAEINNSTSANR	84.4	5min_batch2
		1061	DSPpYAEINNSTSANR	73.5	15min_batch2
		1099	LSQDPpYDLPK	35.4	5min_batch2
		1111	NSHIPCHpYDLLPVR	41.8	15min_batch1
IPI00072377	Isoform 1 of protein SET	146	IDFYFDENPpYFENK	57.9	5min_batch2
IPI00099522	Isoform 1 of homeodomain-interacting protein kinase 3	359	TVCSTpYLQpSR	26.1	60min_batch2
		359	TVCSTpYLQSR	37.7	1min_batch2
IPI00099883	Isoform 1 of G-protein coupled receptor family C group 5 member C	312	SSPEQSpYQGD*MpYPTR	43.9	5min_batch2
		312	SSPEQSpYQGD*MYPTR	53.6	5min_batch2
		317	SSPEQSpYQGD*MpYPTR	43.9	5min_batch2
IPI00101049	Putative uncharacterized protein NMD3	236	LISQDIHSNTpYNYK	65.7	60min_batch1
IPI00103013	Sorting Nexin-33	264	LTPTHAASPVpYR	51.6	60min_batch1
IPI00103018	Putative uncharacterized protein KIAA1217	244	NVpYYELNDVR	66.0	1min_batch1
		393	NEGFpYADPYLYHEGR	59.7	0min_batch1
		399	NEGFYADPYLpYHEGR	25.9	1min_batch1
IPI00107633	Isoform 2 of SH3 and multiple ankyrin repeat domains protein 2	228	CFPAGSD*MNSVpYER	67.5	5min_batch2
		228	CFPAGSDMNSVpYER	52.1	15min_batch1
IPI00107698	Fibroblast growth factor receptor substrate 2	306	LVpYENINGLSIPSASGVR	86.8	1min_batch2
IPI00140420	Staphylococcal nuclease domain-containing protein 1	908	ADDADEFGpYSR	64.7	5min_batch2
IPI00149048	Isoform 2 of mitogen-activated protein kinase 7	82	GLCTSPAEHQYFMTEpYVATR	61.8	1min_batch1
		82	GLCTSPAEHQYFMpTEpYVATR	55.8	1min_batch1
		82	GLCTSPAEHQYF*MpTEpYVATR	42.6	15min_batch1
		82	GLCTSPAEHQYF*MTEpYVATR	58.2	60min_batch1
IPI00152093	Isoform 3 of periphilin-1	57	SFpYSSHYAR	43.2	5min_batch2
IPI00152881	Shroom family member 3 protein	501	ESGpYIAPQGACNK	38.0	60min_batch1
IPI00162743	Isoform 2 of suppresion of tumorgenicity 5	81	ENPpYEDVDLK	49.1	5min_batch2
IPI00165157	Sodium potassium-transporting ATPase subunit beta-1-interacting protein 1	160	TSHLQLQPLpYTSG	42.4	15min_batch1
IPI00165946	Isoform 2 of membrane-associated guanylate kinase, WW and PDZ domain-containing protein 1	373	IEDPVpYGIYYVDHINR	42.6	5min_batch1
		373	IEDPVpYGIYYVDHINRK	45.4	0min_batch1
IPI00166680	Isoform 3 of misshapen-like kinase 1	706	SNSAWQIpYLQR	76.0	1min_batch1
IPI00167198	Isoform 2 of U4/U6 small nuclear ribonucleoprotein PRP31	205	IpYEYVESR	24.1	5min_batch2
IPI00168459	Isoform 2 of pleckstrin homology-like domain family B member 2	131	ADFDHpYTGRDSER	21.2	5min_batch2
IPI00169383	Phosphoglycerate kinase 1	196	ELNpYFAK	22.9	5min_batch1
IPI00170865	Isoform 4 of membrane-associated guanylate kinase, WW and PDZ domain-containing protein 3	353	IEDPQpYGTYYVDHLNQK	53.2	15min_batch1
IPI00171134	Isoform 1 of girdin	1765	KTEDTpYFISSAGKPTPGTQGK	67.4	5min_batch2
IPI00171499	Isoform 3 of FAM59A	74	QWTTITAHSLEEGHpYVIGPK	25.0	1min_batch2
		453	SELPpYEELWLEEGKPSHQPLTR	53.4	1min_batch2
IPI00174976	Isoform 1 of MAGUK P55 subfamily member 5	243	VpYESIGQYGGETVK	60.7	60min_batch1
IPI00175416	Isoform 1 of 1-phosphatidylinositol-4,5-bisphosphate phosphodiesterase ETA-1	1275	HATNTVpYETTCTPISK	83.0	5min_batch2
IPI00176458	Isoform 2 of protocadherin-1	1089	LGPLALPEDHpYER	44.2	15min_batch1
IPI00176662		31	LVQSPNSpYFMDVK	69.5	15min_batch1
		31	LVQSPNSpYF*MDVK	55.5	5min_batch2
IPI00176903	Isoform 1 of polymerase and transcript release factor	308	SFTPDHVVpYAR	25.9	0min_batch1
IPI00179053	Isoform 1 of breast cancer anti-estrogen resistance protein 3	266	CLEEHpYGTSPGQAR	37.2	5min_batch1
IPI00179330	Ubiquitin and ribosomal protein S27A precusor	148	CCLTpYCFNKPEDK	37.3	1min_batch1
IPI00180143	Dual specificity protein kinase CLK4	80	YVDEYRNDpYCEGYVPR	36.2	5min_batch2

IPI00181265	cDNA FLJ56060, weakly similar to ferm domain-containing protein 4E	398	SSEVLpYERPQPTPAFSSR	49.6	15min_batch1
IPI00181905	Isoform 1 of actin filament-associated protein 1 like 2	413	VAQQPLSLVGCEVVPDPSPDHLpYSFR	76.6	1min_batch1
IPI00182118	Isoform 2 of probable palmitoyltransferase ZDHHC8	449	EPpSPVRpYDNLSR	41.4	5min_batch2
IPI00182469	Isoform 1AB of catenin delta-1	96	LNGPQDHSHLLpYSTIPR	58.8	15min_batch1
		96	LNGPQDHSHLLpYpSTIPR	34.2	1min_batch1
		213	NFHYPPDGpYSR	28.1	5min_batch2
		217	HpYEDGYPGGSDNYGSLSR	77.1	15min_batch1
		228	HYEDGYPGGSDNpYGpSLSR	68.8	15min_batch2
		228	HYEDGYPGGSDNpYGSLSR	83.8	60min_batch2
		257	QDVpYGPQPQVR	57.5	60min_batch1
		257	APpSRQDVpYGPQPQVR	45.4	1min_batch1
		257	APSRQDVpYGPQPQVR	40.3	5min_batch2
		296	S*MGYDDLDpYGMMSDYGTAR	81.3	1min_batch1
		296	S*MGYDDLDpYG*M*MSDYGTAR	70.1	1min_batch1
		302	S*MGYDDLDYG*M*MSDpYGTAR	69.2	5min_batch2
		334	SYED*MIGEEVPSDQpYYWAPLAQHER	81.2	5min_batch1
		898	SLDNNpYSTPNER	79.5	60min_batch2
IPI00182798	Isoform 1 of PAX-interacting protein 1	704	YTGpYLCR	25.6	5min_batch2
IPI00183046	Isoform 3 of tyrosine-protein phosphatase non-receptor type 6	525	HKEDVpYENLHTK	27.2	15min_batch2
IPI00183445	Isoform 1 of Latrophiliin 1	1337	AEIELLpYK	23.2	5min_batch2
IPI00183526	NCL protein	235	SISLYpYTGEK	31.7	1min_batch2
IPI00185526	Putative uncharacterized protein SAMSN1	247	VHTDFTPSPpYDTDSLK	71.6	1min_batch1
IPI00185919	Isoform 1 of LA-related protein 1	361	THFDpYQFGYR	26.5	5min_batch2
IPI00186826	Ephrin receptor	574	EAEpYSDKHGQYLIGHGTK	42.3	60min_batch1
		590	VpYIDPFTpYEDPNEAVR	70.2	15min_batch2
		596	VpYIDPFTpYEDPNEAVR	70.2	15min_batch2
		596	VYIDPFTpYEDPNEAVR	90.3	1min_batch1
		774	FLEENpSSDPTpYTSSLGGK	76.2	1min_batch1
		774	FLEENSSDPTpYTSSLGGK	114.8	15min_batch1
		935	SQAKPGTPGGTGGPAPQpY	44.4	5min_batch1
IPI00186990	Isoform 1 of GRB2-associated-binding protein 2	293	GSLTGSETDNEDVpYTFK	77.3	1min_batch1
		409	ASSCETpYEYPQR	32.5	5min_batch2
IPI00215637	ATP-dependent RNA helicase DDX3X	69	DKDApYSSFGSR	41.6	5min_batch2
IPI00215716	Isoform B of band 4.1-like protein 3	471	DS*MSAAEVGTGQpYATTK	62.3	5min_batch2
IPI00215948	Isoform 1 of catenin alpha-1	177	NAGNEQDLGIQpYK	89.3	5min_batch1
		619	LVpYDGIR	23.1	0min_batch1
IPI00215949	Isoform 2 of homeodomain-interacting protein kinase 2	361	AVCSTpYLQpSR	43.1	60min_batch1
		361	AVCSTpYLQSR	55.3	5min_batch2
IPI00215965	Isoform A1-B of heterogenous nuclear ribonucleoprotein A1	341	SSGPpYGGGGQYFAKPR	47.6	60min_batch1
		347	SSGPYGGGGQpYFAKPR	74.2	5min_batch2
		357	NQGGpYGGSSSSSSYGSGR	92.4	60min_batch1
IPI00215980	Isoform alpha of poliovirus receptor-related protein 2	408	KSPGGAGGGASGDGGFpYDPK	39.5	5min_batch2
IPI00216008	Isoform long of glucose-6-phosphate 1-dehydrogenase	549	VGFQpYEGTYK	28.7	5min_batch2
		553	VGFQYEGTpYK	44.2	5min_batch2
IPI00216049	Isoform 1 of heterogenous nuclear ribonucleoprotein K	323	GGDL*MApYDRR	28.7	5min_batch2
IPI00216171	Gamma enolase	44	AAVPSGASTGIpYEALELR	105.2	5min_batch2
IPI00216219	Isoforom long of tight junction protein ZO-1	830	LSYLSAPGSEpYS*MYSTDSR	79.7	15min_batch1
		830	LSYLSAPGSEpYSMYSTDSR	72.0	1min_batch1
		895	SSEPVREDSSG*MHHENQTpYPPYSPQAQPQPIHR	25.1	60min_batch1
		895	EDSSGMHHENQTpYPPYSPQAQPQPIHR	26.6	1min_batch1
		1059	DLEQPTpYR	30.2	5min_batch2
		1059	DLEQPTpYRYESSSYTDQFSR	55.2	15min_batch1
		1061	DLEQPTYRpYESSSYTDQFSR	52.2	5min_batch2
		1066	YESSSpYTDQFSR	49.0	5min_batch1
		1140	FEEPAPLSpYDSRPR	46.3	5min_batch2
		1191	LRPEAQPHPSAGPKPAESKQpYFEQYSR	24.4	15min_batch1
		1191	QpYFEQYSR	35.7	60min_batch2
		1195	QYFEQpYSR	28.3	1min_batch2
		1346	SNHpYDPEEDEEYYRK	57.5	15min_batch2
		1354	SNHYDPEEDEEpYYRK	57.1	15min_batch1
		1355	SNHYDPEEDEEYpYRK	56.6	60min_batch2
		1361	QLSpYFDRR	27.7	5min_batch1

		1398	SFENKPPAHIAASHLSEPAKPAHSQNQSNFSSpYSSK	34.4	15min_batch1
IPI00216423	Isoform 3 of intesectin-2	552	LIpYLVPEK	34.7	1min_batch2
		967	EEPEALpYAAVNK	67.5	5min_batch2
		967	REEPEALpYAAVNK	86.3	60min_batch1
IPI00216592	Isoform C1 of heterogenous nuclear ribonucleoproteins C1/C2	124	MpYSYPAR	28.1	1min_batch2
IPI00216969	Isoform IA of proto-oncogene tyrosine-protein kinase ABL1	185	INTASDGKLpYVSSESR	48.4	5min_batch2
		257	LGGGQYGEVpYEGVWK	46.7	1min_batch1
		257	LGGGQYGEVpYEGVWKK	60.2	15min_batch1
		393	LMTGDTpYTAHAGAK	66.7	15min_batch2
		393	L*MTGDTpYTAHAGAK	69.8	5min_batch2
IPI00216991	Isoform 3 of ARF GTPase-activating protein GIT2	469	KQATTNVpYQVQTGSEYTDTSNHSSLK	34.0	1min_batch1
		469	QATTNVpYQVQTGSEYTDTSNHSSLKR	34.0	1min_batch1
		469	QATTNVpYQVQTGSEYTDTSNHSSLK	76.3	1min_batch1
		577	QNSTPESDpYDNTPNDMEPDGMGSSR	54.0	1min_batch1
		577	QNSTPESDpYDNTPND*MEPDGMGSSR	55.6	1min_batch1
		577	QNSTPESDpYDNTPND*MEPDG*MGSSR	41.9	60min_batch2
IPI00217054	Isoform long of E3 ubiquitin-protein ligase SMURF-1	413	IEVSREEIFEEpSpYR	23.6	60min_batch1
IPI00217059	Isoform 2 of coiled-coil domain-containing protein 50	455	NERPARPPPPI*MTDGEDADpYTHFTNQQSSTR	48.4	1min_batch1
IPI00217223	Mulitfunctional protein ADE2	48	TKEVpYELLDSPGK	36.0	5min_batch2
IPI00217418	Isoform 2 of RHO GTPase-activating protein 12	243	ATTPPNQGRPDpSPVpYANLQELK	31.9	15min_batch1
		243	ATpTPPNQGRPDpSPVpYANLQELK	49.0	5min_batch2
		243	ApTTPPNQGRPDpSPVpYANLQELK	39.9	15min_batch2
IPI00217563	Isoform beta-1A of intergrin beta-1	783	WDTGENPIpYK	50.8	60min_batch2
IPI00217872	Isoform 2 of phosphoglucomutase-1	371	IALpYETPTGWK	35.7	1min_batch1
IPI00218487	Gap junction alpha-1 protein	137	FKpYGIEEHGK	39.0	60min_batch1
		247	SDPpYHATSGALpSPAKDCGSQK	29.5	60min_batch1
		247	SDPpYHATSGALSPAK	57.2	15min_batch2
		247	SDPpYHApTSGALSPAK	44.4	60min_batch1
		247	GKSDPpYHATSGALSPAK	64.0	5min_batch2
		265	DCGSQKpYApYFNGCSSPTAPLSP*MSPPGYK	47.4	5min_batch2
		265	DCGpSQKpYAYFNGCSSPTAPLSP*MSPPGYK	47.2	15min_batch2
		267	DCGSQKpYApYFNGCSSPTAPLSP*MSPPGYK	47.4	5min_batch2
		267	YApYFNGCSSPTAPLSPMSPPGYK	37.3	1min_batch1
		267	YApYFNGCSSPTAPLSP*MSPPGYK	35.8	5min_batch2
		286	YAYFNGCSSPTAPLSP*MSPPGpYK	21.6	1min_batch1
		313	QASEQNWANpYSAEQNR	99.0	60min_batch2
		313	QApSEQNWANpYSAEQNR	50.9	1min_batch1
IPI00218570	Phosphoglycerate mutase 2	92	HpYGGLTGLNK	33.1	15min_batch2
IPI00219217	L-lactate dehydrogenase B chain	240	*MVVESApYEVIK	41.2	0min_batch1
		240	MVVESApYEVIK	37.6	1min_batch2
IPI00219299	Talin-2	28	T*MQFEPSTAVpYDACR	59.5	5min_batch2
		1667	ECDpYSIDGINR	42.5	0min_batch1
IPI00219420	Structural maintenance of chromosomes protein 3	560	LFpYHIVDSDEVSTK	27.8	5min_batch2
IPI00219483	Isoform 2 of U1 small nuclear ribonucleoprotein 70kDa	126	EFEVpYGPIKR	25.3	15min_batch1
		146	GpYAFIEYEHER	37.8	1min_batch1
IPI00219622	Proteome subunit alpha type-2	76	HIGLVpYSG*MGPDYR	66.6	1min_batch2
		76	HIGLVpYSGMGPDYR	94.7	1min_batch1
IPI00219757	Bleomycin hydrolase	8	PPYTVVpYFPVR	57.4	1min_batch1
		109	YISLIpYTNYEAGKDDYVK	21.7	1min_batch1
IPI00219798	Isoform 1 of roundabout homolog 1	932	NGLTSTpYAGIR	37.3	60min_batch1
IPI00219852	Isoform B of membrane cofactor protein	384	GKADGGAEpYATYQTK	70.8	60min_batch1
		384	ADGGAEpYATYQTK	73.3	5min_batch1
		387	ADGGAEYATpYQTK	42.7	5min_batch2
IPI00220030	Isoform alpha of paxillin	88	FIHQQPQSSSPVpYGSSAK	59.2	1min_batch2
		88	FIHQQPQSSpSPVpYGSSAK	44.1	5min_batch2
		88	FIHQQPQSpSSPVpYGSSAK	32.7	15min_batch2
		118	VGEEEHVpYSFPNKQK	49.8	5min_batch2
		118	VGEEEHVpYSFPNK	54.0	15min_batch1
IPI00220032	Isoform 2 of catenin delta-2	292	GGSAPEGATpYAAPR	75.7	5min_batch1

		424	ALQSPEHHIDPIpYEDR	34.9	15min_batch1
		499	ASYAAGPASNpYADPYR	59.1	1min_batch1
		516	QLQYCPSVESPpYSK	34.9	1min_batch1
IPI00220490	Isoform 3 of SH3 and multiple ankyrin repeat domains protein 2	371	YSLDSEDLpYSR	86.5	15min_batch1
		371	ELDRYSLDSEDLpYSR	26.3	5min_batch2
		393	GQ*MPENPpYSEVGK	52.7	60min_batch2
		393	RGQ*MPENPpYSEVGK	44.5	5min_batch2
		393	RGQMPENPpYSEVGK	65.6	5min_batch2
		393	GQMPENPpYSEVGK	49.6	5min_batch1
IPI00221067	Isoform 2 of receptor-type tyrosine protein phosphatase alpha	789	VVQEYIDAFSDpYANFK	96.9	1min_batch1
IPI00221089	40S ribosomal protien S13	38	LTSDDVKEQIpYK	47.0	60min_batch1
IPI00221316	Isoform C of protein sprouty homolog 4	52	TSHVENDpYIDNPSLALTTGPK	48.3	60min_batch1
IPI00240909		245	YApYYDTER	25.7	5min_batch2
IPI00253050	Line-1 type transposase domain-contianing protein 1	24	ENITpYMKR	29.3	60min_batch1
IPI00257508	Dihydropyrimidinase-related protein 2	499	GLpYDGPVCEVSVTPK	78.8	5min_batch2
IPI00260090	cDNA FLJ60834, highly similar to engulfment and cell motility protein 2	717	EPSSYDFVpYHYG	28.7	5min_batch2
		719	EPSSYDFVYHpYG	23.4	1min_batch1
IPI00289258	cDNA FLJ9037, highly simlar to myosin-10	1128	CSVGTpYNSpSGAYR	38.5	5min_batch2
		1128	CSVGTpYNSSGApYR	70.3	5min_batch1
		1128	CSVGTpYNSSGAYR	73.8	1min_batch2
		1134	CSVGTpYNSSGApYR	70.3	5min_batch1
		1134	CSVGTYNpSSGApYR	47.2	5min_batch2
		1134	CSVGTYNSSGApYR	110.2	1min_batch2
IPI00289329	Ephrin type-B receptor 3	600	LQQpYIAPG*MK	20.1	60min_batch2
		792	FLEDDPSDPTpYTSSLGGK	92.4	15min_batch1
IPI00289334	Isoform 1 of filamin-B	2502	SSTETCpYSAIPK	23.4	5min_batch1
IPI00289499	Bifunctional purine biosynthesis protein PURH	290	VC*MVpYDLYK	33.8	5min_batch2
IPI00289822	Isoform RMO1AB of Ras-associated and pleckstrin homology domains-containing protein 1	576	TESApYDWTSLSSSSIK	99.8	1min_batch2
IPI00290039	Isoform 1 of CUB domain-containing protein 1	707	TNKGPAVGIpYNGNINTEMPR	37.1	1min_batch1
		707	GPAVGIpYNGNINTE*MPR	81.5	1min_batch1
		707	GPAVGIpYNGNINTEMPR	52.8	1min_batch2
IPI00290158	Isoform 7 of serine/threonine-protein kinase MARK2	526	DQQNLPpYGVTPASPSGHSQGR	60.2	1min_batch2
IPI00290337	Epidermal growth factor receptor kinase substrate 8	527	HIDRNpYEPLK	27.6	60min_batch1
IPI00291175	Isoform 1 of vinculin	692	NPGNQAApYEHFET*MK	61.7	15min_batch2
		692	NPGNQAApYEHFETMK	70.7	15min_batch1
		822	SFLDSGpYR	38.8	60min_batch2
IPI00291802	Isoform 3 of LIM domain only protein 7	185	KAQSNPpYYNGPHLNLK	35.0	5min_batch2
		186	AQSNPYpYNGPHLNLK	26.7	5min_batch2
IPI00291939	Structural maintenance of chromosomes protein 1A	186	AEEDTQFNpYHR	29.1	5min_batch2
		714	LKpYSQSDLEQTK	60.0	5min_batch2
IPI00292856	Isoform long of E3 ubiquitin-protein ligase CBL-B	889	TSQDpYDQLPSCSDGSQAPARPPKPRPR	31.2	60min_batch1
IPI00293126	Tubulin-folding cofactor E	98	LGEpYEDVSR	57.0	5min_batch1
IPI00293451	Tight junction protein ZO-3	364	ESSYDIpYRVPSSQS*MEDR	45.9	1min_batch1
		364	ESSYDIpYRVPSSQSMEDR	54.5	1min_batch1
IPI00293655	ATP-dependent RNA helicase DDX1	628	VWpYHVCSSR	32.3	5min_batch2
IPI00294250	Ephrin type-A receptor 1	781	LLDDFDGTpYETQGGK	76.9	1min_batch1
			LLDDFDGTpYETQGGKIPIR	54.1	60min_batch1
IPI00295698	Catonic amino acid transporter 3	615	TVDLDPGTLpYVHSV	51.5	1min_batch1
IPI00296114	Large neutral amino acids transported small subunit 2	519	GSGTEEANEDMEEQQQP*MpYQPTPTK	59.5	1min_batch1
		519	GSGTEEANEDMEEQQQPMpYQPTPTK	73.6	1min_batch1
		519	GSGTEEANED*MEEQQQP*MpYQPTPTK	41.3	5min_batch2
IPI00296283	Mitogen-activated protein kinase 12	185	QADSEMTGpYVVTR	60.7	5min_batch1
		185	QADSE*MTGpYVVTR	49.7	60min_batch2
		185	QADSE*MpTGpYVVTR	20.2	5min_batch1
IPI00296784	Isoform 1 of marvel domain-contianing protein 2	23	RYDEVPSDLPpYQDTTIR	40.3	1min_batch1
IPI00296913	APD-sugar pyrophosphatase	74	TLHpYECIVLVK	36.8	1min_batch1
IPI00296992	Isoform long of tyrosine-protein kinase receptor UFO	702	IYNGDpYYR	38.2	5min_batch1
		702	KIYNGDpYYR	43.5	1min_batch2
		702	IYNGDpYpYR	25.5	5min_batch2

		702	KIYNGDpYpYR	27.0	5min_batch1
		703	IYNGDpYpYR	25.5	5min_batch2
		703	KIYNGDYpYR	38.1	5min_batch1
		703	KIYNGDpYpYR	27.0	5min_batch1
IPI00298285	Isoform 1 of receptor tyrosine-protein kinase ERBB-3	1276	DGGGPGGDpYAA*MGACPASEQGpYEE *MR	33.7	60min_batch2
		1289	DGGGPGGDYAA*MGACPASEQGpYEE* MR	82.6	1min_batch1
		1289	DGGGPGGDYAA*MGACPASEQGpYEE MR	81.9	1min_batch1
		1289	DGGGPGGDpYAA*MGACPASEQGpYEE	33.7	60min_batch2
		1307	AFQGPGHQAPHVHpYAR	33.5	5min_batch2
		1328	SLEATDSAFDNPDpYWHSR	93.0	60min_batch1
IPI00298347	Isoform 2 of tyrosine-protein phosphatase non-receptor type 11	62	IQNTGDpYYDLYGGEK	104.2	15min_batch1
		580	VpYENVGLMQQQK	56.7	60min_batch1
		580	VpYENVGL*MQQQK	67.9	5min_batch2
IPI00298625	Isoform LYN A of tyrosine-protein kinase LYN	193	SLDNGGpYYISPR	54.9	5min_batch2
		194	SLDNGGYpYISPR	66.4	60min_batch1
IPI00298860	cDNA FLJ8440, highly similar to human lactoferrin	546	YpYGYTGAFR	42.8	5min_batch1
		548	YYGpYTGAFR	34.0	5min_batch1
IPI00298994	Talin-1	26	TMQFEPSTMVpYDACR	83.9	1min_batch1
		26	T*MQFEPST*MVpYDACR	76.3	1min_batch1
		26	T*MQFEPSTMVpYDACR	98.3	15min_batch1
		70	ALDpYYMLR	43.7	1min_batch2
		70	ALDpYY*MLR	28.3	5min_batch2
		71	ALDYpYMLR	23.3	1min_batch1
IPI00299588	ARGBPIB protein	157	HTPPTIGGSLPpYR	44.9	1min_batch1
IPI00299699	Neural proliferation differentiation and control protien 1	249	LAQSAE*MpYHYQHQR	34.5	5min_batch2
IPI00299904	Isoform 1 of DNA replication licensing factor MCM7	492	CSILAAANPApYGR	21.2	1min_batch1
IPI00299977	14kDA phosphohistidine phosphatase	125	AKYPDYEVTWANDGpY	71.3	1min_batch1
IPI00300384	Receptor tyrosine-protein kinase ERBB-2	877	LLDIDETEpYHADGGK	75.7	5min_batch2
		877	LLDIDETEpYHADGGKVPIK	66.5	5min_batch2
IPI00301058	Vasodilator-stimulated phosphoprotein	39	VQIpYHNPTANSFR	73.7	15min_batch2
IPI00301561	Thyroid receptor-interacting protein 6	55	VNFCPLPSEQCpYQAPGGPEDR	34.4	15min_batch1
		123	QApYEPPPPPAYR	42.1	5min_batch2
		131	QAYEPPPPPApYR	42.5	15min_batch1
IPI00301987	Isoform 1 of WD repeat-containing protein MIO	235	*MFVNTKAVQGVpTVDPpYFHDR	28.1	1min_batch1
IPI00302690	Actin-related protein 10	4	PLpYEGLGSGGEK	31.9	5min_batch2
IPI00302925	59kDa protein	30	HFSGLEEAVpYR	51.2	15min_batch1
IPI00305610	Scavenger receptor class F member 2	623	SASSVEGPGGALpYAR	65.8	5min_batch2
IPI00306436	Isoform del-701 of signal transducer activator of trancription 3	704	YCRPESQEHPEADPGAAPpYLK	38.8	15min_batch1
IPI00307545	Tensin-1	796	SYSPpYDYQPCLAGPNQDFHSK	52.1	1min_batch1
		798	SYSPYDpYQPCLAGPNQDFHSK	48.9	15min_batch1
		903	AASDGQpYENQSPEATSPR	47.5	5min_batch2
		1254	HPAGVpYQVSGLHNK	36.8	5min_batch1
		1323	HVApYGGpYSTPEDR	61.9	5min_batch2
		1323	HVApYGGYSTPEDR	39.7	0min_batch1
		1326	HVApYGGpYSTPEDR	61.9	5min_batch2
		1326	HVAYGGpYSTPEDR	80.5	60min_batch1
		1404	AGSLPNpYATINGK	31.8	5min_batch1
IPI00307829	Isoform 1 of cingulin-like protein 1	108	ENSEELQLPENPpYAQPSPIR	73.6	1min_batch2
IPI00328180	Isoform 1 of Ras-related protein Rab-34	247	INSDDSNLpYLTASK	59.2	5min_batch1
IPI00328218	Isoform 2 of lipolysis-stimulated lipoprotein receptor	272	CPCCPDKCCCPEALpYAAGK	43.2	1min_batch1
		272	CCCPEALpYAAGK	60.9	5min_batch1
		286	AATSGVPSIpYAPSTYAHLpSPAK	53.5	15min_batch1
		286	AATSGVPSIpYAPSTpYAHLSPAK	40.1	5min_batch2
		286	AATSGVPSIpYAPSTYAHLSPAK	42.6	1min_batch1
		291	AATSGVPSIpYAPSTpYAHLSPAK	40.1	5min_batch2
		348	VLpYY*MEK	24.9	5min_batch2
		493	DPHpYDDFR	20.4	5min_batch1
		493	SRDPHpYDDFR	47.5	15min_batch2
		528	SGDLPpYDGR	22.4	60min_batch2
IPI00328587		44	AAVPSGASTGIpYEVLELQDNDK	32.4	1min_batch1
IPI00328737	Isoform 1 of zinc finger protein 598	306	RNEGVVGGEDpYEEVDR	78.5	5min_batch2
IPI00328825	Isoform 1 of NEDD4-binding protein 2	599	IELCApYSCEDR	56.9	1min_batch1
IPI00329027	P2Y purinoceptor 8	272	LFYGKSYpYHVYK	23.6	1min_batch2
IPI00329033	Protein kinase C delta type	90	IQEpYIIAFAKEK	23.9	60min_batch1
IPI00329236	Faser syndrome 1 protein isoform precursor	313	SDSASSEPVGIpYQGFEK	77.3	15min_batch2

		313	RSDSASSEPVGIpYQGFEK	99.8	1min_batch1
		334	TGVAGED*MQDNSGTpYGK	35.4	5min_batch2
IPI00329327	Faser syndrome 1 protein isoform 1 precursor	3986	NVNILSEPEAApYTFK	55.6	15min_batch1
IPI00329488	Isoform IB of tyrosine-protein kinase ABL2	718	CpYGGSFAQR	26.8	5min_batch1
IPI00329526	Cell division cylce protein 20 homolog	152	VLpYSQKApTPGSSR	35.4	60min_batch1
IPI00329672	Myosin-IE	7	GVpYQYHWQSHNVK	24.0	15min_batch1
		990	SLpYTS*MARPPLPR	28.4	5min_batch2
		990	SLpYTSMARPPLPR	44.4	1min_batch1
IPI00329801	Annexin A5	94	LYDApYELKHALK	28.4	1min_batch1
IPI00332841	Guanine nucleotide-releasing factor 2 isoform B	347	QDFDVDCpYAQR	61.7	60min_batch1
IPI00334294	Isoform 1 of RHO/CDC42/RAC GTPase activating protein RICS	1188	YNTpYVAPGR	48.8	5min_batch1
		1353	SLpYSYAGLAPRPR	33.6	5min_batch2
IPI00334627	Putative annexin A2-like protein	24	LSLEGDHSTPPSApYGSVK	57.5	1min_batch1
		30	ApYTNFDAER	62.5	5min_batch2
IPI00334715	Isoform 1 of glucocorticoid receptor DNA-binding factor 1	1087	SVSSSPWLPQDGFDPSDpYAEPMDAVVKPR	50.4	15min_batch2
		1087	SVSSSPWLPQDGFDPSDpYAEP*MDAVVKPR	53.0	15min_batch2
		1105	NEEENIpYSVPHDSTQGK	78.8	0min_batch1
IPI00335824	Isoform short of tight junction protein ZO-1	923	IDSPGFKPASQQVpYR	39.8	1min_batch1
		923	IDpSPGFKPASQQVpYR	26.7	0min_batch1
IPI00337612	Discoidin, CUB and LCCL domain-contaiing protien 1	578	GSTFRPMDTDAEEAGVSTDAGGHpYDCPQR	45.1	1min_batch1
		578	GSTFRP*MDTDAEEAGVSTDAGGHpYDCPQR	57.1	1min_batch1
		578	KGSTFRPMDTDAEEAGVSTDAGGHpYDCPQR	60.8	1min_batch1
		578	KGSTFRP*MDTDAEEAGVSTDAGGHpYDCPQR	62.6	1min_batch1
		621	AHTFSAQSGpYRVPGPQPGHK	27.4	1min_batch2
			AHTFSAQSGpYR	60.1	15min_batch2
		652	HSLSSGGFSPVAGVGAQDGDpYQRPHSAQPADR	54.1	15min_batch1
		696	AVSALATESGHPDSQKPPTHPGTSDSpYSAPR	30.3	15min_batch2
IPI00374301	Isoform 1 of uncharactized protein C6ORF132	894	HKAPGSADpYGFAPAAGR	47.8	5min_batch2
IPI00376221	Isofrm 1 of band 4.1-like protein 5	499	LPGLGEPEVEpYETLKDTSEK	33.4	1min_batch1
IPI00376229	Isoform 1 of phosphofurin acidic cluster sorting protein 1	251	IpYSLSSQPIDHEGIK	33.9	1min_batch1
IPI00376306	Isoform 2 of membrane-associated guanylate kinase, WW and PDZ domain-containing protein 2	362	IDDPIpYGTYYVDHINR	57.8	5min_batch2
		362	IDDPIpYGTYYVDHINRR	54.2	5min_batch2
IPI00376955	Serine/Threonine-protein kinase PCTAIRE-2	203	LGEGpTpYATVYK	22.0	15min_batch1
IPI00382470	Isoform of heat shock protein HSP 90-alpha	614	HIpYYITGETK	45.2	60min_batch1
IPI00382749	Ribosomal protein L15	59	QGpYVIYR	22.1	5min_batch1
IPI00382946	CCDC123 protein	128	GGHSDDLpYAVPHR	58.2	5min_batch2
IPI00383423	Isoform of coiled-coil domain-containing protein 50	145	AYADSYpYYEDGG*MKPR	39.9	5min_batch2
		145	AYADSYpYYEDGGMKPR	74.2	15min_batch1
		146	AYADSYYpYEDGG*MKPR	22.8	1min_batch1
IPI00384529	Isoform of protein cordon-bleu	867	TSSQpYVASAIAK	23.3	0min_batch1
IPI00384861	Isoform 1 of ARF GTPase-activating protein GIT1	545	LQPFHSTELEDDAIpYSVHVPAGLYR	47.2	1min_batch1
IPI00385317	Isoform 2 of tensin-like C1 domain-containing phosphatase	483	GPLDGSPpYAQVQRPPR	22.6	1min_batch1
IPI00394952	cDNA FLJ56184, highly similar to proto-oncogene tyrosine-protein kinase LCK	192	NLDNGGFpYISPR	73.0	15min_batch1
		505	SVLEDFFTATEGQpYQPQP	100.5	0min_batch2
IPI00395663	Ankyrin repeat and SAM domain-containing protein 1A	455	EEDEHPpYELLLTAETK	37.0	15min_batch2
		455	EEDEHPpYELLLTAETKK	27.6	1min_batch2
		834	IIASLADRPpYEEPPQKPPR	49.2	1min_batch1
IPI00395903	Transmembrane protein 106B	50	NGDVSQFPpYVEFTGR	61.3	1min_batch1
IPI00396130	Isoform 2 of P130CAS-associated protein	456	GEGLpYADPYGLLHEGR	77.1	1min_batch1
		522	AAGGGGPLpYGDGYGFR	52.4	5min_batch2
IPI00396378	Isoform B1 of heterogenous nuclear ribonucleoproteins A2/B1	331	NMGGPpYGGGNYGPGGSGGSGGYGGR	72.0	1min_batch1
		331	N*MGGPpYGGGNYGPGGSGGSGGYGGR	129.3	5min_batch2
		336	NMGGPYGGGNpYGPGGSGGSGGYGGR	96.0	1min_batch1
		336	N*MGGPYGGGNpYGPGGSGGSGGYGGR	110.1	5min_batch2

		347	NMGGPYGGGNYGPGGSGGSGGpYGGR	110.0	15min_batch1
IPI00396435	Putative pre-mRNA-splicing factor ATP-dependent RNA helicase DHX15	13	HRLDLGEDpYPSGK	59.8	15min_batch2
IPI00396634	Isoform 1 of uncharactized protein KIAA1671	1032	ATFFAVTpYQIPNTQK	66.4	1min_batch1
IPI00397526	Isoform 1 of myosin-10	22	AVIpYNPATQADWTAK	69.6	1min_batch1
		1415	ALApYDKLEK	40.0	5min_batch1
IPI00409640	Isoform 1 of lipolysis-stimulated lipoprotein receptor	372	SSSAGGQGSpYVPLLR	54.1	5min_batch1
IPI00410034	Isoform 1 of sodium-coupled neutral amino acid transporter 2	20	FSISPDEDSSSpYSSNSDFNYSYPTK	76.3	1min_batch1
		28	FSISPDEDSSSYSSNSDFNpYSYPTK	78.5	15min_batch1
		41	SHpYADVDPENQNFLLESNLGK	83.4	1min_batch1
IPI00410693	Isoform 1 of plasminogen activator inhibitor 1 RNA-binding protein	207	SSFSHpYSGLK	32.8	5min_batch2
IPI00411452	Putative uncharacterized protein DOCK11	1223	GSLSTDKDTApYGSFQNGHGIK	25.4	1min_batch1
IPI00411901	Isoform 1 of disks large homolog 5	1197	NPIpYTVR	24.7	5min_batch2
IPI00412752	Signal transducer and activator of transcription 3 isoform 3	705	YCRPESQEHPEADPGSAAPpYLK	54.0	5min_batch2
IPI00412771	CD2-associated protein	541	DTCpYSPKPSVYLSTPSSASK	35.0	1min_batch1
IPI00413641	Aldose reductase	190	YKPAVNQIECHPpYLTQEK	27.2	1min_batch1
IPI00414123	Dihydropyrimidinase-related protein 1	504	GMYDGPVpYEVPATPK	71.8	15min_batch1
		504	G*MYDGPVpYEVPATPK	68.4	1min_batch2
IPI00414676	Heat shock protein HSP 90-beta	484	SIpYYITGESK	41.3	0min_batch1
		485	SIYpYITGESK	28.1	1min_batch2
		596	LVSSPCCIVTSTpYGWTAN*MER	60.5	1min_batch1
IPI00418471	Vimentin	53	SLpYASSPGGVYATR	73.1	1min_batch1
		61	SLYASSPGGVpYATR	51.2	1min_batch2
IPI00418545	Isoform 2 of RHO guanine nucleotide exchange factor 5	292	RTEELIpYLSQK	37.2	5min_batch2
IPI00419100	Isoform 1 of FYVE, RHOGEF and PH domain-containing protein 6	748	SVTSLCAPEpYENIR	40.2	15min_batch1
		760	HYEEIPEpYENLPFI*MAIR	38.1	5min_batch2
IPI00419373	Isoform 1 of heterogenous nuclear ribonucleoprotein A3	360	SSGSPpYGGGYGSGGGSGGYGSR	111.4	60min_batch2
		364	SSGSPYGGGpYGSGGGSGGYGSR	83.4	5min_batch1
		373	SSGSPYGGGYGSGGGSGGpYGSR	118.0	5min_batch2
IPI00419836	Isoform 1 of discoidin, CUB and LCCL domain-containing protein 2	565	TEGTpYDLPYWDR	39.1	5min_batch2
		732	TDSCSSAQAQpYDTPK	66.1	60min_batch2
		750	AGKPGLPAPDELVpYQVPQSTQEVSGAGR	53.4	1min_batch1
IPI00419933	Isoform 1 of pleckstrin homology domain-containing family A member 7	656	SADDTpYLQLKK	42.6	5min_batch2
		665	DLEpYLDLK	33.9	5min_batch2
IPI00431025	Isoform 1 of ABL interactor 1	213	TLEPVKPPTVPNDpYMTSPAR	30.5	1min_batch1
		213	TLEPVKPPTVPNDpY*MTSPAR	49.1	5min_batch2
IPI00435208	Isoform 1 of connnector enhancer of kinase suppressor Ras 2	821	CHLQDHpYGPYPLAESER	43.8	1min_batch1
IPI00435947	Isoform 1 of WAS/WASL-interacting protein family member 2	74	GSSGGpYGSGGAALQPK	52.2	60min_batch1
		346	DAPPPPPPpYR	28.0	5min_batch2
IPI00438229	Isoform 1 of transcription intemediary factor 1-beta	458	QGSGSSQP*MEVQEGYGFGSGDDPpYSSAEPHVSGVK	69.3	5min_batch2
IPI00438286	ERBB2IP protein	1104	RAQIPEGDpYLSYR	49.2	15min_batch1
		1104	AQIPEGDpYLSYR	61.2	15min_batch2
IPI00440932	Isoform 1 of disintegrin and metalloproteinase domain-containing protein 9	769	HVSPVpTPPREVPIpYANR	46.2	1min_batch1
IPI00442025	Isoform 3 ov activated CDC42 kinase 1	937	VSSTHpYYLLPERPSYLER	32.6	1min_batch2
IPI00442210	Isoform 1 of ABL interactor 2	213	TLEPVRPPVVPNDpYVPSPTR	28.7	1min_batch1
IPI00444262	cDNA FLJ45706 FIS, clone FEBRA2028457, highly similar to nucleolin	295	NLPpYKVTQDELK	67.9	1min_batch2
IPI00453473	Histone H4	52	ISGLIpYEETR	51.6	60min_batch1
IPI00453476	29kDa protein	26	FSGWpYDADLSPAGHEEAK	51.4	5min_batch2
		26	FSGWpYDADLSPAGHEEAKR	77.4	1min_batch1
IPI00455851	KIAA1688 protein	448	SGDpYST*MEGPELR	58.0	5min_batch1
IPI00464978	Insulin receptor substrate 2 insertion mutant (fragement)	186	AAAGDAPPAAAPAASCSASLPGALGGSAGAAGAEDSpYGLVAPATAAYR	75.4	60min_batch2
		542	DGGGGGEFpYGpY*MT*MDRPLSHCGR	36.7	5min_batch2
		544	DGGGGGEFpYGpY*MT*MDRPLSHCGR	36.7	5min_batch2
		544	DGGGGGEFYGpY*MT*MDRPLSHCGR	64.5	1min_batch1
		544	DGGGGGEFYGpYMT*MDRPLSHCGR	87.5	15min_batch1

		544	DGGGGGEFYGpYMTMDRPLSHCGR	73.3	15min_batch1
		544	DGGGGGEFYGpY*MTMDRPLSHCGR	59.7	15min_batch2
		600	QRPVPQPSSASLDEpYTLMR	60.8	15min_batch1
		600	QRPVPQPSSASLDEpYTL*MR	77.6	15min_batch2
		634	VAYHPYPEDpYGDIEIGSHR	78.1	5min_batch2
		655	SSSSNLGADDGpYMP*MTPGAALAGSG SGSCR	116.1	60min_batch1
		655	SSSSNLGADDGpY*MP*MTPGAALAGSG SGSCR	96.6	5min_batch1
		655	SSSSNLGADDGpYMPMTPGAALAGSGS GSCR	97.2	1min_batch1
		655	SSSSNLGADDGpY*MPMTPGAALAGSG SGSCR	83.0	1min_batch2
		677	SDDpYMPMSPASVSAPK	105.6	1min_batch1
		677	SDDpYMP*MSPASVSAPK	72.9	60min_batch1
		677	SDDpY*MPMSPASVSAPK	58.5	60min_batch1
		677	SDDpY*MP*MSPASVSAPK	72.8	5min_batch1
		744	ASSPAESSPEDSGpY*MR	80.1	5min_batch2
		744	ASSPAESSPEDSGpYMR	85.7	1min_batch2
		744	ASSPAESpSPEDSGpYMR	60.2	1min_batch2
		744	ASSPAESpSPEDSGpY*MR	57.2	60min_batch1
		744	ASpSPAESSPEDSGpYMR	42.5	60min_batch1
		744	ASpSPAESSPEDSGpY*MR	35.5	5min_batch1
		816	APpYTCGGDSDQYVL*MSSPVGR	31.2	5min_batch2
		825	SYKAPYTCGGDSDQpYVLMSSPVGR	39.1	15min_batch1
		825	APYTCGGDSDQpYVL*MSSPVGR	74.8	1min_batch2
		825	APYTCGGDSDQpYVLMSSPVGR	98.2	15min_batch2
		825	SYKAPYTCGGDSDQpYVL*MSSPVGR	58.9	1min_batch1
		980	SPLSDpY*MNLDFSSPK	68.1	5min_batch1
IPI00470360	Isoform 1 of kin of IRRE-like protein 1	572	AIpYSSFKDDVDLK	78.4	15min_batch1
		605	EEYE*MKDPTNGpYYNVR	41.4	1min_batch1
		606	EEYEMKDPTNGYpYNVR	44.6	1min_batch1
		622	AVLpYADYR	27.2	5min_batch1
		721	TPpYEAYDPIGK	44.4	60min_batch1
		724	TPYEApYDPIGK	40.8	0min_batch1
		745	FSYTSQHSDpYGQR	39.1	5min_batch2
IPI00470838	Isoform 1 of DENN domain-containing protein 2C	195	SLENIpYSEPEGQECGPSINPLPKPR	30.4	5min_batch2
IPI00478817	Isoform 2 of RHO guanine nucleotide exchange factor 10-like protein	152	NLLpYEDAHR	35.5	60min_batch2
IPI00478892	Leucine-rich repeats and immunoglobin-like domains protein 2	912	VICSDCYDNANIpYSR	82.1	15min_batch1
IPI00552750	Activated CDC42 kinase 1	574	KPTpYDPVSEDQDPLSSDFK	50.0	1min_batch1
		574	KPTpYDPVSEDQDPLSSDFKR	75.7	1min_batch1
IPI00554648	Keratin, type II cytoskeletal 8	25	AFSSRpSpYTSGPGSR	28.6	5min_batch2
IPI00554711	Junction plakoglobin	20	VTEWQQTpYTYDSGIHSGANTCVPSVSS K	81.0	15min_batch1
		549	HVAAGTQQPpYTDGVR	46.2	60min_batch1
IPI00641339	cDNA FLJ55515, highly similar to breast cancer anti-estrogen resistance protein 1	306	GLPPSNHHAVpYDVPPSVSK	37.4	15min_batch1
		387	RPGPGTLpYDVPR	45.2	60min_batch1
IPI00643785	Myeloid/Lymphoid or mixed-lineage leukemi	76	YSLpYEVHVSGEER	46.0	1min_batch1
IPI00644231	Isoform 1 of cytoplasmic FMR1-interacting protein 1	108	CNEQPNRVEIpYEK	37.7	60min_batch2
IPI00644865	FERM domain-containing protein 4A	861	SLESDQEGHpYSVK	26.3	5min_batch2
IPI00654623	Isoform 2 of tensin-3	114	WDpSYENLSADGEVLHTQGPVDGSLpY AK	58.2	1min_batch1
		361	QQQMVVAHQpYSFAPDGEAR	29.2	1min_batch1
		361	QQQ*MVVAHQpYSFAPDGEAR	32.9	5min_batch1
		540	LSLGQpYDNDAGGQLPFSK	90.0	1min_batch1
		540	KLSLGQpYDNDAGGQLPFSK	74.7	15min_batch1
		615	ES*MCSTPAFPVSPETPpYVK	47.5	5min_batch2
		615	ESMCSTPAFPVSPETPpYVK	49.2	1min_batch1
IPI00737545	Tyrosine-protein kinase SGK269	387	EIEPNpYESPSSNNQDKDSSQASK	48.6	60min_batch2
		462	ASTDVAGQAVTINLVPTEEQAKPpYR	96.5	1min_batch1
		616	FNSYNNAG*MPPFPIIIHDEPTpYAR	80.0	1min_batch1
		635	NAIKVPIVINPNApYDNLAIYK	30.4	1min_batch1
		635	VPIVINPNApYDNLAIYK	90.8	1min_batch1
		635	VPIVINPNApYDNLAIpYK	31.1	15min_batch2
		641	VPIVINPNApYDNLAIpYK	31.1	15min_batch2
		797	ACpSVEELpYAIPPDADVAK	56.5	1min_batch1
		880	STSSPpYHAGNLLQR	55.9	5min_batch2
		1107	EDGKEDISDP*MDPNPCSATpYSNLGQS R	67.8	5min_batch2
IPI00739386	Tyrosine-protein kinase SGK223	132	QEDAPVVpYLGSFR	58.2	1min_batch1

		197	EKPSFPpYQDRPSTQESFR	21.5	15min_batch1
		253	CSPSGDSEGGEpYCSILDCCPGSPVAK	67.5	15min_batch1
		413	CLGLTGEPQPPAHPQEATQPEPIpYAEST KR	75.7	15min_batch1
		413	CLGLTGEPQPPAHPQEATQPEPIpYAEST K	69.2	1min_batch1
IPI00744706	Isoform 3 of spectrin alpha chain, brair	1053	QEQIDNQpYHSLLELGEK	23.8	15min_batch1
IPI00746301	Cyclin-dependent kinase-like 5	173	NLSEGNNANYTEpYVATR	91.2	15min_batch2
IPI00760833	Isoform 1 of ankyrin repeat domain-containing protein 26	296	KNLEATpYGTVR	35.6	60min_batch1
IPI00784156	Isoform 1 of AP-2 complex subunit beta	276	DSDpYYN*MLLK	39.3	5min_batch2
IPI00784186		364	LRLDTASSNGpYQRPGSVVAAK	28.3	15min_batch1
		364	LDTASSNGpYQRPGSVVAAK	59.5	60min_batch1
IPI00797763	Cingulin	105	GANDQGASGALSSDLELPENPpYSQVK	84.5	60min_batch2
IPI00829652	Putative RNA-binding protein 16	17	TFNSELYSLNDpYKPPISK	60.0	1min_batch1
IPI00844578	ATP dependent helicase A	148	GANLKDpYYSR	26.4	5min_batch2
