# Supplementary material for: Tyrosine Phosphorylation Profiling in FGF-2 Stimulated Human Embryonic Stem Cells
Source: PLoS One. 2011 Mar 17;6(3):e17538. doi: 10.1371/journal.pone.0017538 (PMC3060089; doi:10.1371/journal.pone.0017538)
Supplement: Table S2 — List of cumulative phosphopeptides detected from FGF-2 stimulated hESC. (PDF) [file pone.0017538.s005.pdf]

IPI acession no.	Gene name	Motif pattern	Motif number
IPI00737545	NKF3 kinase family member	...E..yA.....	1
IPI00376955	PCTAIRE protein kinase 2	...E..yA.....	1
IPI00043978	ISOFORM 1 OF PARTITIONING DEFECTIVE 3 HOMOLOG B	...E..yA.....	1
IPI00307829	cingulin-like 1	...E..yA.....	1
IPI00016932	inositol polyphosphate phosphatase-like 1	...E..yA.....	1
IPI00023974	pituitary tumor-transforming 1 interacting protein	...E..yA.....	1
IPI00328218	ISOFORM 2 OF LIPOLYSIS-STIMULATED LIPOPROTEIN RECEPTOR	...E..yA.....	1
IPI00023704	LIM domain containing preferred translocation partner in lipoma	...E..yA.....	1
IPI00002966	heat shock 70kDa protein 4	...E..yA.....	1
IPI00029731	ribosomal protein L35a	...E..yA.....	1
IPI00023942	ISOFORM 2 OF SYNDECAN-3.	...E..yA.....	1
IPI00103018	PUTATIVE UNCHARACTERIZED PROTEIN KIAA1217	...E..yA.....	1
IPI00739386	homolog of rat pragma of Rnd2	...E..yA.....	1
IPI00396130	SNAP25-interacting protein	...E..yA.....	1
IPI00007248	pleckstrin homology domain containing, family A member 6	...E..yA.....	1
IPI00011736	phosphoinositide-3-kinase, regulatory subunit 2 (beta)	...E..yA.....	1
IPI00737545	NKF3 kinase family member	...E..yA.....	1
IPI00044678	PFTAIRE protein kinase 2	...E..yA.....	1
IPI00028931	desmoglein 2	...E..yA.....	1
IPI00045423	ISOFORM 7 OF PARTITIONING DEFECTIVE 3 HOMOLOG.	...E..yA.....	1
IPI00064607	multiple EGF-like-domains 10	......yD.P...	2
IPI00011652	embryonal Fyn-associated substrate	......yD.P...	2
IPI00011652	embryonal Fyn-associated substrate	......yD.P...	2
IPI00026889	disabled homolog 1 (Drosophila)	......yD.P...	2
IPI00011998	BREAST CANCER ANTI-ESTROGEN RESISTANCE 1.	......yD.P...	2
IPI00031068	GRB2-associated binding protein 1	......yD.P...	2
IPI00011998	BREAST CANCER ANTI-ESTROGEN RESISTANCE 1.	......yD.P...	2
IPI00031407	neural precursor cell expressed, developmentally down-regulated 9	......yD.P...	2
IPI00011998	BREAST CANCER ANTI-ESTROGEN RESISTANCE 1.	......yD.P...	2
IPI00011998	BREAST CANCER ANTI-ESTROGEN RESISTANCE 1.	......yD.P...	2
IPI00641339	CDNA FLJ55515, HIGHLY SIMILAR TO BREAST CANCER ANTI-ESTROGEN RESISTANCE PROTEIN 1.	......yD.P...	2
IPI00031407	neural precursor cell expressed, developmentally down-regulated 9	......yD.P...	2
IPI00031407	neural precursor cell expressed, developmentally down-regulated 9	......yD.P...	2
IPI00011998	BREAST CANCER ANTI-ESTROGEN RESISTANCE 1.	......yD.P...	2
IPI00014454	Ras and Rab interactor 1	......yD.P...	2
IPI00017578	Src homology 2 domain containing adaptor protein B	......yD.P...	2
IPI00017578	Src homology 2 domain containing adaptor protein B	......yD.P...	2
IPI00017578	Src homology 2 domain containing adaptor protein B	......yD.P...	2
IPI00031068	GRB2-associated binding protein 1	......yD.P...	2
IPI00017578	Src homology 2 domain containing adaptor protein B	......yD.P...	2
IPI00257508	dihydropyrimidinase-like 2	......yD.P...	2
IPI00419836	discoidin, CUB and LCCL domain containing 2	......yD.P...	2
IPI00337612	DISCOIDIN, CUB AND LCCL DOMAIN-CONTAINING PROTEIN 1.	......yD.P...	2
IPI00419836	discoidin, CUB and LCCL domain containing 2	......yD.P...	2
IPI00027721	platelet-derived growth factor receptor, alpha polypeptide	......yD.P...	2
IPI00027834	heterogeneous nuclear ribonucleoprotein L	......yD.P...	2
IPI00012885	PTK2 protein tyrosine kinase 2	...D..y......	3
IPI00464978	insulin receptor substrate 2	...D..y......	3
IPI00464978	insulin receptor substrate 2	...D..y......	3
IPI00186826	EPHRIN RECEPTOR.	...D..y......	3
IPI00012885	PTK2 protein tyrosine kinase 2	...D..y......	3
IPI00001654	ISOFORM 3 OF PERICENTRIOLAR MATERIAL 1 PROTEIN.	...D..y......	3
IPI00028931	desmoglein 2	...D..y......	3
IPI00045423	ISOFORM 7 OF PARTITIONING DEFECTIVE 3 HOMOLOG.	...D..y......	3
IPI00744706	CDNA FLJ61399, HIGHLY SIMILAR TO SPECTRIN ALPHA CHAIN, BRAIN.	...D..y......	3
IPI00064607	multiple EGF-like-domains 10	...D..y......	3
IPI00013988	Rho GTPase activating protein 5	...D..y......	3
IPI00411452	PUTATIVE UNCHARACTERIZED PROTEIN DOCK11.	...D..y......	3
IPI00029273	met proto-oncogene (hepatocyte growth factor receptor)	...D..y......	3
IPI00029273	met proto-oncogene (hepatocyte growth factor receptor)	...D..y......	3
IPI00216592	heterogeneous nuclear ribonucleoprotein C (C1/C2)	...D..y......	3
IPI00382946	CCDC123 PROTEIN (FRAGMENT).	...D..y......	3
IPI00061178	RNA binding motif protein, X-linked-like 1	...D..y......	3
IPI00216219	tight junction protein 1 (zona occludens 1)	...D..y......	3
IPI00383423	coiled-coil domain containing 50	...D..y......	3
IPI00014344	dual-specificity tyrosine-(Y)-phosphorylation regulated kinase 1A	...D..y......	3
IPI00021076	plakophilin 4	...D..y......	3
IPI00005264	plakophilin 2	...D..y......	3
IPI00008868	microtubule-associated protein 1B	...D..y......	3
IPI00012007	adenosylhomocysteinase	...D..y......	3
IPI00008868	microtubule-associated protein 1B	...D..y......	3
IPI00008868	microtubule-associated protein 1B	...D..y......	3
IPI00015180	shroom family member 2	...D..y......	3
IPI00296784	MARVEL domain containing 2	...D..y......	3
IPI00061178	RNA binding motif protein, X-linked-like 1	...D..y......	3
IPI00014197	CDV3 homolog (mouse)	...D..y......	3
IPI00013881	heterogeneous nuclear ribonucleoprotein H1 (H)	...D..y......	3
IPI00784156	adaptor-related protein complex 2, beta 1 subunit	...D..y......	3
IPI00013933	desmoplakin	...D..y......	3
IPI00410034	solute carrier family 38, member 2	...D..y......	3
IPI00027667	cholecystokinin B receptor	...D..y......	3
IPI00182469	catenin (cadherin-associated protein), delta 1	...D..y......	3
IPI00023704	LIM domain containing preferred translocation partner in lipoma	...D..y......	3
IPI00170865	membrane associated guanylate kinase, WW and PDZ domain containing 3	...D..y......	3
IPI00376306	ISOFORM 2 OF MEMBRANE-ASSOCIATED GUANYLATE KINASE, WW AND PDZ DOMAIN- CONTAINING PRO	...D..y......	3
IPI00165946	membrane associated guanylate kinase, WW and PDZ domain containing 1	...D..y......	3
IPI00031068	GRB2-associated binding protein 1	...D..y......	3
IPI00045423	ISOFORM 7 OF PARTITIONING DEFECTIVE 3 HOMOLOG.	...D..y......	3

IPI00029769	hemopoietic cell kinase	...D..y......	3
IPI00007750	tubulin, alpha 4a	...D..y......	3
IPI00181905	actin filament associated protein 1-like 2	...D..y......	3
IPI00220032	ISOFORM 2 OF CATENIN DELTA-2.	...D..y......	3
IPI00013981	v-yes-1 Yamaguchi sarcoma viral oncogene homolog 1	...D..y......	3
IPI00021326	SHC (Src homology 2 domain containing) transforming protein 1	...D..y......	3
IPI00217059	coiled-coil domain containing 50	...D..y......	3
IPI00438229	tripartite motif-containing 28	...D..y......	3
IPI00011736	phosphoinositide-3-kinase, regulatory subunit 2 (beta)	...D..y......	3
IPI00328218	ISOFORM 2 OF LIPOLYSIS-STIMULATED LIPOPROTEIN RECEPTOR.	...D..y......	3
IPI00290337	epidermal growth factor receptor pathway substrate 8	...D..y......	3
IPI00328218	ISOFORM 2 OF LIPOLYSIS-STIMULATED LIPOPROTEIN RECEPTOR.	...D..y......	3
IPI00412771	CD2-associated protein	...D..y......	3
IPI00384861	G protein-coupled receptor kinase interacting ArfGAP 1	...D..y......	3
IPI00012885	PTK2 protein tyrosine kinase 2	...D..y......	3
IPI00012885	PTK2 protein tyrosine kinase 2	...D..y......	3
IPI00012885	PTK2 protein tyrosine kinase 2	...D..y......	3
IPI00029702	PTK2B protein tyrosine kinase 2 beta	...D..y......	3
IPI00019471	insulin receptor substrate 1	...D..y......	3
IPI00337612	DISCOIDIN, CUB AND LCCL DOMAIN-CONTAINING PROTEIN 1.	...D..y......	3
IPI00464978	insulin receptor substrate 2	...D..y......	3
IPI00419933	pleckstrin homology domain containing, family A member 7	...D..y......	3
IPI00419933	pleckstrin homology domain containing, family A member 7	...D..y......	3
IPI00021290	ATP citrate lyase	...D..y......	3
IPI00464978	insulin receptor substrate 2	...D..y......	3
IPI00005904	DEAD (Asp-Glu-Ala-Asp) box polypeptide 20	...D..y......	3
IPI00186826	EPHRIN RECEPTOR.	...D..y......	3
IPI00008315	EPH receptor B1	...D..y......	3
IPI00021275	ISOFORM 1 OF EPHRIN TYPE-B RECEPTOR 2.	...D..y......	3
IPI00294250	EPH receptor A1	...D..y......	3
IPI00289329	EPH receptor B3	...D..y......	3
IPI00291175	vinculin	...D..y......	3
IPI00030887	TYRO3 protein tyrosine kinase	...D..y......	3
IPI00395663	ankyrin repeat and sterile alpha motif domain containing 1A	...D..y......	3
IPI00182469	catenin (cadherin-associated protein), delta 1	...D..y......	3
IPI00307545	tensin 1	...D..y......	3
IPI00028931	desmoglein 2	...D..y......	3
IPI00025803	insulin receptor	...E..y......	4
IPI00025803	insulin receptor	...E..y......	4
IPI00026689	cell division cycle 2, G1 to S and G2 to M	...E..y......	4
IPI00023503	cyclin-dependent kinase 3	...E..y......	4
IPI00019025	par-6 partitioning defective 6 homolog beta (C. elegans)	...E..y......	4
IPI00028065	NCK adaptor protein 1	...E..y......	4
IPI00797763	cingulin	...E..y......	4
IPI00008868	microtubule-associated protein 1B	...E..y......	4
IPI00438286	ERBB2IP PROTEIN.	...E..y......	4
IPI00334715	ISOFORM 1 OF GLUCOCORTICOID RECEPTOR DNA-BINDING FACTOR 1.	...E..y......	4
IPI00021076	plakophilin 4	...E..y......	4
IPI00045423	ISOFORM 7 OF PARTITIONING DEFECTIVE 3 HOMOLOG.	...E..y......	4
IPI00220030	paxillin	...E..y......	4
IPI00025803	insulin receptor	...E..y......	4
IPI00025803	insulin receptor	...E..y......	4
IPI00027422	integrin, beta 4	...E..y......	4
IPI00025803	insulin receptor	...E..y......	4
IPI00004901	CDNA FLJ20242 FIS, CLONE COLF6369.	...E..y......	4
IPI00004901	CDNA FLJ20242 FIS, CLONE COLF6369.	...E..y......	4
IPI00015180	shroom family member 2	...E..y......	4
IPI00298285	v-erb-b2 erythroblastic leukemia viral oncogene homolog 3 (avian)	...E..y......	4
IPI00298285	v-erb-b2 erythroblastic leukemia viral oncogene homolog 3 (avian)	...E..y......	4
IPI00183445	latrophilin 1	...E..y......	4
IPI00017562	latrophilin 2	...E..y......	4
IPI00216219	tight junction protein 1 (zona occludens 1)	...E..y......	4
IPI00072377	similar to SET translocation	...E..y......	4
IPI00023530	cyclin-dependent kinase 5	...E..y......	4
IPI00026689	cell division cycle 2, G1 to S and G2 to M	...E..y......	4
IPI00031681	cyclin-dependent kinase 2	...E..y......	4
IPI00219299	talin 2	...E..y......	4
IPI00171134	ISOFORM 1 OF GIRDIN.	...E..y......	4
IPI00011913	heterogeneous nuclear ribonucleoprotein A0	...E..y......	4
IPI00464978	insulin receptor substrate 2	...E..y......	4
IPI00470838	ISOFORM 1 OF DENN DOMAIN-CONTAINING PROTEIN 2C.	...E..y......	4
IPI00169383	phosphoglycerate kinase 1	...E..y......	4
IPI00021439	actin, beta	...E..y......	4
IPI00006176	hepatocyte growth factor-regulated tyrosine kinase substrate	...E..y......	4
IPI00001466	echinoderm microtubule associated protein like 4	...E..y......	4
IPI00219217	lactate dehydrogenase B	...E..y......	4
IPI00174976	membrane protein, palmitoylated 5 (MAGUK p55 subfamily member 5)	...E..y......	4
IPI00289334	filamin B, beta	...E..y......	4
IPI00179053	breast cancer anti-estrogen resistance 3	...E..y......	4
IPI00022353	tyrosine kinase 2	...E..y......	4
IPI00418545	Rho guanine nucleotide exchange factor (GEF) 5	...E..y......	4
IPI00186990	GRB2-associated binding protein 2	...E..y......	4
IPI00302925	59 KDA PROTEIN.	...E..y......	4
IPI00018522	protein arginine methyltransferase 1	...E..y......	4
IPI00099883	G protein-coupled receptor, family C, group 5, member C	...E..y......	4
IPI00099883	G protein-coupled receptor, family C, group 5, member C	...E..y......	4
IPI00024307	ephrin-B1	...E..y......	4
IPI00001762	oligophrenin 1	...E..y......	4
IPI00221089	ribosomal protein S13	...E..y......	4

IPI00737545	NKF3 kinase family member	...E..y......	4
IPI00012885	PTK2 protein tyrosine kinase 2	...E..y......	4
IPI00329327	ISOFORM 2 OF EXTRACELLULAR MATRIX PROTEIN FRAS1.	...E..y......	4
IPI00016670	chromosome 11 open reading frame 59	...E..y......	4
IPI00043622	ISOFORM 1 OF FERM DOMAIN-CONTAINING PROTEIN 6.	...E..y......	4
IPI00023503	cyclin-dependent kinase 3	...E..y......	4
IPI00023503	cyclin-dependent kinase 3	...E..y......	4
IPI00029601	cortactin	...E..y......	4
IPI00171499	family with sequence similarity 59, member A	...E..y......	4
IPI00395663	ankyrin repeat and sterile alpha motif domain containing 1A	...E..y......	4
IPI00376221	erythrocyte membrane protein band 4.1 like 5	...E..y......	4
IPI00014873	cyclin-dependent kinase 10	...E..y......	4
IPI00152881	shroom family member 3	...E..y......	4
IPI00220032	ISOFORM 2 OF CATENIN DELTA-2.	...E..y......	4
IPI00221316	ISOFORM C OF PROTEIN SPROUTY HOMOLOG 4.	...E..y......	4
IPI00183046	protein tyrosine phosphatase, non-receptor type 6	...E..y......	4
IPI00298860	lactotransferrin	...E..y......	4
IPI00301561	thyroid hormone receptor interactor 6	...E..y......	4
IPI00216008	ISOFORM LONG OF GLUCOSE-6-PHOSPHATE 1-DEHYDROGENASE.	...E..y......	4
IPI00186826	EPHRIN RECEPTOR.	...E..y......	4
IPI00021267	EPH receptor A2	...E..y......	4
IPI00216991	G protein-coupled receptor kinase interacting ArfGAP 2	...E..y......	4
IPI00029702	PTK2B protein tyrosine kinase 2 beta	...E..y......	4
IPI00005142	fibroblast growth factor receptor 1	...E..y......	4
IPI00005142	fibroblast growth factor receptor 1	...E..y......	4
IPI00005142	fibroblast growth factor receptor 1	...E..y......	4
IPI00016645	EPH receptor A7	...E..y......	4
IPI00654623	SIMILAR TO TENSIN 3.	...E..y......	4
IPI00171499	family with sequence similarity 59, member A	...E..y......	4
IPI00419836	discoidin, CUB and LCCL domain containing 2	...E..y......	4
IPI00016736	phospholipase C, gamma 1	...E..y......	4
IPI00021267	EPH receptor A2	...E..y......	4
IPI00016645	EPH receptor A7	...E..y......	4
IPI00162743	suppression of tumorigenicity 5	...E..y......	4
IPI00008290	EPH receptor A5	...E..y......	4
IPI00015756	ISOFORM 1 OF RECEPTOR-TYPE TYROSINE-PROTEIN PHOSPHATASE KAPPA.	...E..y......	4
IPI00644865	FERM domain containing 4A	...E..y......	4
IPI00300384	v-erb-b2 erythroblastic leukemia viral oncogene homolog 2, neuro/glioblastoma derived oncogene homolog (avian)	...E..y......	4
IPI00016932	inositol polyphosphate phosphatase-like 1	...E..y......	4
IPI00013983	ret proto-oncogene	...E..y......	4
IPI00013983	ret proto-oncogene	...E..y......	4
IPI00140420	staphylococcal nuclease and tudor domain containing 1	...E..y......	4
IPI00218570	phosphoglycerate mutase 2 (muscle)	...E..y......	4
IPI00004901	CDNA FLJ20242 FIS, CLONE COLF6369.	...E..y......	4
IPI00654623	SIMILAR TO TENSIN 3.	......y...R..	5
IPI00005741	mitogen-activated protein kinase 13	......y...R..	5
IPI00002857	mitogen-activated protein kinase 14	......y...R..	5
IPI00003479	mitogen-activated protein kinase 1	......y...R..	5
IPI00003145	mitogen-activated protein kinase 8	......y...R..	5
IPI00296283	mitogen-activated protein kinase 12	......y...R..	5
IPI00003479	mitogen-activated protein kinase 1	......y...R..	5
IPI00018195	mitogen-activated protein kinase 3	......y...R..	5
IPI00301987	missing oocyte, meiosis regulator, homolog (Drosophila)	......y...R..	5
IPI00149048	mitogen-activated protein kinase 7	......y...R..	5
IPI00059185	chromosome 11 open reading frame 52	......y...R..	5
IPI00021076	plakophilin 4	......y...R..	5
IPI00024320	RNA binding motif (RNP1, RRM) protein 3	......y...R..	5
IPI00024320	RNA binding motif (RNP1, RRM) protein 3	......y...R..	5
IPI00298285	v-erb-b2 erythroblastic leukemia viral oncogene homolog 3 (avian)	......y...R..	5
IPI00216219	tight junction protein 1 (zona occludens 1)	......y...R..	5
IPI00022434	PUTATIVE UNCHARACTERIZED PROTEIN ALB.	......y...R..	5
IPI00746301	cyclin-dependent kinase-like 5	......y...R..	5
IPI00003985	BCS1-like (yeast)	......y...R..	5
IPI00005741	mitogen-activated protein kinase 13	......y...R..	5
IPI00002857	mitogen-activated protein kinase 14	......y...R..	5
IPI00296283	mitogen-activated protein kinase 12	......y...R..	5
IPI00003145	mitogen-activated protein kinase 8	......y...R..	5
IPI00024673	mitogen-activated protein kinase 9	......y...R..	5
IPI00003479	mitogen-activated protein kinase 1	......y...R..	5
IPI00029769	hemopoietic cell kinase	......y...R..	5
IPI00298625	v-yes-1 Yamaguchi sarcoma viral related oncogene homolog	......y...R..	5
IPI00018195	mitogen-activated protein kinase 3	......y...R..	5
IPI00028570	glycogen synthase kinase 3 beta	......y...R..	5
IPI00028570	glycogen synthase kinase 3 beta	......y...R..	5
IPI00013981	v-yes-1 Yamaguchi sarcoma viral oncogene homolog 1	......y...R..	5
IPI00059964	anterior pharynx defective 1 homolog A (C. elegans)	......y...R..	5
IPI00031068	GRB2-associated binding protein 1	......y...R..	5
IPI00298994	talin 1	......y...R..	5
IPI00006482	ATPase, Na+/K+ transporting, alpha 1 polypeptide	......y...R..	5
IPI00022558	myelin protein zero-like 1	......y...R..	5
IPI00000352	dual-specificity tyrosine-(Y)-phosphorylation regulated kinase 1B	......y...R..	5
IPI00219299	talin 2	......y...R..	5
IPI00220032	ISOFORM 2 OF CATENIN DELTA-2.	......y...R..	5
IPI00182469	catenin (cadherin-associated protein), delta 1	......y...R..	5
IPI00022521	dual-specificity tyrosine-(Y)-phosphorylation regulated kinase 2	......y...R..	5
IPI00332841	Rap guanine nucleotide exchange factor (GEF) 1	......y...R..	5
IPI00017578	Src homology 2 domain containing adaptor protein B	......y...R..	5
IPI00099522	homeodomain interacting protein kinase 3	......y...R..	5
IPI00099522	homeodomain interacting protein kinase 3	......y...R..	5

IPI00215949	similar to homeodomain interacting protein kinase 2	......y...R..	5
IPI00215949	similar to homeodomain interacting protein kinase 2	......y...R..	5
IPI00419373	heterogeneous nuclear ribonucleoprotein A3	......y...R..	5
IPI00103018	PUTATIVE UNCHARACTERIZED PROTEIN KIAA1217.	......y...R..	5
IPI00012752	growth factor receptor-bound protein 10	......y...R..	5
IPI00001477	discoidin domain receptor tyrosine kinase 1	......y...R..	5
IPI00453473	histone cluster 2, H4b	......y...R..	5
IPI00382749	RIBOSOMAL PROTEIN L15.	......y...R..	5
IPI00464978	insulin receptor substrate 2	......y...R..	5
IPI00470360	kin of IRRE like (Drosophila)	......y...R..	5
IPI00215948	catenin (cadherin-associated protein), alpha 1, 102kDa	......y...R..	5
IPI00024312	FYVE, RhoGEF and PH domain containing 1	......y...R..	5
IPI00470360	kin of IRRE like (Drosophila)	......y...R..	5
IPI00005264	plakophilin 2	......y...R..	5
IPI00024067	clathrin, heavy chain (Hc)	......y...R..	5
IPI00337612	DISCOIDIN, CUB AND LCCL DOMAIN-CONTAINING PROTEIN 1.	......y...R..	5
IPI00298994	talin 1	......y...R..	5
IPI00296992	AXL receptor tyrosine kinase	......y...R..	5
IPI00296992	AXL receptor tyrosine kinase	......y...R..	5
IPI00166680	misshapen-like kinase 1 (zebrafish)	......y...R..	5
IPI00419100	FYVE, RhoGEF and PH domain containing 6	......y...R..	5
IPI00029756	c-mer proto-oncogene tyrosine kinase	......y...R..	5
IPI00219757	glutathione S-transferase pi 1	......y...R..	5
IPI00149048	mitogen-activated protein kinase 7	......y...R..	5
IPI00013721	similar to hCG1820375	......y...R..	5
IPI00478892	leucine-rich repeats and immunoglobulin-like domains 2	......y...R..	5
IPI00021428	actin, alpha 1, skeletal muscle	......y...R..	5
IPI00219798	roundabout, axon guidance receptor, homolog 1 (Drosophila)	......y...R..	5
IPI00021076	plakophilin 4	......yE.....	6
IPI00029601	cortactin	......yE.....	6
IPI00029601	cortactin	......yE.....	6
IPI00021267	EPH receptor A2	......yE.....	6
IPI00008318	EPH receptor A4	......yE.....	6
IPI00216219	tight junction protein 1 (zona occludens 1)	......yE.....	6
IPI00644231	cytoplasmic FMR1 interacting protein 1	......yE.....	6
IPI00021076	plakophilin 4	......yE.....	6
IPI00008438	ribosomal protein S10	......yE.....	6
IPI00025803	insulin receptor	......yE.....	6
IPI00025803	insulin receptor	......yE.....	6
IPI00025803	insulin receptor	......yE.....	6
IPI00025803	insulin receptor	......yE.....	6
IPI00025803	insulin receptor	......yE.....	6
IPI00301561	thyroid hormone receptor interactor 6	......yE.....	6
IPI00175416	phospholipase C, eta 1	......yE.....	6
IPI00025803	insulin receptor	......yE.....	6
IPI00012011	cofilin 1 (non-muscle)	......yE.....	6
IPI00383423	coiled-coil domain containing 50	......yE.....	6
IPI00478817	Rho guanine nucleotide exchange factor (GEF) 10-like	......yE.....	6
IPI00021439	actin, beta	......yE.....	6
IPI00031407	neural precursor cell expressed, developmentally down-regulated 9	......yE.....	6
IPI00008868	microtubule-associated protein 1B	......yE.....	6
IPI00008868	microtubule-associated protein 1B	......yE.....	6
IPI00167198	ISOFORM 2 OF U4/U6 SMALL NUCLEAR RIBONUCLEOPROTEIN PRP31.	......yE.....	6
IPI00182469	catenin (cadherin-associated protein), delta 1	......yE.....	6
IPI00107633	SH3 AND MULTIPLE ANKYRIN REPEAT DOMAINS 2 ISOFORM 2.	......yE.....	6
IPI00216969	c-abl oncogene 1, receptor tyrosine kinase	......yE.....	6
IPI00023704	LIM domain containing preferred translocation partner in lipoma	......yE.....	6
IPI00005126	ephrin-B2	......yE.....	6
IPI00107698	fibroblast growth factor receptor substrate 2	......yE.....	6
IPI00328737	zinc finger protein 598	......yE.....	6
IPI00024307	ephrin-B1	......yE.....	6
IPI00217872	ISOFORM 2 OF PHOSPHOGLUCOMUTASE-1.	......yE.....	6
IPI00021076	plakophilin 4	......yE.....	6
IPI00302690	actin-related protein 10 homolog (S. cerevisiae)	......yE.....	6
IPI00029263	fer (fps/fes related) tyrosine kinase	......yE.....	6
IPI00186990	GRB2-associated binding protein 2	......yE.....	6
IPI00021076	plakophilin 4	......yE.....	6
IPI00029601	cortactin	......yE.....	6
IPI00216171	enolase 2 (gamma, neuronal)	......yE.....	6
IPI00328587	ENOLASE.	......yE.....	6
IPI00003431	mitogen-activated protein kinase 6	......yE.....	6
IPI00016736	phospholipase C, gamma 1	......yE.....	6
IPI00217223	phosphoribosylaminoimidazole carboxylase, phosphoribosylaminoimidazole succinocarboxamide synthetase	......yE.....	6
IPI00010740	splicing factor proline/glutamine-rich (polypyrimidine tract binding protein associated)	......yE.....	6
IPI00414123	collapsin response mediator protein 1	......yE.....	6
IPI00022959	poliovirus receptor-related 3	......yE.....	6
IPI00216008	ISOFORM LONG OF GLUCOSE-6-PHOSPHATE 1-DEHYDROGENASE.	......yE.....	6
IPI00298347	protein tyrosine phosphatase, non-receptor type 11	......yE.....	6
IPI00021267	EPH receptor A2	......yE.....	6
IPI00186826	EPHRIN RECEPTOR.	......yE.....	6
IPI00021267	EPH receptor A2	......yE.....	6
IPI00186826	EPHRIN RECEPTOR.	......yE.....	6
IPI00008318	EPH receptor A4	......yE.....	6
IPI00008315	EPH receptor B1	......yE.....	6
IPI00008318	EPH receptor A4	......yE.....	6
IPI00016645	EPH receptor A7	......yE.....	6
IPI00291175	vinculin	......yE.....	6
IPI00470360	kin of IRRE like (Drosophila)	......yE.....	6
IPI00296913	nudix (nucleoside diphosphate linked moiety X)-type motif 5	......yE.....	6

IPI00031030	amyloid beta (A4) precursor-like protein 2	......yE.....	6
IPI00006608	amyloid beta (A4) precursor protein	......yE.....	6
IPI00643785	MYELOID/LYMPHOID OR MIXED-LINEAGE LEUKEMIA.	......yE.....	6
IPI00419100	FYVE, RhoGEF and PH domain containing 6	......yE.....	6
IPI00016736	phospholipase C, gamma 1	......yE.....	6
IPI00013983	ret proto-oncogene	......yE.....	6
IPI00013983	ret proto-oncogene	......yE.....	6
IPI00329801	annexin A5	......yE.....	6
IPI00023461	ISOFORM 4 OF AFADIN.	......yE.....	6
IPI00293126	tubulin folding cofactor B	......yE.....	6
IPI00029273	met proto-oncogene (hepatocyte growth factor receptor)	.....Dy......	7
IPI00018335	fms-related tyrosine kinase 1 (vascular endothelial growth factor/vascular permeability factor receptor)	.....Dy......	7
IPI00334715	ISOFORM 1 OF GLUCOCORTICOID RECEPTOR DNA-BINDING FACTOR 1.	.....Dy......	7
IPI00021076	plakophilin 4	.....Dy......	7
IPI00018274	epidermal growth factor receptor (erythroblastic leukemia viral (v-erb-b) oncogene homolog, avian)	.....Dy......	7
IPI00298285	v-erb-b2 erythroblastic leukemia viral oncogene homolog 3 (avian)	.....Dy......	7
IPI00396435	DEAH (Asp-Glu-Ala-His) box polypeptide 15	.....Dy......	7
IPI00029515	pleckstrin homology domain containing, family A member 5	.....Dy......	7
IPI00014344	dual-specificity tyrosine-(Y)-phosphorylation regulated kinase 1A	.....Dy......	7
IPI00844578	DEAH (Asp-Glu-Ala-His) box polypeptide 9	.....Dy......	7
IPI00005264	plakophilin 2	.....Dy......	7
IPI00829652	RNA binding motif protein 16	.....Dy......	7
IPI00026952	plakophilin 3	.....Dy......	7
IPI00029601	cortactin	.....Dy......	7
IPI00017578	Src homology 2 domain containing adaptor protein B	.....Dy......	7
IPI00031461	CDNA FLJ60299, HIGHLY SIMILAR TO RAB GDP DISSOCIATION INHIBITOR BETA.	.....Dy......	7
IPI00431025	abl-interactor 1	.....Dy......	7
IPI00442210	ISOFORM 1 OF ABL INTERACTOR 2.	.....Dy......	7
IPI00008530	ribosomal protein, large, P0	.....Dy......	7
IPI00023704	LIM domain containing preferred translocation partner in lipoma	.....Dy......	7
IPI00017578	Src homology 2 domain containing adaptor protein B	.....Dy......	7
IPI00185919	La ribonucleoprotein domain family, member 1	.....Dy......	7
IPI00027705	primase, DNA, polypeptide 2 (58kDa)	.....Dy......	7
IPI00012885	PTK2 protein tyrosine kinase 2	.....Dy......	7
IPI00012885	PTK2 protein tyrosine kinase 2	.....Dy......	7
IPI00015287	docking protein 1, 62kDa (downstream of tyrosine kinase 1)	.....Dy......	7
IPI00022228	high density lipoprotein binding protein	.....Dy......	7
IPI00455851	KIAA1688 protein	.....Dy......	7
IPI00298347	protein tyrosine phosphatase, non-receptor type 11	.....Dy......	7
IPI00464978	insulin receptor substrate 2	.....Dy......	7
IPI00027174	fibroblast growth factor receptor 3	.....Dy......	7
IPI00027174	fibroblast growth factor receptor 3	.....Dy......	7
IPI00054004	HYPOTHETICAL PROTEIN LOC643896.	.....Dy......	7
IPI00005142	fibroblast growth factor receptor 1	.....Dy......	7
IPI00005142	fibroblast growth factor receptor 1	.....Dy......	7
IPI00010680	fibroblast growth factor receptor 2	.....Dy......	7
IPI00010680	fibroblast growth factor receptor 2	.....Dy......	7
IPI00031068	GRB2-associated binding protein 1	.....Dy......	7
IPI00023343	discs, large homolog 3 (Drosophila)	.....Dy......	7
IPI00464978	insulin receptor substrate 2	.....Dy......	7
IPI00296992	AXL receptor tyrosine kinase	.....Dy......	7
IPI00296992	AXL receptor tyrosine kinase	.....Dy......	7
IPI00470360	kin of IRRE like (Drosophila)	.....Dy......	7
IPI00029756	c-mer proto-oncogene tyrosine kinase	.....Dy......	7
IPI00029756	c-mer proto-oncogene tyrosine kinase	.....Dy......	7
IPI00221067	ISOFORM 2 OF RECEPTOR-TYPE TYROSINE-PROTEIN PHOSPHATASE ALPHA.	.....Dy......	7
IPI00001477	discoidin domain receptor tyrosine kinase 1	.....Dy......	7
IPI00307545	tensin 1	.....Dy......	7
IPI00013396	small nuclear ribonucleoprotein polypeptide C	.....Dy......	7
IPI00180143	CDC-like kinase 4	.....Dy......	7
IPI00004500	Nedd4 binding protein 3	.....Dy......	7
IPI00292856	Cas-Br-M (murine) ecotropic retroviral transforming sequence b	.....Dy......	7
IPI00374301	HYPOTHETICAL PROTEIN.	.....Dy......	7
IPI00002211	ISOFORM 2 OF SEMAPHORIN-6A.	.....Dy......	7
IPI00032003	emerin	.....Dy......	7
IPI00464978	insulin receptor substrate 2	.....Dy......	7
