# Supplementary material for: Tyrosine Phosphorylation Profiling in FGF-2 Stimulated Human Embryonic Stem Cells
Source: PLoS One. 2011 Mar 17;6(3):e17538. doi: 10.1371/journal.pone.0017538 (PMC3060089; doi:10.1371/journal.pone.0017538)
Supplement: Table S3 — Motif assignment for phosphopeptides from the cumulative dataset. (PDF) [file pone.0017538.s006.pdf]

IPI accession no.	Protein	Phosphopeptide	Phosphosites	* Fold Change of phosphorylation post FGF-2 stimulation		
				0 min	5 min	15 min
IPI00431025	ABI1 Isoform 1 of Abl interactor 1	TLEPVKPPTVPNDpYMTSPAR	Y213	0	0.68	0.13
IPI00216969	ABL1 Isoform IA of Proto-oncogene tyrosine-protein kinase ABL1	INTASDGKLpYVSSESR	Y185	0	-0.11	-0.07
IPI00216969	ABL1 Isoform IA of Proto-oncogene tyrosine-protein kinase ABL1	LMTGDTpYTAHAGAK	Y393	0	-0.11	-0.17
IPI00216969	ABL1 Isoform IA of Proto-oncogene tyrosine-protein kinase ABL1	LGGGQYGEVpYEGVWKK	Y257	0	0.47	0.26
IPI00013508	ACTN1 Alpha-actinin-1	AIMTpYVSSFYHAFSGAQK	Y241	0	-0.12	-0.20
IPI00181905	AFAP1L2 Isoform 1 of Actin filament-associated protein 1-like 2	SKVAQQPLSLVGCEVVPDPSPDHLpYSFR	Y413	0	-0.05	0.54
IPI00395663	ANKS1A Ankyrin repeat and SAM domain-containing protein 1A	EEDEHPpYELLLTAETK	Y455	0	1.32	1.25
IPI00217418	ARHGAP12 Isoform 2 of Rho GTPase-activating protein 12	ATTPPNQGRPDpSPVpYANLQELK	Y243	0	-0.49	-0.51
IPI00646885	ARHGAP5 Rho GTPase activating protein 5 isoform a	VPLAHPEDMDPSDNpYAEPIDTIFK	Y1091	0	0.68	0.77
IPI00013988	ARHGAP5 Rho GTPase-activating protein 5	GYSDEIpYVVPDDSQNR	Y1109	0	0.31	0.37
IPI00641339	BCAR1 93 kDa protein	AQQGLpYQVPGPSPQFQSPPAK	Y212	0	0.52	0.39
IPI00641339	BCAR1 93 kDa protein	DVPDGPLLREETpYDVPPAFAK	Y411	0	0.82	0.54
IPI00641339	BCAR1 93 kDa protein	GLLPSQpYGQEVYDTPPMAVK	Y346	0	0.77	0.46
IPI00641339	BCAR1 93 kDa protein	GPNGRDPLLEVpYDVPPSVEK	Y371	0	0.33	0.19
IPI00641339	BCAR1 93 kDa protein	HLLAPGPQDIpYDVPPVR	Y333	0	0.07	0.09
IPI00641339	BCAR1 93 kDa protein	RPGPGTLpYDVPR	Y471	0	0.47	0.08
IPI00641339	BCAR1 93 kDa protein	VLPPEVADGGVVDSGVpYAVPPPAER	Y494	0	0.75	0.83
IPI00004497	BCR Isoform 1 of Breakpoint cluster region protein	GHGQPGADAEKPFpYVNVEFHHER	Y177	0	1.19	1.09
IPI00059185	C11orf52 Uncharacterized protein C11orf52	HVHLENATEpYATLR	Y103	0	1.53	0.19
IPI00016670	C11orf59 UPF0404 protein C11orf59	ALNGAEPNpYHSLPSAR	Y40	0	1.11	0.91
IPI00007067	C9orf19 Golgi-associated plant pathogenesis-related protein 1	EAQQpYSEALASTR	Y42	0	-0.50	0.05
IPI00027269	CBL E3 ubiquitin-protein ligase CBL	IKPSSSANAIpYSLAARPLPVPK	Y674	0	0.94	0.56
IPI00061178	CCBL2 kynurenine aminotransferase III isoform 3	DVpYLSPRDDGYSTK	Y222	0	-0.35	-0.56
IPI00382946	CCDC123 CCDC123 protein (Fragment)	GGHSDDLpYAVPHR	Y157	0	0.00	0.06
IPI00219852	CD46 Isoform B of Membrane cofactor protein precursor	ADGGAEpYATYQTK	Y384	0	2.74	2.14
IPI00026689	CDC2 Putative uncharacterized protein DKFZp686L20222	IEKIGEGpTpYGVVYK	T14, Y15	0	-0.16	-0.09
IPI00026689	CDC2 Putative uncharacterized protein DKFZp686L20222	IGEGTpYGVVYK	Y15	0	-0.45	-0.14
IPI00026689	CDC2 Putative uncharacterized protein DKFZp686L20222	IGEGTYGVVpYK	Y19	0	0.26	0.11
IPI00031681	CDK2 Cell division protein kinase 2	VEKIGEGpTpYGVVYK	T14, Y15	0	-0.39	-0.77
IPI00031681	CDK2 Cell division protein kinase 2	VEKIGEGpTYGVVpYK	T14, Y19	0	-0.37	-0.70
IPI00031681	CDK2 Cell division protein kinase 2	VEKIGEGTpYGVVYK	Y15	0	-0.56	-0.34
IPI00023530	CDK5 Cell division protein kinase 5	IGEGTpYGTVFK	Y15	0	1.33	1.57
IPI00746301	CDKL5 Uncharacterized protein CDKL5	NLSEGNNANYTEpYVATR	Y171	0	0.37	0.43
IPI00014197	CDV3 Isoform 1 of Protein CDV3 homolog	KTPQGPPEIpYSDTQFPSLQSTAK	Y190	0	1.98	1.50
IPI00797763	CGN Cingulin	GANDQGASGALSSDLELPENPpYSQVK	Y105	0	0.29	0.44
IPI00141318	CKAP4 Isoform 1 of Cytoskeleton-associated protein 4	STLQTMESDIpYTEVR	Y322	0	-0.05	-0.16
IPI00011084	CLDN6 Claudin-6	GPSEpYPTKNpYV	Y214, Y219	0	0.28	0.49
IPI00011084	CLDN6 Claudin-6	GPSEYPTKNpYV	Y219	0	0.90	0.79
IPI00024067	CLTC Isoform 1 of Clathrin heavy chain 1	ALEHFTDLpYDIKR	Y634	0	-0.44	-0.59
IPI00295857	COPA Coatomer subunit alpha	GHpYNNVSCAVFHPR	Y249	0	0.17	0.19
IPI00414123	CRMP1 Dihydropyrimidinase-related protein 1	GMpYDGPVYEVPATPK	Y499	0	0.31	0.28
IPI00215948	CTNNA1 Isoform 1 of Catenin alpha-1	ANRDLIpYK	Y245	0	0.45	0.96
IPI00215948	CTNNA1 Isoform 1 of Catenin alpha-1	LVpYDGIR	Y619	0	-0.17	-0.35
IPI00215948	CTNNA1 Isoform 1 of Catenin alpha-1	NAGNEQDLGIQpYK	Y177	0	-0.07	0.07
IPI00219725	CTNND1 Isoform 2AB of Catenin delta-1	HYEDGYPGGSDNpYGSLSR	Y174	0	1.04	1.21
IPI00219725	CTNND1 Isoform 2AB of Catenin delta-1	LNGPQDHSHLLpYSTIPR	Y42	0	0.79	0.93
IPI00219725	CTNND1 Isoform 2AB of Catenin delta-1	QDVpYGPQPQVR	Y203	0	0.99	1.23
IPI00219725	CTNND1 Isoform 2AB of Catenin delta-1	SLDNNpYSTPNER	Y844	0	1.24	1.52
IPI00220032	CTNND2 Isoform 2 of Catenin delta-2	ASYAAGPASNpYADPYR	Y499	0	0.78	0.66
IPI00220032	CTNND2 Isoform 2 of Catenin delta-2	GGSAPEGATpYAAPR	Y292	0	0.14	0.71
IPI00029601	CTTN Src substrate cortactin	GPVSGTEPEPVpYSMEAADYR	Y446	0	1.09	0.90
IPI00029601	CTTN Src substrate cortactin	LPSSPVpYEDAASFK	Y421	0	1.83	1.18
IPI00029601	CTTN Src substrate cortactin	TQpTPPVSPAPQPTEERLPSSPVpYEDAASFK	T400, Y421	0	1.31	0.69
IPI00019146	CXADR Isoform 1 of Coxsackievirus and adenovirus receptor precursor	SYIGSNHSSLGSMSPSNMEGpYSK	Y313	0	0.74	0.98
IPI00019146	CXADR Isoform 1 of Coxsackievirus and adenovirus receptor precursor	TQpYNQVPSEDFER	Y318	0	1.03	0.53
IPI00026889	DAB1 Isoform DAB555 of Disabled homolog 1	EGVpYDVPK	Y232	0	1.64	1.40
IPI00337612	DCBLD1 Discoidin, CUB and LCCL domain-containing protein 1 precursor	AHTFSAQSGpYRVPGPQPGHK	Y662	0	-0.18	-0.27
IPI00337612	DCBLD1 Discoidin, CUB and LCCL domain-containing protein 1 precursor	AVSALATESGHPDSQKPPTHPGTSDSpYSAPR	Y737	0	-1.04	-1.09
IPI00419836	DCBLD2 Isoform 1 of Discoidin, CUB and LCCL domain-containing protein 2	AGKPGLPAPDELVpYQVPQSTQEVSGAGR	Y791	0	-0.28	-0.21
IPI00396435	DHX15 Putative pre-mRNA-splicing factor ATP-dependent RNA helicase DH	HRLDLGEDpYPSGK	Y13	0	1.11	0.97
IPI00023343	DLG3 Disks large homolog 3	DNEVDGQDpYHFVVSR	Y673	0	0.26	1.14
IPI00257508	DPYSL2 Dihydropyrimidinase-related protein 2	GLpYDGPVCEVSVTPK	Y499	0	1.14	0.77
IPI00014344	DYRK1A Isoform Long of Dual specificity tyrosine-phosphorylation-regulat	KVYNDGYDDDNpYDYIVK	Y145	0	0.03	-0.04
IPI00000352	DYRK1B Isoform 1 of Dual specificity tyrosine-phosphorylation-regulated k	IYQpYIQSR	Y273	0	-0.44	-0.48
IPI00022521	DYRK2 Isoform 2 of Dual specificity tyrosine-phosphorylation-regulated ki	VYTpYIQSR	Y309	0	-0.19	-0.29
IPI00641459	EEF1A1 Similar to Elongation factor 1 alpha	EHALLApYTLGVK	Y141	0	-0.02	-0.06
IPI00641459	EEF1A1 Similar to Elongation factor 1 alpha	STTTGHLIpYK	Y29	0	0.73	1.26
IPI00024307	EFNB1 Ephrin-B1 precursor	TTENNYCPHpYEK	Y317	0	2.06	2.33
IPI00024307	EFNB1 Ephrin-B1 precursor	VSGDpYGHPVYIVQEMPPQSPANIYYKV	Y324	0	1.32	1.05
IPI00011652	EFS Isoform Efs1 of Embryonal Fyn-associated substrate	DALEVpYDVPPTALR	Y148	0	0.79	0.36
IPI00011652	EFS Isoform Efs1 of Embryonal Fyn-associated substrate	VPSSGPpYDCPASFSHPLTR	Y163	0	1.17	0.52
IPI00018274	EGFR Isoform 1 of Epidermal growth factor receptor precursor	GSHQISLDNPDpYQQDFFPK	1172	0	1.20	0.84
IPI00016910	EIF3C Eukaryotic translation initiation factor 3 subunit 8	QGTpYGGYFR	Y881	0	0.60	0.65
IPI00165477	ELP3 Isoform 1 of Elongator complex protein 3	NLHDALSGHTSNNIpYEAVK	Y202	0	-0.04	-0.92
IPI00032003	EMD Emerin	GYNDDpYYEESYFTTR	Y94	0	1.36	1.18
IPI00465248	ENO1 Isoform alpha-enolase of Alpha-enolase	AAVPSGASTGIpYEALELR	Y44	0	0.00	0.15
IPI00294250	EPHA1 Ephrin type-A receptor 1 precursor	LLDDFDGTpYETQGGK	Y781	0	1.39	1.46
IPI00021267	EPHA2 Ephrin type-A receptor 2 precursor	QSPEDVpYFSK	Y575	0	0.82	0.94
IPI00021267	EPHA2 Ephrin type-A receptor 2 precursor	SEQLKPLKpTYVDPHTpYEDPNQAVLK	T587, Y594	0	0.95	1.22
IPI00021267	EPHA2 Ephrin type-A receptor 2 precursor	TpYVDPHTYEDPNQAVLK	Y588	0	0.06	0.15
IPI00021267	EPHA2 Ephrin type-A receptor 2 precursor	VLEDDPEATpYTTSGGK	Y772	0	1.21	1.11
IPI00008318	EPHA4 Ephrin type-A receptor 4 precursor	TYVDPFTpYEDPNQAVR	Y602	0	0.11	0.34
IPI00008318	EPHA4 Ephrin type-A receptor 4 precursor	VLEDDPEAApYTTR	Y779	0	0.75	0.76
IPI00016645	EPHA7 Isoform 1 of Ephrin type-A receptor 7 precursor	TpYIDPETYEDPNRAVHQFAK	Y608	0	0.05	0.37

IPI00016645	EPHA7 Isoform 1 of Ephrin type-A receptor 7 precursor	TYIDPETpYEDPNR	Y614	0	0.80	0.76
IPI00016645	EPHA7 Isoform 1 of Ephrin type-A receptor 7 precursor	VIEDDPEAVpYTTTGGKIPVR	Y791	0	0.71	0.73
IPI00008315	EPHB1 Isoform 1 of Ephrin type-B receptor 1 precursor	EAVpYSDKLQHYSTGR	Y575	0	0.99	0.61
IPI00008315	EPHB1 Isoform 1 of Ephrin type-B receptor 1 precursor	YLQDDTSDPTpYTSSLGGKIPVR	Y778	0	0.40	0.34
IPI00021275	EPHB2 Isoform 1 of Ephrin type-B receptor 2 precursor	FLEDDTSDPTpYTSALGGK	Y780	0	0.35	0.21
IPI00021275	EPHB2 Isoform 1 of Ephrin type-B receptor 2 precursor	IYIDPFTpYEDPNEAVR	Y602	0	-0.26	-0.01
IPI00289329	EPHB3 Ephrin type-B receptor 3 precursor	FLEDDPSDPTpYTSSLGGK	Y792	0	0.51	0.33
IPI00289329	EPHB3 Ephrin type-B receptor 3 precursor	VYIDPFTpYEDPNEAVR	Y614	0	1.22	1.19
IPI00186826	EPHB4 Ephrin receptor	EAEpYSDKHGQYLIGHGTK	Y574	0	1.79	1.90
IPI00186826	EPHB4 Ephrin receptor	FLEENSSDPTpYTSSLGGK	Y774	0	1.73	1.47
IPI00186826	EPHB4 Ephrin receptor	SQAKPGTPGGTGGPAPQpY	Y935	0	1.73	1.95
IPI00300384	ERBB2 Receptor tyrosine-protein kinase erbB-2 precursor	LLDIDETEpYHADGGKVPIK	Y877	0	2.96	1.69
IPI00438286	ERBB2IP Isoform 1 of Protein LAP2 (Ebrin)	AQIPEGDpYLSYR	Y1104	0	1.28	1.00
IPI00298285	ERBB3 Isoform 1 of Receptor tyrosine-protein kinase erbB-3 precursor	AFQGPGHQAPHVHpYAR	Y1307	0	2.05	2.48
IPI00001754	F11R Junctional adhesion molecule A precursor	VIpYSQPSAR	Y280	0	0.99	2.07
IPI00171499	FAM59A Isoform 3 of Protein FAM59A	QWTTITAHSLEEGHpYVIGPK	Y74	0	1.70	1.12
IPI00029263	FER Proto-oncogene tyrosine-protein kinase FER	QEDGGVpYSSSGLK	Y714	0	2.17	1.85
IPI00005142	FGFR1 Isoform 1 of Basic fibroblast growth factor receptor 1 precursor	DIHHIDpYpYKK	Y653; Y654	0	1.68	2.26
IPI00005142	FGFR1 Isoform 1 of Basic fibroblast growth factor receptor 1 precursor	DIHHIDpYYK	Y653	0	1.82	0.97
IPI00005142	FGFR1 Isoform 1 of Basic fibroblast growth factor receptor 1 precursor	RPPGLEpYCYNPSHNPEEQLSSK	Y583	0	1.67	1.69
IPI00010680	FGFR2 Isoform 1 of Fibroblast growth factor receptor 2 precursor	DINNIDpYpYKK	Y656. Y657	0	1.28	1.42
IPI00010680	FGFR2 Isoform 1 of Fibroblast growth factor receptor 2 precursor	DINNIDpYYKK	Y656	0	-0.36	-1.67
IPI00010680	FGFR2 Isoform 1 of Fibroblast growth factor receptor 2 precursor	RPPGMEpYSYDINRVPEEQMTFK	Y586	0	1.29	1.22
IPI00027174	FGFR3 Isoform 1 of Fibroblast growth factor receptor 3 precursor	DVHNLDpYYKK	Y647	0	1.19	0.97
IPI00304578	FGFR4 Fibroblast growth factor receptor 4 precursor	GVHHIDpYYKK	Y642	0	1.44	2.49
IPI00784186	FLJ32810 similar to Oligophrenin 1 isoform 1	LDTASSNGpYQRPGSVVAAK	Y405	0	-0.26	-0.07
IPI00027438	FLOT1 Flotillin-1	VSAQpYLSEIEMAK	Y203	0	-0.07	-0.05
IPI00644865	FRMD4A FERM domain containing 4A	SLESDQEGHpYSVK	Y861	0	0.50	0.59
IPI00031068	GAB1 Isoform 1 of GRB2-associated-binding protein 1	APSASVDSSLpYNLPR	Y259	0	0.72	1.29
IPI00031068	GAB1 Isoform 1 of GRB2-associated-binding protein 1	DASSQDCpYDIPR	Y406	0	0.89	1.65
IPI00031068	GAB1 Isoform 1 of GRB2-associated-binding protein 1	SSGSGSSVADERVDpYVVVDQQK	Y659	0	2.90	3.76
IPI00186990	GAB2 Isoform 1 of GRB2-associated-binding protein 2	ASSCETpYEYPQR	Y409	0	1.66	0.80
IPI00186990	GAB2 Isoform 1 of GRB2-associated-binding protein 2	GSLTGSETDNEDVpYTFK	Y293	0	1.27	0.45
IPI00186990	GAB2 Isoform 1 of GRB2-associated-binding protein 2	HNTEFRDSTpYDLPR	Y266	0	0.82	0.13
IPI00218487	GJA1 Gap junction alpha-1 protein	QASEQNWANpYSAEQNR	Y313	0	0.68	0.77
IPI00218487	GJA1 Gap junction alpha-1 protein	SDPpYHATSGALSPAK	Y247	0	0.30	0.14
IPI00004901	GPRC5C CDNA FLJ20242 fis, clone COLF6369	VPSEGApYDILPR	Y113	0	1.09	0.79
IPI00099883	GPRC5C G-protein coupled receptor family C group 5 member C precursor	NSQVFRNPpYVWD	Y438	0	0.94	1.02
IPI00334715	GRLF1 Isoform 1 of Glucocorticoid receptor DNA-binding factor 1	NEEENIpYSVPHDSTQGK	Y1105	0	0.09	0.00
IPI00334715	GRLF1 Isoform 1 of Glucocorticoid receptor DNA-binding factor 1	SVSSSPWLPQDGFDPSDpYAEPMDAVVKPR	Y1087	0	0.69	0.75
IPI00028570	GSK3B Isoform 1 of Glycogen synthase kinase-3 beta	GEPNVSpYICpSR	Y216, S219	0	0.01	-0.01
IPI00028570	GSK3B Isoform 1 of Glycogen synthase kinase-3 beta	GEPNVSpYICSR	Y216	0	0.06	0.09
IPI00219757	GSTP1 Glutathione S-transferase P	YISLIpYTNYEAGKDDYVK	Y109	0	0.19	0.23
IPI00054004	hCG_1790474 Uncharacterized protein ENSP00000354428	YFDSGDpYNMAK	Y65	0	2.48	1.69
IPI00029769	HCK Isoform p59-HCK of Tyrosine-protein kinase HCK	TLDNGGFpYISPR	Y188	0	-0.34	-0.24
IPI00006176	HGS Hepatocyte growth factor-regulated tyrosine kinase substrate	pYKVVQDTYQIMK	Y125	0	0.56	-0.17
IPI00006176	HGS Hepatocyte growth factor-regulated tyrosine kinase substrate	VVQDTpYQIMK	Y132	0	0.30	-0.07
IPI00215949	HIPK2 Isoform 2 of Homeodomain-interacting protein kinase 2	AVCSTpYLQpSR	Y361, S364	0	0.42	0.12
IPI00215949	HIPK2 Isoform 2 of Homeodomain-interacting protein kinase 2	AVCSTpYLQSR	Y361	0	0.04	0.14
IPI00099522	HIPK3 Isoform 1 of Homeodomain-interacting protein kinase 3	TVCSTpYLQSR	Y359	0	-2.59	-1.94
IPI00018534	HIST1H2BL Histone H2B type 1-L	ESYSVYVpYK	Y43	0	0.06	0.06
IPI00453473	HIST1H4A;HIST1H4K;HIST1H4J;HIST1H4D;HIST1H4E;HIST1H4H;HIST1H4C;	ISGLIpYEETR	Y52	0	-0.10	0.02
IPI00215965	HNRNPA1 Isoform A1-B of Heterogeneous nuclear ribonucleoprotein A1	NQGGpYGGSSSSSSYGSGR	Y357	0	1.65	3.12
IPI00215965	HNRNPA1 Isoform A1-B of Heterogeneous nuclear ribonucleoprotein A1	SSGPYGGGGQpYFAKPR	Y347	0	2.02	1.97
IPI00419373	HNRPA3 Isoform 1 of Heterogeneous nuclear ribonucleoprotein A3	SSGSPYGGGpYGSGGGSGGYGSR	Y364	0	1.49	1.50
IPI00013877	HNRPH3 Isoform 1 of Heterogeneous nuclear ribonucleoprotein H3	DGMDNQGGpYGSVGR	Y296	0	1.33	1.26
IPI00005038	HRSP12 Ribonuclease UK114	AApYQVAALPK	Y110	0	-0.27	-0.35
IPI00784154	HSPD1 60 kDa heat shock protein, mitochondrial precursor	GYISPpYFINTSK	Y241	0	-0.08	0.24
IPI00027232	IGF1R Insulin-like growth factor 1 receptor precursor	ASFDERQPpYAHMNGGR	Y1346	0	2.76	2.19
IPI00016932	INPPL1 Isoform 1 of Phosphatidylinositol-3,4,5-trisphosphate 5-phosphata	TLSEVDpYAPAGPAR	Y1168	0	0.73	0.63
IPI00025803	INSR Insulin receptor	DIpYETDpYYRK	Y1205, Y1209	0	1.93	1.42
IPI00025803	INSR Insulin receptor	DIpYETDYYRK	Y1205	0	0.74	0.28
IPI00025803	INSR Insulin receptor	DIYETDpYpYRK	Y1209, Y1210	0	1.86	1.21
IPI00025803	INSR Insulin receptor	DIYETDpYYR	Y1209	0	0.79	0.60
IPI00464978	IRS2 Insulin receptor substrate 2 insertion mutant (Fragment)	APpYTCGGDSDQYVLMSSPVGR	Y816	0	1.22	0.52
IPI00464978	IRS2 Insulin receptor substrate 2 insertion mutant (Fragment)	ApSSPAESSPEDSGpYMR	S731, Y744	0	1.47	1.06
IPI00464978	IRS2 Insulin receptor substrate 2 insertion mutant (Fragment)	APYTCGGDSDQpYVLMSSPVGR	Y825	0	1.21	0.63
IPI00464978	IRS2 Insulin receptor substrate 2 insertion mutant (Fragment)	ASSPAESSPEDSGpYMR	Y744	0	0.89	0.10
IPI00464978	IRS2 Insulin receptor substrate 2 insertion mutant (Fragment)	GVPGCCpYSSLPR	Y805	0	1.88	0.77
IPI00464978	IRS2 Insulin receptor substrate 2 insertion mutant (Fragment)	SDDpYMPMSPASVSAPK	Y677	0	1.07	0.39
IPI00464978	IRS2 Insulin receptor substrate 2 insertion mutant (Fragment)	QRPVPQPSSASLDEpYTLMR	Y600	0	0.18	-1.56
IPI00464978	IRS2 Insulin receptor substrate 2 insertion mutant (Fragment)	SPLSDpYMNLDFSSPK	Y980	0	1.83	-0.19
IPI00464978	IRS2 Insulin receptor substrate 2 insertion mutant (Fragment)	VAYHPYPEDpYGDIEIGSHR	Y634	0	2.78	1.79
IPI00216423	ITSN2 Isoform 4 of Intersectin-2	LIpYLVPEK	Y552	0	0.51	0.65
IPI00021396	KDR Vascular endothelial growth factor receptor 2 precursor	FHpYDNTAGISQYLQNSK	Y1214	0	2.29	1.75
IPI00103018	KIAA1217 Uncharacterized protein KIAA1217	NEGFpYADPYLYHEGR	Y393	0	0.96	0.97
IPI00103018	KIAA1217 Uncharacterized protein KIAA1217	NVpYYELNDVR	Y244	0	-0.13	-0.11
IPI00455851	KIAA1688 KIAA1688 protein	SGDpYSTMEGPELR	Y448	0	-0.24	0.13
IPI00470360	KIRREL Isoform 1 of Kin of IRRE-like protein 1 precursor	AVLpYADYR	Y622	0	1.81	3.93
IPI00470360	KIRREL Isoform 1 of Kin of IRRE-like protein 1 precursor	TPYEApYDPIGK	Y724	0	0.96	0.88
IPI00253050	Y	EIPFSpYLVGDSGK	Y513	0	1.11	1.29
IPI00014172	LAPTM4A Lysosomal-associated transmembrane protein 4A	MPEKEPPPPpYLPA	Y230	0	0.72	0.69
IPI00394952	LCK Isoform Long of Proto-oncogene tyrosine-protein kinase LCK	LIEDNEpYTAR	Y499	0	0.26	0.04
IPI00394952	LCK Isoform Long of Proto-oncogene tyrosine-protein kinase LCK	NLDNGGFpYISPR	Y192	0	-0.32	-0.12
IPI00394952	LCK Isoform Long of Proto-oncogene tyrosine-protein kinase LCK	SVLEDFFTATEGQpYQPQP	Y593	0	0.14	0.42
IPI00217966	LDHA Isoform 1 of L-lactate dehydrogenase A chain	EVHKQVVESApYEVIK	Y268	0	0.02	0.00

IPI00219217	LDHB L-lactate dehydrogenase B chain	MVVESApYEVIK	Y240	0	0.03	0.46
IPI00017562	LPHN2 Isoform 2 of Latrophilin-2 precursor	SENEDIpYYK	Y1350	0	0.76	0.37
IPI00023704	LPP Lipoma-preferred partner	NDSDPTpYGQQGHPNTWK	Y317	0	1.56	1.45
IPI00023704	LPP Lipoma-preferred partner	YYEGYpYAAGPGYGGR	Y301	0	1.34	1.39
IPI00478892	LRIG2 Leucine-rich repeats and immunoglobulin-like domains protein 2 pr	VICSDCYDNANIpYSR	Y912	0	1.55	1.31
IPI00328218	LSR Isoform 2 of Lipolysis-stimulated lipoprotein receptor	AATSGVPSIpYAPSTYAHLSPAK	Y286	0	0.15	0.24
IPI00328218	LSR Isoform 2 of Lipolysis-stimulated lipoprotein receptor	CCCPEALpYAAGK	Y272	0	0.60	0.31
IPI00328218	LSR Isoform 2 of Lipolysis-stimulated lipoprotein receptor	VLpYYMEK	Y348	0	0.65	0.45
IPI00298625	LYN Isoform LYN A of Tyrosine-protein kinase Lyn	SLDNGGpYYISPR	Y193	0	-0.37	-0.23
IPI00298625	LYN Isoform LYN A of Tyrosine-protein kinase Lyn	VIEDNEpYTAR	Y397	0	1.00	0.88
IPI00165946	MAGI1 Isoform 2 of Membrane-associated guanylate kinase, WW and PDZ	IEDPVpYGIYYVDHINR	Y413	0	0.41	0.55
IPI00165946	MAGI1 Isoform 2 of Membrane-associated guanylate kinase, WW and PDZ	IEDPVYGIpYYVDHINRK	Y416	0	0.29	0.44
IPI00170865	MAGI3 Membrane-associated guanylate kinase, WW and PDZ domain-con	IEDPQYGTpYYVDHLNQK	Y396	0	-0.04	-0.80
IPI00003479	MAPK1 Mitogen-activated protein kinase 1	VADPDHDHpTGFLTEpYVATR	Y187	0	1.41	2.31
IPI00019473	MAPK11 Mitogen-activated protein kinase 11	QADEEMTGpYVATR	T176, Y182	0	0.44	1.00
IPI00296283	MAPK12 Mitogen-activated protein kinase 12	QADSEMTGpYVVTR	Y185	0	1.33	1.02
IPI00005741	MAPK13 Mitogen-activated protein kinase 13	HADAEMTGpYVVTR	Y182	0	1.74	1.44
IPI00002857	MAPK14 Isoform CSBP2 of Mitogen-activated protein kinase 14	HTDDEMpTGpYVATR	Y182	0	0.13	0.71
IPI00002857	MAPK14 Isoform CSBP2 of Mitogen-activated protein kinase 14	HTDDEMTGpYVATR	Y182	0	0.81	1.13
IPI00018195	MAPK3 Mitogen-activated protein kinase 3	IADPEHDHpTGFLTEpYVATR	Y204	0	1.17	2.29
IPI00018195	MAPK3 Mitogen-activated protein kinase 3	IADPEHDHTGFLpTEpYVATR	T198, Y204	0	0.98	2.06
IPI00018195	MAPK3 Mitogen-activated protein kinase 3	IADPEHDHTGFLTEpYVATR	T202, Y204	0	1.26	1.13
IPI00149048	MAPK7 mitogen-activated protein kinase 7 isoform 2	GLCTSPAEHQYFMpTEpYVATR	Y82	0	0.86	2.60
IPI00149048	MAPK7 mitogen-activated protein kinase 7 isoform 2	GLCTSPAEHQYFMTEpYVATR	Y82	0	0.37	2.55
IPI00003145	MAPK8 Isoform 1 of Mitogen-activated protein kinase 8	TAGTSFMMTPpYVVTR	Y185	0	-0.03	-0.20
IPI00024673	MAPK9 Isoform Alpha-2 of Mitogen-activated protein kinase 9	TACTNFMMTPpYVVTR	Y185	0	0.30	0.27
IPI00064607	MEGF10 Isoform 1 of Multiple epidermal growth factor-like domains 10 pr	DSPpYAEINNSTSANR	Y1061	0	2.40	1.92
IPI00064607	MEGF10 Isoform 1 of Multiple epidermal growth factor-like domains 10 pr	LSQDPpYDLPK	Y1099	0	2.36	1.61
IPI00064607	MEGF10 Isoform 1 of Multiple epidermal growth factor-like domains 10 pr	NSHIPCHpYDLLPVR	Y1111	0	2.44	1.82
IPI00064607	MEGF10 Isoform 1 of Multiple epidermal growth factor-like domains 10 pr	SSECGpYVEMK	Y1048	0	2.82	2.45
IPI00643785	MLLT4 Myeloid/lymphoid or mixed-lineage leukemia (Trithorax homolog,	YSLpYEVHVSGEER	Y76	0	-0.35	-0.14
IPI00022558	MPZL1 Isoform 1 of Myelin protein zero-like protein 1 precursor	SESVVpYADIR	Y263	0	0.62	0.81
IPI00022558	MPZL1 Isoform 1 of Myelin protein zero-like protein 1 precursor	SLPSGSHQGPVIpYAQLDHSGGHHSDK	Y241	0	0.69	0.86
IPI00397526	MYH10 Isoform 1 of Myosin-10	ALApYDKLEK	Y1415	0	0.35	0.30
IPI00397526	MYH10 Isoform 1 of Myosin-10	AVIpYNPATQADWTAK	Y22	0	1.04	1.16
IPI00289258	MYO10 Myosin-X	CSVGTpYNSSGAYR	Y1164	0	1.61	0.46
IPI00004500	N4BP3 NEDD4-binding protein 3	NEPADpYATLYYR	Y83	0	-0.31	-0.51
IPI00031407	NEDD9 Enhancer of filamentation 1	TGHGYVpYEYPSR	Y166	0	-0.17	0.01
IPI00101049	NMD3 CGI-07 protein	LISQDIHSNTpYNYK	Y236	0	2.04	1.56
IPI00003373	OCLN Occludin	FYPESSpYK	Y342	0	1.26	1.20
IPI00003373	OCLN Occludin	SNILWDKEHIpYDEQPPNVEEWVK	Y287	0	-0.42	0.16
IPI00045423	PARD3 Isoform 7 of Partitioning-defective 3 homolog	DVTIGGSAPIpYVK	Y489	0	-0.01	-0.11
IPI00045423	PARD3 Isoform 7 of Partitioning-defective 3 homolog	EGHMMDALpYAQVK	Y1044	0	-0.26	-0.41
IPI00045423	PARD3 Isoform 7 of Partitioning-defective 3 homolog	FSPDSQpYIDNR	Y388	0	0.01	-0.04
IPI00045423	PARD3 Isoform 7 of Partitioning-defective 3 homolog	ISHSLpYSGIEGLDESPSR	Y706	0	-0.08	0.07
IPI00007935	PDLIM5 PDZ and LIM domain protein 5	YTEFpYHVPTHSDASK	Y251	0	0.88	0.75
IPI00292056	PIK3C2B Phosphatidylinositol-4-phosphate 3-kinase C2 domain-containing	LLGSVDpYDGINDAITR	Y228	0	1.30	1.54
IPI00031386	PIK3CA Phosphatidylinositol-4,5-bisphosphate 3-kinase catalytic subunit a	EAGFSpYSHAGLSNR	Y564	0	2.62	0.63
IPI00011736	PIK3R2 Phosphatidylinositol 3-kinase regulatory subunit beta	EYDQLpYEEYTR	Y464	0	-0.16	-0.05
IPI00005264	PKP2 Isoform 2 of Plakophilin-2	AHYTHSDpYQYSQR	Y166	0	1.32	1.99
IPI00026952	PKP3 Plakophilin-3	GQpYHTLQAGFSSR	Y84	0	1.22	1.02
IPI00021076	PKP4 Isoform Long of Plakophilin-4	LQHQQLpYYSQDDSNRK	Y1115	0	0.98	1.31
IPI00021076	PKP4 Isoform Long of Plakophilin-4	LYLQSPHSpYEDPYFDDR	Y1139	0	0.97	0.83
IPI00021076	PKP4 Isoform Long of Plakophilin-4	NNpYALNTTATYAEPYRPIQYR	Y470	0	1.06	1.09
IPI00021076	PKP4 Isoform Long of Plakophilin-4	SAVSPDLHITPIpYEGR	Y415	0	0.25	0.44
IPI00021076	PKP4 Isoform Long of Plakophilin-4	SPNHGTVELQGSQTALpYR	Y443	0	0.80	0.73
IPI00021076	PKP4 Isoform Long of Plakophilin-4	STTNpYVDFYSTK	Y1168	0	0.96	0.84
IPI00021076	PKP4 Isoform Long of Plakophilin-4	TVHDMEQFGQQQpYDIYER	Y369	0	0.90	0.99
IPI00021076	PKP4 Isoform Long of Plakophilin-4	TVHDMEQFGQQQYDIpYER	Y372	0	0.41	0.78
IPI00021076	PKP4 Isoform Long of Plakophilin-4	TYpYSPVYR	Y421	0	0.63	0.62
IPI00016736	PLCG1 1-phosphatidylinositol-4,5-bisphosphate phosphodiesterase gamm	ACpYRDMSSFPETK	Y1018	0	0.11	0.30
IPI00016736	PLCG1 1-phosphatidylinositol-4,5-bisphosphate phosphodiesterase gamm	IGTAEPDpYGALYEGR	Y812	0	2.07	1.31
IPI00016736	PLCG1 1-phosphatidylinositol-4,5-bisphosphate phosphodiesterase gamm	LAEGSAYEEVPTSMMpYSENDISNSIK	Y522	0	0.06	1.13
IPI00016736	PLCG1 1-phosphatidylinositol-4,5-bisphosphate phosphodiesterase gamm	NPGFpYVEANPMPTFK	Y824	0	1.45	1.29
IPI00014898	PLEC1 Isoform 1 of Plectin-1	GYYSPpYSVSGSGSTAGSR	Y4615	0	2.00	1.94
IPI00007248	PLEKHA6 Pleckstrin homology domain-containing family A member 6	SEDIpYADPAAYVMR	Y492	0	0.46	0.51
IPI00419933	PLEKHA7 Isoform 1 of Pleckstrin homology domain-containing family A me	QGPGQSLSFPENpYQTLPK	Y470	0	0.60	0.79
IPI00419933	PLEKHA7 Isoform 1 of Pleckstrin homology domain-containing family A me	SADDTpYLQLKK	Y656	0	0.25	0.03
IPI00439948	PPP1R13L Isoform 1 of RelA-associated inhibitor	TPLYLQPDApYGSLDR	Y132	0	0.61	1.52
IPI00739386	PRAGMIN Tyrosine-protein kinase SgK223	CLGLTGEPQPPAHPQEATQPEPIpYAESTK	Y413	0	0.50	0.51
IPI00739386	PRAGMIN Tyrosine-protein kinase SgK223	CPPApYTMVGLHNLEPR	Y159	0	0.07	0.10
IPI00739386	PRAGMIN Tyrosine-protein kinase SgK223	QEDAPVVpYLGSFR	Y132	0	0.57	0.71
IPI00013721	PRPF4B Serine/threonine-protein kinase PRP4 homolog	LCDFGSASHVADNDITPpYLVSR	Y849	0	0.17	0.30
IPI00219622	PSMA2 Proteasome subunit alpha type-2	HIGLVpYSGMGPDYR	Y76	0	-0.31	-0.70
IPI00012885	PTK2 Isoform 1 of Focal adhesion kinase 1	GSIDREDGSLQGPIGNQHIpYQPVGKPDPAAPPK	Y861	0	0.85	0.34
IPI00012885	PTK2 Isoform 1 of Focal adhesion kinase 1	pYMEDpSTYYKASK	Y570, S574	0	1.11	1.03
IPI00012885	PTK2 Isoform 1 of Focal adhesion kinase 1	pYMEDSTpYYKASK	Y570, Y576	0	1.27	0.92
IPI00012885	PTK2 Isoform 1 of Focal adhesion kinase 1	pYMEDSTYYKASK	Y570	0	0.96	0.94
IPI00012885	PTK2 Isoform 1 of Focal adhesion kinase 1	THAVpSVSETDDYAEIIDEEDTpYTMPSTR	S390, Y407	0	0.65	1.07
IPI00012885	PTK2 Isoform 1 of Focal adhesion kinase 1	YMEDpSTpYYKASK	Y576, Y574	0	0.22	-0.17
IPI00012885	PTK2 Isoform 1 of Focal adhesion kinase 1	YMEDSpTpYYKASK	T575, Y576	0	1.12	1.05
IPI00012885	PTK2 Isoform 1 of Focal adhesion kinase 1	YMEDSpTYpYK	T575, Y577	0	1.37	1.08
IPI00012885	PTK2 Isoform 1 of Focal adhesion kinase 1	YMEDSTpYpYKASK	Y576, Y577	0	1.28	0.93
IPI00012885	PTK2 Isoform 1 of Focal adhesion kinase 1	YMEDSTpYYK	Y576	0	1.31	0.97
IPI00298347	PTPN11 Isoform 2 of Tyrosine-protein phosphatase non-receptor type 11	IQNTGDpYYDLYGGEK	Y62	0	0.18	-0.11
IPI00221067	PTPRA Isoform 2 of Receptor-type tyrosine-protein phosphatase alpha pre	VVQEYIDAFSDpYANFK	Y789	0	0.89	1.03
IPI00107819	PTPRD PTPRD protein	AALEYLGSFDHpYAT	Y1504	0	2.07	1.50
IPI00023974	PTTG1IP Pituitary tumor-transforming gene 1 protein-interacting protein p	YGLFKEENPpYAR	Y174	0	1.31	0.95

IPI00220030	PXN Isoform Alpha of Paxillin	FIHQQPQSpSSPVpYGSSAK	S84, Y88	0	1.15	1.03
IPI00220030	PXN Isoform Alpha of Paxillin	VGEEEHVpYSFPNK	Y118	0	0.76	0.69
IPI00555917	PXN Paxillin variant (Fragment)	FIHQQPQSSpSPVpYGSSAK	S92, Y95	0	1.15	1.03
IPI00555917	PXN Paxillin variant (Fragment)	FIHQQPQSSSPVpYGSSAK	Y95	0	1.01	0.97
IPI00555917	PXN Paxillin variant (Fragment)	VGEEEHVpYSFPNKQK	Y125	0	0.44	0.70
IPI00829652	RBM16 Putative RNA-binding protein 16	TFNSELpYSLNDYKPPISK	Y12	0	0.22	0.30
IPI00013983	RET Proto-oncogene tyrosine-protein kinase receptor ret precursor	RPAQAFPVSpYSSSGAR	Y687	0	0.99	0.93
IPI00176662	Ribosomal protein S27	LVQSPNSpYFMDVK	Y31	0	0.16	0.02
IPI00014454	RIN1 Isoform RIN1 of Ras and Rab interactor 1	EKPAQDPLpYDVPNASGGQAGGPQRPGR	Y36	0	0.10	0.46
IPI00219798	ROBO1 Isoform 1 of Roundabout homolog 1 precursor	NGLTSTpYAGIR	Y932	0	0.64	0.49
IPI00008530	RPLP0 60S acidic ribosomal protein P0	IIQLLDDpYPK	Y24	0	0.07	-0.44
IPI00008438	RPS10 40S ribosomal protein S10	IAIpYELLFK	Y12	0	0.11	0.01
IPI00178324	SEMA6A CDNA FLJ14533 fis, clone NT2RM2000407, moderately similar to	MSEVAQMALEDQAATLEpYK	Y402	0	0.68	0.41
IPI00072377	SET Isoform 1 of Protein SET	IDFYFDENPpYFENK	Y146	0	0.50	0.35
IPI00010204	SFRS3 Splicing factor, arginine/serine-rich 3	AFGpYYGPLR	Y55	0	-0.01	-0.16
IPI00737545	SGK269 Tyrosine-protein kinase SgK269	VPIVINPNApYDNLAIYK	Y635	0	-0.26	-0.51
IPI00220490	SHANK2 Isoform 1 of SH3 and multiple ankyrin repeat domains protein 2	CFPAGSDMNSVpYER	Y227	0	2.03	1.59
IPI00220490	SHANK2 Isoform 1 of SH3 and multiple ankyrin repeat domains protein 2	ELDRYSLDSEDLpYSR	Y378	0	1.81	1.66
IPI00220490	SHANK2 Isoform 1 of SH3 and multiple ankyrin repeat domains protein 2	GQMPENPpYSEVGK	Y400	0	2.09	1.66
IPI00017578	SHB Shb	GESAGpYMEPYEAQR	Y355	0	1.93	1.88
IPI00017578	SHB Shb	LDpYCGGSGEPGGVQR	Y201	0	2.47	2.55
IPI00017578	SHB Shb	LPQDDDRPADEpYDQPWEWNR	Y423	0	1.16	1.50
IPI00017578	SHB Shb	VTIADDYpSDPFDAK	Y333	0	1.93	1.52
IPI00513796	SHC1 SHC (Src homology 2 domain containing) transforming protein 1	ELFDDPSpYVNVQNLDK	Y99	0	1.75	1.77
IPI00015180	SHROOM2 Protein Shroom2	TLSTSEQFpYSR	Y1257	0	0.74	0.93
IPI00007582	SLC6A8 Sodium- and chloride-dependent creatine transporter 1	SAENGIpYSVSGDEK	Y11	0	1.02	0.61
IPI00295698	SLC7A3 Cationic amino acid transporter 3	TVDLDPGTLpYVHSV	Y615	0	0.93	1.11
IPI00396130	SNIP Isoform 2 of p130Cas-associated protein	GEGLpYADPYGLLHEGR	Y456	0	0.27	0.40
IPI00306505	SPRY1 Portein sprouty homolog 1	GSNEpYTEGPSVVK	Y53	0	1.47	1.50
IPI00000878	TEC Tyrosine-protein kinase Tec	YVLDDQpYTSSSGAK	Y519	0	1.46	0.77
IPI00022462	TFRC Transferrin receptor protein 1	SAFSNLFGGEPLSpYTR	Y20	0	0.95	1.39
IPI00216219	TJP1 Isoform Long of Tight junction protein ZO-1	QLSpYFDRR	Y1361	0	0.29	0.41
IPI00216219	TJP1 Isoform Long of Tight junction protein ZO-1	SNHYDPEEDEEpYYRK	Y1354	0	0.37	0.70
IPI00335824	TJP1 Isoform Short of Tight junction protein ZO-1	FEEPAPLSpYDSRPR	Y1060	0	0.52	0.67
IPI00335824	TJP1 Isoform Short of Tight junction protein ZO-1	FTPKPpYTSSARPFER	Y1452	0	0.96	1.51
IPI00335824	TJP1 Isoform Short of Tight junction protein ZO-1	IDSPGFKPASQQVpYR	Y923	0	1.09	0.71
IPI00335824	TJP1 Isoform Short of Tight junction protein ZO-1	QYFEQpYSR	Y1115	0	0.36	0.54
IPI00003843	TJP2 Isoform A1 of Tight junction protein ZO-2	AYDPDpYER	Y338	0	0.78	1.17
IPI00003843	TJP2 Isoform A1 of Tight junction protein ZO-2	HQYSDpYDYHSSSEK	Y503	0	1.22	1.95
IPI00003843	TJP2 Isoform A1 of Tight junction protein ZO-2	IEIAQKHPDIpYAVPIK	Y1195	0	0.17	0.32
IPI00003843	TJP2 Isoform A1 of Tight junction protein ZO-2	SIDQDpYER	Y326	0	0.55	1.55
IPI00003843	TJP2 Isoform A1 of Tight junction protein ZO-2	TFLRPSPEDEAIpYGPNTK	Y583	0	0.10	0.57
IPI00642355	TLN1 Talin 1	TMQFEPSTMVpYDACR	Y26	0	0.00	0.04
IPI00298994	TLN1 Uncharacterized protein TLN1	ALDpYYMLR	Y70	0	-0.20	-0.18
IPI00552750	TNK2 Activated CDC42 kinase 1	VSSTHpYYLLPERPSYLER	Y913	0	1.52	1.22
IPI00307545	TNS1 Tensin-1	HPAGVpYQVSGLHNK	Y1254	0	1.29	1.35
IPI00654623	TNS3 CDNA FLJ13732 fis, clone PLACE3000145, moderately similar to TENS	KLSLGQpYDNDAGGQLPFSK	Y220	0	0.59	0.90
IPI00654623	TNS3 CDNA FLJ13732 fis, clone PLACE3000145, moderately similar to TENS	QQQMVVAHQpYSFAPDGEAR	Y41	0	0.48	0.90
IPI00301561	TRIP6 Thyroid receptor-interacting protein 6	QApYEPPPPPAYR	Y123	0	1.33	1.40
IPI00003923	UMPS Isoform 1 of Uridine 5'-monophosphate synthase	SGLSSPIpYIDLR	Y37	0	0.03	0.25
IPI00453476	Uncharacterized protein ENSP00000348237	FSGWpYDADLSPAGHEEAK	Y26	0	0.52	0.56
IPI00301058	VASP Vasodilator-stimulated phosphoprotein	VQIpYHNPTANSFR	Y39	0	-0.19	-0.02
IPI00291175	VCL Isoform 1 of Vinculin	NPGNQAApYEHFETMK	Y692	0	0.39	0.16
IPI00291175	VCL Isoform 1 of Vinculin	SFLDSGpYR	Y822	0	-0.24	-0.62
IPI00011676	WASL Neural Wiskott-Aldrich syndrome protein	VIpYDFIEK	Y256	0	1.05	0.56
IPI00025830	WEE1 Wee1-like protein kinase	SPAAPpYFLGSSFSPVR	Y132	0	3.64	2.85
IPI00013981	YES1 Proto-oncogene tyrosine-protein kinase Yes	GApYSLSIR	Y194	0	0.03	-0.23
IPI00013981	YES1 Proto-oncogene tyrosine-protein kinase Yes	LDNGGpYYITTR	Y222	0	-0.18	0.01
IPI00013981	YES1 Proto-oncogene tyrosine-protein kinase Yes	LIEDNEpYTARQGAK	Y425	0	-5.06	-3.64

* Ratios are log2 transformed . No change = 0; two fold up =1; and two fold down =-1

Highlighted in grey are peptides with two fold or greater change in either 5 min or 15 min FGF-2 stimulation
