# Supplementary material for: Tyrosine Phosphorylation Profiling in FGF-2 Stimulated Human Embryonic Stem Cells
Source: PLoS One. 2011 Mar 17;6(3):e17538. doi: 10.1371/journal.pone.0017538 (PMC3060089; doi:10.1371/journal.pone.0017538)
Supplement: Table S4 — List of phosphopeptides from the quantitated dataset. (PDF) [file pone.0017538.s007.pdf]

Cluster 1

IPI accession no.	Phosphopeptides	0 min	5 min	15 min	Protein
IPI00337612	AVSALATESGHPDSQKPPTHPGTSDSpYSAPR	0	-1.04	-1.09	DCBLD1 Discoidin, CUB and LCCL domain-containing protein 1 precursor
IPI00010680	DINNIDpYYKK	0	-0.36	-1.67	FGFR2 Isoform 1 of Fibroblast growth factor receptor 2 precursor
IPI00099522	TVCSTpYLQSR	0	-2.59	-1.94	HIPK3 Isoform 1 of Homeodomain-interacting protein kinase 3
IPI00464978	QRPVPQPSSASLDEpYTLMR	0	0.18	-1.56	IRS2 Insulin receptor substrate 2 insertion mutant (Fragment)

Cluster 2

IPI accession no.	Phosphopeptides	0 min	5 min	15 min	Protein
IPI00219725	HYEDGYPGGSDNpYGSLSR	0	1.04	1.21	CTNND1 Isoform 2AB of Catenin delta-1
IPI00219725	QDVpYGPQPQVR	0	0.99	1.23	CTNND1 Isoform 2AB of Catenin delta-1
IPI00023343	DNEVDGQDpYHFVVSR	0	0.26	1.14	DLG3 Disks large homolog 3
IPI00641459	STTTGHLIpYK	0	0.73	1.26	EEF1A1 Similar to Elongation factor 1 alpha
IPI00021267	SEQLKPLKpTYVDPHTpYEDPNQAVLK	0	0.95	1.22	EPHA2 Ephrin type-A receptor 2 precursor
IPI00031068	APSASVDSSLpYNLPR	0	0.72	1.29	GAB1 Isoform 1 of GRB2-associated-binding protein 1
IPI00031068	DASSQDCpYDIPR	0	0.89	1.65	GAB1 Isoform 1 of GRB2-associated-binding protein 1
IPI00099883	NSQVFRNPpYVWD	0	0.94	1.02	GPRC5C G-protein coupled receptor family C group 5 member C precursor
IPI00253050	EIPFSpYLVGDSGK	0	1.11	1.29	L1TD1 LINE-1 type transposase domain-containing protein 1
IPI00019473	QADEEMTGpYVATR	0	0.44	1.00	MAPK11 Mitogen-activated protein kinase 11
IPI00002857	HTDDEMTGpYVATR	0	0.81	1.13	MAPK14 Isoform CSBP2 of Mitogen-activated protein kinase 14
IPI00149048	GLCTSPAEHQYFMpTEpYVATR	0	0.86	2.60	MAPK7 mitogen-activated protein kinase 7 isoform 2
IPI00149048	GLCTSPAEHQYFMTEpYVATR	0	0.37	2.55	MAPK7 mitogen-activated protein kinase 7 isoform 2
IPI00397526	AVIpYNPATQADWTAK	0	1.04	1.16	MYH10 Isoform 1 of Myosin-10
IPI00021076	LQHQQLpYYSQDDSNRK	0	0.98	1.31	PKP4 Isoform Long of Plakophilin-4
IPI00021076	NNpYALNTTATYAEPYRPIQYR	0	1.06	1.09	PKP4 Isoform Long of Plakophilin-4
IPI00016736	LAEGSAYEEVPTSMMpYSENDISNSIK	0	0.06	1.13	PLCG1 1-phosphatidylinositol-4,5-bisphosphate phosphodiesterase gamma-1
IPI00439948	TPLYLQPDApYGSLDR	0	0.61	1.52	PPP1R13L Isoform 1 of RelA-associated inhibitor
IPI00012885	THAVpSVSETDDYAEIIDEEDTpYTMPSTR	0	0.65	1.07	PTK2 Isoform 1 of Focal adhesion kinase 1
IPI00221067	VVQEYIDAFSDpYANFK	0	0.89	1.03	PTPRA Isoform 2 of Receptor-type tyrosine-protein phosphatase alpha precursor
IPI00295698	TVDLDPGTLpYVHSV	0	0.93	1.11	SLC7A3 Cationic amino acid transporter 3
IPI00022462	SAFSNLFGGEPLSpYTR	0	0.95	1.39	TFRC Transferrin receptor protein 1
IPI00335824	FTPKPpYTSSARPFER	0	0.96	1.51	TJP1 Isoform Short of Tight junction protein ZO-1
IPI00003843	AYDPDpYER	0	0.78	1.17	TJP2 Isoform A1 of Tight junction protein ZO-2
IPI00003843	SIDQDpYER	0	0.55	1.55	TJP2 Isoform A1 of Tight junction protein ZO-2

Cluster 3

IPI accession no.	Phosphopeptides	0 min	5 min	15 min	Protein
IPI00219852	ADGGAEpYATYQTK	0	2.74	2.14	CD46 Isoform B of Membrane cofactor protein precursor
IPI00023530	IGEGTpYGTVFK	0	1.33	1.57	CDK5 Cell division protein kinase 5
IPI00014197	KTPQGPPEIpYSDTQFPSLQSTAK	0	1.98	1.50	CDV3 Isoform 1 of Protein CDV3 homolog
IPI00219725	SLDNNpYSTPNER	0	1.24	1.52	CTNND1 Isoform 2AB of Catenin delta-1
IPI00026889	EGVpYDVPK	0	1.64	1.40	DAB1 Isoform DAB555 of Disabled homolog 1
IPI00024307	TTENNYCPHpYEK	0	2.06	2.33	EFNB1 Ephrin-B1 precursor
IPI00294250	LLDDFDGTpYETQGGK	0	1.39	1.46	EPHA1 Ephrin type-A receptor 1 precursor
IPI00186826	EAEpYSDKHGQYLIGHGTK	0	1.79	1.90	EPHB4 Ephrin receptor
IPI00186826	FLEENSSDPTpYTSSLGGK	0	1.73	1.47	EPHB4 Ephrin receptor
IPI00186826	SQAKPGTPGGTGGPAPQpY	0	1.73	1.95	EPHB4 Ephrin receptor

IPI00300384	LLDIDETEpYHADGGKVPIK	0	2.96	1.69	ERBB2 Receptor tyrosine-protein kinase erbB-2 precursor
IPI00298285	AFQGPGHQAPHVHpYAR	0	2.05	2.48	ERBB3 Isoform 1 of Receptor tyrosine-protein kinase erbB-3 precursor
IPI00001754	VIpYSQPSAR	0	0.99	2.07	F11R Junctional adhesion molecule A precursor
IPI00029263	QEDGGVpYSSSGLK	0	2.17	1.85	FER Proto-oncogene tyrosine-protein kinase FER
IPI00005142	DIHHIDpYpYKK	0	1.68	2.26	FGFR1 Isoform 1 of Basic fibroblast growth factor receptor 1 precursor
IPI00005142	RPPGLEpYCYNPSHNPEEQLSSK	0	1.67	1.69	FGFR1 Isoform 1 of Basic fibroblast growth factor receptor 1 precursor
IPI00010680	DINNIDpYpYKK	0	1.28	1.42	FGFR2 Isoform 1 of Fibroblast growth factor receptor 2 precursor
IPI00304578	GVHHIDpYYKK	0	1.44	2.49	FGFR4 Fibroblast growth factor receptor 4 precursor
IPI00054004	YFDSGDpYNMAK	0	2.48	1.69	hCG_1790474 Uncharacterized protein ENSP00000354428
IPI00215965	SSGPYGGGGQpYFAKPR	0	2.02	1.97	HNRNPA1 Isoform A1-B of Heterogeneous nuclear ribonucleoprotein A1
IPI00419373	SSGSPYGGGpYGSGGGSGGYGSR	0	1.49	1.50	HNRPA3 Isoform 1 of Heterogeneous nuclear ribonucleoprotein A3
IPI00027232	ASFDERQPpYAHMNGGR	0	2.76	2.19	IGF1R Insulin-like growth factor 1 receptor precursor
IPI00025803	DIpYETDpYYRK	0	1.93	1.42	INSR Insulin receptor
IPI00464978	VAYHPYPEDpYGDIEIGSHR	0	2.78	1.79	IRS2 Insulin receptor substrate 2 insertion mutant (Fragment)
IPI00021396	FHpYDNTAGISQYLQNSK	0	2.29	1.75	KDR Vascular endothelial growth factor receptor 2 precursor
IPI00023704	NDSDPTpYGQQGHPNTWK	0	1.56	1.45	LPP Lipoma-preferred partner
IPI00023704	YYEGYpYAAGPGYGGR	0	1.34	1.39	LPP Lipoma-preferred partner
IPI00478892	VICSDCYDNANIpYSR	0	1.55	1.31	LRIG2 Leucine-rich repeats and immunoglobulin-like domains protein 2 precursor
IPI00003479	VADPDHDHpTGFLTEpYVATR	0	1.41	2.31	MAPK1 Mitogen-activated protein kinase 1
IPI00005741	HADAEMTGpYVVTR	0	1.74	1.44	MAPK13 Mitogen-activated protein kinase 13
IPI00018195	IADPEHDHpTGFLTEpYVATR	0	1.17	2.29	MAPK3 Mitogen-activated protein kinase 3
IPI00018195	IADPEHDHTGFLpTEpYVATR	0	0.98	2.06	MAPK3 Mitogen-activated protein kinase 3
IPI00064607	DSPpYAEINNSTSANR	0	2.40	1.92	MEGF10 Isoform 1 of Multiple epidermal growth factor-like domains 10 precursor
IPI00064607	LSQDPpYDLPK	0	2.36	1.61	MEGF10 Isoform 1 of Multiple epidermal growth factor-like domains 10 precursor
IPI00064607	NSHIPCHpYDLLPVR	0	2.44	1.82	MEGF10 Isoform 1 of Multiple epidermal growth factor-like domains 10 precursor
IPI00064607	SSECGpYVEMK	0	2.82	2.45	MEGF10 Isoform 1 of Multiple epidermal growth factor-like domains 10 precursor
IPI00101049	LISQDIHSNTpYNYK	0	2.04	1.56	NMD3 CGI-07 protein
IPI00292056	LLGSVDpYDGINDAITR	0	1.30	1.54	PIK3C2B Phosphatidylinositol-4-phosphate 3-kinase C2 domain-containing beta polypeptide
IPI00005264	AHYTHSDpYQYSQR	0	1.32	1.99	PKP2 Isoform 2 of Plakophilin-2
IPI00016736	IGTAEPDpYGALYEGR	0	2.07	1.31	PLCG1 1-phosphatidylinositol-4,5-bisphosphate phosphodiesterase gamma-1
IPI00016736	NPGFpYVEANPMPTFK	0	1.45	1.29	PLCG1 1-phosphatidylinositol-4,5-bisphosphate phosphodiesterase gamma-1
IPI00014898	GYYSPpYSVSGSGSTAGSR	0	2.00	1.94	PLEC1 Isoform 1 of Plectin-1
IPI00107819	AALEYLGSFDHpYAT	0	2.07	1.50	PTPRD PTPRD protein
IPI00220490	CFPAGSDMNSVpYER	0	2.03	1.59	SHANK2 Isoform 1 of SH3 and multiple ankyrin repeat domains protein 2
IPI00220490	ELDRYSLDSEDLpYSR	0	1.81	1.66	SHANK2 Isoform 1 of SH3 and multiple ankyrin repeat domains protein 2
IPI00220490	GQMPENPpYSEVGK	0	2.09	1.66	SHANK2 Isoform 1 of SH3 and multiple ankyrin repeat domains protein 2
IPI00017578	GESAGpYMEPYEAQR	0	1.93	1.88	SHB Shb
IPI00017578	LDpYCGGSGEPGGVQR	0	2.47	2.55	SHB Shb
IPI00017578	LPQDDDRPADEpYDQPWEWNR	0	1.16	1.50	SHB Shb
IPI00017578	VTIADDpYSDPFDAK	0	1.93	1.52	SHB Shb
IPI00513796	ELFDDPSpYVNVQNLDK	0	1.75	1.77	SHC1 SHC (Src homology 2 domain containing) transforming protein 1
IPI00306505	GSNEpYTEGPSVVK	0	1.47	1.50	SPRY1 Portein sprouty homolog 1
IPI00003843	HQYSDpYDYHSSSEK	0	1.22	1.95	TJP2 Isoform A1 of Tight junction protein ZO-2
IPI00552750	VSSTHpYYLLPERPSYLER	0	1.52	1.22	TNK2 Activated CDC42 kinase 1
IPI00307545	HPAGVpYQVSGLHNK	0	1.29	1.35	TNS1 Tensin-1

IPI00301561	QApYEPPPPPAYR	0	1.33	1.40	TRIP6 Thyroid receptor-interacting protein 6

Cluster 4

IPI accession no.	Phosphopeptides	0 min	5 min	15 min	Protein
IPI00395663	EEDEHPpYELLLTAETK	0	1.32	1.25	ANKS1A Ankyrin repeat and SAM domain-containing protein 1A
IPI00004497	GHGQPGADAEKPFpYVNVEFHHER	0	1.19	1.09	BCR Isoform 1 of Breakpoint cluster region protein
IPI00059185	HVHLENATEpYATLR	0	1.53	0.19	C11orf52 Uncharacterized protein C11orf52
IPI00016670	ALNGAEPNpYHSLPSAR	0	1.11	0.91	C11orf59 UPF0404 protein C11orf59
IPI00029601	GPVSGTEPEPVpYSMEAADYR	0	1.09	0.90	CTTN Src substrate cortactin
IPI00029601	LPSSPVpYEDAASFK	0	1.83	1.18	CTTN Src substrate cortactin
IPI00029601	TQpTPPVSPAPQPTEERLPSSPVpYEDAASFK	0	1.31	0.69	CTTN Src substrate cortactin
IPI00019146	TQpYNQVPSEDFER	0	1.03	0.53	CXADR Isoform 1 of Coxsackievirus and adenovirus receptor precursor
IPI00396435	HRLDLGEDpYPSGK	0	1.11	0.97	DHX15 Putative pre-mRNA-splicing factor ATP-dependent RNA helicase DHX15
IPI00257508	GLpYDGPVCEVSVTPK	0	1.14	0.77	DPYSL2 Dihydropyrimidinase-related protein 2
IPI00024307	VSGDpYGHPVYIVQEMPPQSPANIYYKV	0	1.32	1.05	EFNB1 Ephrin-B1 precursor
IPI00011652	VPSSGPpYDCPASFSHPLTR	0	1.17	0.52	EFS Isoform Efs1 of Embryonal Fyn-associated substrate
IPI00018274	GSHQISLDNPDpYQQDFFPK	0	1.20	0.84	EGFR Isoform 1 of Epidermal growth factor receptor precursor
IPI00032003	GYNDDpYYEESYFTTR	0	1.36	1.18	EMD Emerin
IPI00021267	VLEDDPEATpYTTSGGK	0	1.21	1.11	EPHA2 Ephrin type-A receptor 2 precursor
IPI00289329	VYIDPFTpYEDPNEAVR	0	1.22	1.19	EPHB3 Ephrin type-B receptor 3 precursor
IPI00438286	AQIPEGDpYLSYR	0	1.28	1.00	ERBB2IP Isoform 1 of Protein LAP2
IPI00171499	QWTTITAHSLEEGHpYVIGPK	0	1.70	1.12	FAM59A Isoform 3 of Protein FAM59A
IPI00005142	DIHHIDpYYK	0	1.82	0.97	FGFR1 Isoform 1 of Basic fibroblast growth factor receptor 1 precursor
IPI00010680	RPPGMEpYSYDINRVPEEQMTFK	0	1.29	1.22	FGFR2 Isoform 1 of Fibroblast growth factor receptor 2 precursor
IPI00027174	DVHNLDpYYKK	0	1.19	0.97	FGFR3 Isoform 1 of Fibroblast growth factor receptor 3 precursor
IPI00186990	ASSCETpYEYPQR	0	1.66	0.80	GAB2 Isoform 1 of GRB2-associated-binding protein 2
IPI00186990	GSLTGSETDNEDVpYTFK	0	1.27	0.45	GAB2 Isoform 1 of GRB2-associated-binding protein 2
IPI00004901	VPSEGApYDIILPR	0	1.09	0.79	GPRC5C CDNA FLJ20242 fis, clone COLF6369
IPI00013877	DGMDNQGGpYGSVGR	0	1.33	1.26	HNRPH3 Isoform 1 of Heterogeneous nuclear ribonucleoprotein H3
IPI00025803	DIYETDpYpYRK	0	1.86	1.21	INSR Insulin receptor
IPI00464978	APpYTCGGDSDQYVLMSSPVGR	0	1.22	0.52	IRS2 Insulin receptor substrate 2 insertion mutant (Fragment)
IPI00464978	ApSSPAESSPEDSGpYMR	0	1.47	1.06	IRS2 Insulin receptor substrate 2 insertion mutant (Fragment)
IPI00464978	APYTCGGDSDQpYVLMSSPVGR	0	1.21	0.63	IRS2 Insulin receptor substrate 2 insertion mutant (Fragment)
IPI00464978	GVPGCCpYSSLPR	0	1.88	0.77	IRS2 Insulin receptor substrate 2 insertion mutant (Fragment)
IPI00464978	SDDpYMPMSPASVSAPK	0	1.07	0.39	IRS2 Insulin receptor substrate 2 insertion mutant (Fragment)
IPI00464978	SPLSDpYMNLDFSSPK	0	1.83	-0.19	IRS2 Insulin receptor substrate 2 insertion mutant (Fragment)
IPI00298625	VIEDNEpYTAR	0	1.00	0.88	LYN Isoform LYN A of Tyrosine-protein kinase Lyn
IPI00296283	QADSEMTGpYVVTR	0	1.33	1.02	MAPK12 Mitogen-activated protein kinase 12
IPI00018195	IADPEHDHTGFLTEpYVATR	0	1.26	1.13	MAPK3 Mitogen-activated protein kinase 3
IPI00289258	CSVGTpYNSSGAYR	0	1.61	0.46	MYO10 Myosin-X
IPI00003373	FYPESSpYK	0	1.26	1.20	OCLN Occludin
IPI00031386	EAGFSpYSHAGLSNR	0	2.62	0.63	PIK3CA Phosphatidylinositol-4,5-bisphosphate 3-kinase catalytic subunit alpha isoform
IPI00026952	GQpYHTLQAGFSSR	0	1.22	1.02	PKP3 Plakophilin-3
IPI00012885	pYMEDpSTYYKASK	0	1.11	1.03	PTK2 Isoform 1 of Focal adhesion kinase 1
IPI00012885	pYMEDSTpYYKASK	0	1.27	0.92	PTK2 Isoform 1 of Focal adhesion kinase 1
IPI00012885	YMEDSpTpYYKASK	0	1.12	1.05	PTK2 Isoform 1 of Focal adhesion kinase 1

IPI00012885	YMEDSpTYpYK	0	1.37	1.08	PTK2 Isoform 1 of Focal adhesion kinase 1
IPI00012885	YMEDSTpYpYKASK	0	1.28	0.93	PTK2 Isoform 1 of Focal adhesion kinase 1
IPI00012885	YMEDSTpYYK	0	1.31	0.97	PTK2 Isoform 1 of Focal adhesion kinase 1
IPI00023974	YGLFKEENPpYAR	0	1.31	0.95	PTTG1IP Pituitary tumor-transforming gene 1 protein-interacting protein precursor
IPI00220030	FIHQQPQSpSSPVpYGSSAK	0	1.15	1.03	PXN Isoform Alpha of Paxillin
IPI00555917	FIHQQPQSSpSPVpYGSSAK	0	1.15	1.03	PXN Paxillin variant (Fragment)
IPI00555917	FIHQQPQSSSPVpYGSSAK	0	1.01	0.97	PXN Paxillin variant (Fragment)
IPI00007582	SAENGIpYSVSGDEK	0	1.02	0.61	SLC6A8 Sodium- and chloride-dependent creatine transporter 1
IPI00000878	YVLDDQpYTSSSGAK	0	1.46	0.77	TEC Tyrosine-protein kinase Tec
IPI00335824	IDSPGFKPASQQVpYR	0	1.09	0.71	TJP1 Isoform Short of Tight junction protein ZO-1
IPI00011676	VIpYDFIEK	0	1.05	0.56	WASL Neural Wiskott-Aldrich syndrome protein

Cluster 5

IPI accession no.	Phosphopeptides	0 min	5 min	15 min	Protein
IPI00031068	SSGSGSSVADERVDpYVVVDQQK	0	2.90	3.76	GAB1 Isoform 1 of GRB2-associated-binding protein 1
IPI00215965	NQGGpYGGSSSSSSYGSGR	0	1.65	3.12	HNRNPA1 Isoform A1-B of Heterogeneous nuclear ribonucleoprotein A1
IPI00470360	AVLpYADYR	0	1.81	3.93	KIRREL Isoform 1 of Kin of IRRE-like protein 1 precursor
IPI00025830	SPAAPpYFLGSSFSPVR	0	3.64	2.85	WEE1 Wee1-like protein kinase
