# Supplementary material for: Tyrosine Phosphorylation Profiling in FGF-2 Stimulated Human Embryonic Stem Cells
Source: PLoS One. 2011 Mar 17;6(3):e17538. doi: 10.1371/journal.pone.0017538 (PMC3060089; doi:10.1371/journal.pone.0017538)
Supplement: Text S1 — Supplementary Materials and Methods. (DOC) [file pone.0017538.s009.doc]

**Supplementary material and methods**

*Src Kinases Inhibition*

Human ESC were cultured as described in the Experimental Procedures with the exception that the SU6656 inhibitor (10 mM stock, Calbiochem, Darmstadt, Germany) was added daily for 6 days to a final concentration of 1, 2 and 4 µM starting 24 h after inoculation. Human ESCs were harvested by mechanical dissociation, and the total cell count was determined by the nuclei count method using NucleoCounter (Chemometec, Copenhagen, Denmark) as per the manufacturer’s recommendations

*Quantitative real-time polymerase chain reaction (qRT-PCR)*

Total RNA was extracted from hESC treated with SU6656 inhibitor, using the Nucleospin RNA II kit (Macherey-Nagel, Germany) according to the manufacturer’s protocol. Reverse transcription was carried out with 1µg of total RNA using M-MLV Reverse Transcriptase (Promega, Madison, USA). Quantitative real time PCR analysis was performed using an ABI PRISM 7500 Sequence Detection System and SYBR green PCR Master Mix (Applied Biosystems, California, USA). Primers used are listed in below. Fold induction was calculated relative to 18S rRNA expression using the ΔΔCt method. Nanog:  *Forward 5'-GAA AAA CAA CTG GCC GAA GAA T-3'; Reverse 5'-GGT GCT GAG GCC TTC TGC-3'.* EpCAM: *Forward 5’-GAA AAA GAT GTT AAA GGT GAA TCC TTG-3’; Reverse 5’-GTC AGG TCC ATT TTC TTA GAA TGA AAC-3’.* Oct3/4: *Forward 5’-AAA CCC GGA GGA GTC CCA G-3’; Reverse 5’-TGG CAA ATT GCT CGA GTT CTT-3’.* 18S: *Forward 5’-CGC CGC GCT CTA CCT TAC CTA-3’; Reverse 5’-TAG GAG AGG AGC GAG CGA CCA-3’*
